# Integrating ICT in education: A scoping review of pre-service teachers’ ICT beliefs

**DOI:** 10.1371/journal.pone.0317591

**Published:** 2025-02-07

**Authors:** Honghuan Li

**Affiliations:** School of Foreign Studies, China University of Political Science and Law, Beijing, China; National University of Lesotho, LESOTHO

## Abstract

This scoping review aims to map the current understanding of pre-service teachers’ beliefs about ICT, identify critical research gaps, and provide actionable insights for teacher education policy and practice. A comprehensive search of seven electronic databases yielded 1366 studies, of which 277 were included. The review identified seven key themes, including a predominant focus on general ICT beliefs and the limited exploration of cutting-edge technologies or regional variations. Quantitative methods dominated the research landscape, often employing standardized instruments like the Technology Attitude Scale. The findings highlight the need for greater emphasis on underrepresented regions, innovative technologies, and qualitative methodologies to deepen contextual understanding. This review offers a foundational resource for stakeholders in teacher education and sets the stage for future research to bridge identified gaps, ultimately enhancing the integration of ICT in teacher training and classroom practice.

## Introduction

In the evolving landscape of global education, the integration of Information and Communication Technology (ICT) has become a pivotal element in shaping teaching methodologies and educational outcomes. Its integration into teaching practices offers numerous benefits, enhancing both teaching and learning experiences. By encouraging innovative teaching methods, moving away from traditional blackboard and chalk approaches to more dynamic and interactive learning experiences, ICT tools can significantly improve student performance, motivation, and problem-solving skills by making learning more interactive and engaging. The use of ICT in classrooms can also enhance student learning experiences and outcomes [1, 2], and it can provide greater flexibility in teaching and learning, allowing for more personalized and accessible education. The proficiency and attitudes of pre-service teachers towards ICT are critical as they form the backbone of future educational delivery systems. The significance of understanding pre-service teachers’ beliefs about ICT lies in its potential to influence their readiness to integrate technology into classrooms effectively. These beliefs influence how prospective teachers will use ICT in their classrooms, impacting the quality of education and the success of technological integration. Significant gaps remain in addressing the diversity of methodologies and regional contexts in terms of pre-service teachers’ ICT beliefs. Therefore, understanding pre-service teachers’ ICT beliefs is vital for promoting effective teaching practices in the future and crucial for enhancing their future teaching practices. This scoping review adopts a systematic framework to bridge these gaps, providing insights into how beliefs shape teaching practices and identifying directions for future research and policy.

As a form of knowledge synthesis, a scoping review incorporates the process of mapping the existing literature or evidence base to identify patterns and themes in an area of research. In established fields where there is abundant evidence, a scoping review can provide a panoramic view and a holistic understanding of the previous literature. According to Arksey and O’Malley [3], Daudt, van Mossel and Scott [4], and Colquhoun, Levac [5], a scoping review aims to map key concepts rapidly on a particular topic or research area and to identify types of evidence and research gaps to inform research, practice, and policymaking, especially where a defined area or field is complex and has not been synthesized before. Thus, a scoping review can be employed for the following purpose: (1) to explore the extent of the literature in a particular domain with minimal data synthesis and without assessing the quality of included studies; (2) to summarize the research findings and identify the knowledge gaps; (3) to identify the potential scope of a systematic review by gathering a map or a snapshot of the existing literature; (4) to guide the priorities of future research, practice, and policymaking directions by presenting the synthesis outcomes.

The approach for this scoping review is underpinned by the Preferred Reporting Items for Systematic Reviews and Meta-Analyses (PRISMA) statement [6] and the framework developed by Arksey and O’Malley [3]. This approach adopts a rigorous process and holistic view of transparency during the scoping review, which enables the replication of the search strategy and increases the reliability of the findings (see S1 Table for PRISMA Checklist). It is organized in five distinct stages: (1) developing the initial research questions, (2) identifying the relevant studies, (3) selecting the studies with clear inclusion and exclusion criteria, (4) extracting and charting key information from the selected studies (without assessing risk of bias), and (5) summarizing and synthesizing the findings. The purpose of this work is to conduct a scoping review of the literature to map out the current understanding of pre-service teachers’ ICT beliefs, identify gaps in the research, and understand the breadth of methodologies employed in the measurement of ICT beliefs.

## Methods

### Review question development

To ensure that a scoping review generates and captures a sufficient breadth of coverage and substantial range of the literature relating to the topic of interest, the consideration and development of the research questions should cover all aspects of the research area. Articulating the research questions clearly is critical to the review process, as it will guide the subsequent stages of the review process and the structure of the report [7]. Prior to conducting the formal process of the literature search, the purpose of this study and research questions were established. The aims of the scoping review are to explore the extent of the existing literature with regard to pre-service teachers’ ICT beliefs, to identify the potential purview of a further systematic review, to identify the research gaps and to guide future empirical research. The main rationale behind this broad question is to identify the key aspects and research components involved in designing and conducting a piece of empirical research on pre-service teachers’ ICT beliefs in term of the trending technologies in the educational field. Therefore, based on the aims and rationale of this research, the following two core review questions were posited to guide this review:

1. What are the areas of focus and domains in the literature on pre-service teachers’ ICT beliefs?
2. What are the adopted methodologies used in the existing studies on pre-service teachers’ ICT beliefs?

### Identification of the relevant studies

In order to glean information and cover a broad range of the available literature, a wide definition of key terms and a variety of sources for searching and capturing the literature are recommended [3]. Key search terms and concepts were developed to identify the literature relating to pre-service teachers’ or prospective teachers’ beliefs or attitudes to ICT or technology tools. To maximize the permutations of the terms scoped, Boolean operators to combine, narrow, or widen the literature search and search tools to assist the searching process were adopted in the searching techniques. A university librarian was consulted to refine the key search terms, devise the search techniques, and identify the relevant databases in the educational field for optimized results. A search strategy was delineated, which would yield the identification of the most relevant literature.

The search terms were, necessarily, devised broadly and mapped based on thesauri since the goal was to conduct a broad, rather than specific, search of the literature. These terms were combined with Boolean operators to create search strings that could be useful to search in the electronic database: (ICT OR “Information and Communication Technology” OR technology OR computer* OR digital OR IT OR “information technology” OR Internet) AND (perception* OR belief* OR attitude* OR confidence) AND (“teacher education” OR pre-service OR “student teacher*” OR prospective).

The following seven electronic databases were searched: Web of Science, Scopus, ERIC, JSTOR, ScienceDirect, ProQuest Dissertation, and Google Scholar. Although different search engines or databases required different search tactics or different search string styles, the same keywords search technique was always followed in the search process. The linked search string developed in Scopus is outlined in Table 1.

**Table 1:** Search string example.

Search Strings in Scopus
(TITLE (ict OR "Information and Communication Technology" OR technology OR computer* OR digital OR it OR "information technology" OR internet) AND TITLE (perception* OR belief* OR attitude* OR confidence) AND TITLE ("teacher education" OR pre-service OR "student teacher*" OR prospective)) AND PUBYEAR > 2000

The inclusion and exclusion criteria were identified directly from the research questions to guide this scoping review and to help provide comprehensiveness and transparency in the identification of primary evidence. The time period for the considered literature was set to studies in the 21st century (from January 2000 to July 2024), which is considered appropriate for the time period of technology development. The focus on this 24-year period was intended to capture a significant era of technological advancement and its impact on educational paradigms, particularly regarding pre-service teachers’ ICT beliefs. In this scoping review, all the literature database searches were limited to English-language articles. The exclusion of non-English studies and pre-2000 literature was grounded in the focus on contemporary ICT developments that align with the rapid technological advancements of the 21st century. A full list of the inclusion and exclusion criteria is outlined in Table 2.

**Table 2:** Inclusion and exclusion criteria.

Criteria	Inclusion	Exclusion
**Type of Study**	Empirical studies reporting quantitative or qualitative data	Studies are descriptive instead of empirical
**Study Focus**	Pre-service teachers’ beliefs or attitudes toward ICT and various technologies	Pre-service teachers’ beliefs about anything else other than ICT or technologies
**Population and Sample**	Pre-service teachers of all disciplines in recognized universities/colleges or equivalent level	All other teachers are not pre-service teachers
**Language**	English	Non-English studies
**Time Period**	Studies published or unpublished in the public domain from 2000 to 2024 (after 21st century)	Studies outside these dates

### Study selection

The employment of a broad search with key terms generated a list of 1136 records. The search logs were documented during the literature search process. 310 duplicates were removed from the records; thus, 1056 studies were included in the first-stage screening which was title and abstract screening. In order to document the whole review process in one place, Microsoft Excel spreadsheets were used for the screening and later data extraction activities. A review of the titles and abstracts revealed that 673 studies were irrelevant; those studies, primarily, had no relation to ICT beliefs or perceptions, pre-service teachers, and teaching activities with technologies. The number of reports sought for retrieval was 383. Of these, 29 full-text articles were retrieved and obtained from the British Library document delivery service or by contacting authors via ResearchGate or email. This process offered an additional way to discover and identify any additional or further relevant studies that could be included in the review list. 35 reports were not retrieved in full-text which leads to that 348 studies were included in the second-stage screening, the full-text screening. By means of this assessment, 67 studies were excluded from the full-text screening process. As a result, 281 studies were included totally in the corpus for the final stage of the scoping review. S2 Table demonstrates the study selection process for this scoping review.

**Fig 1:**
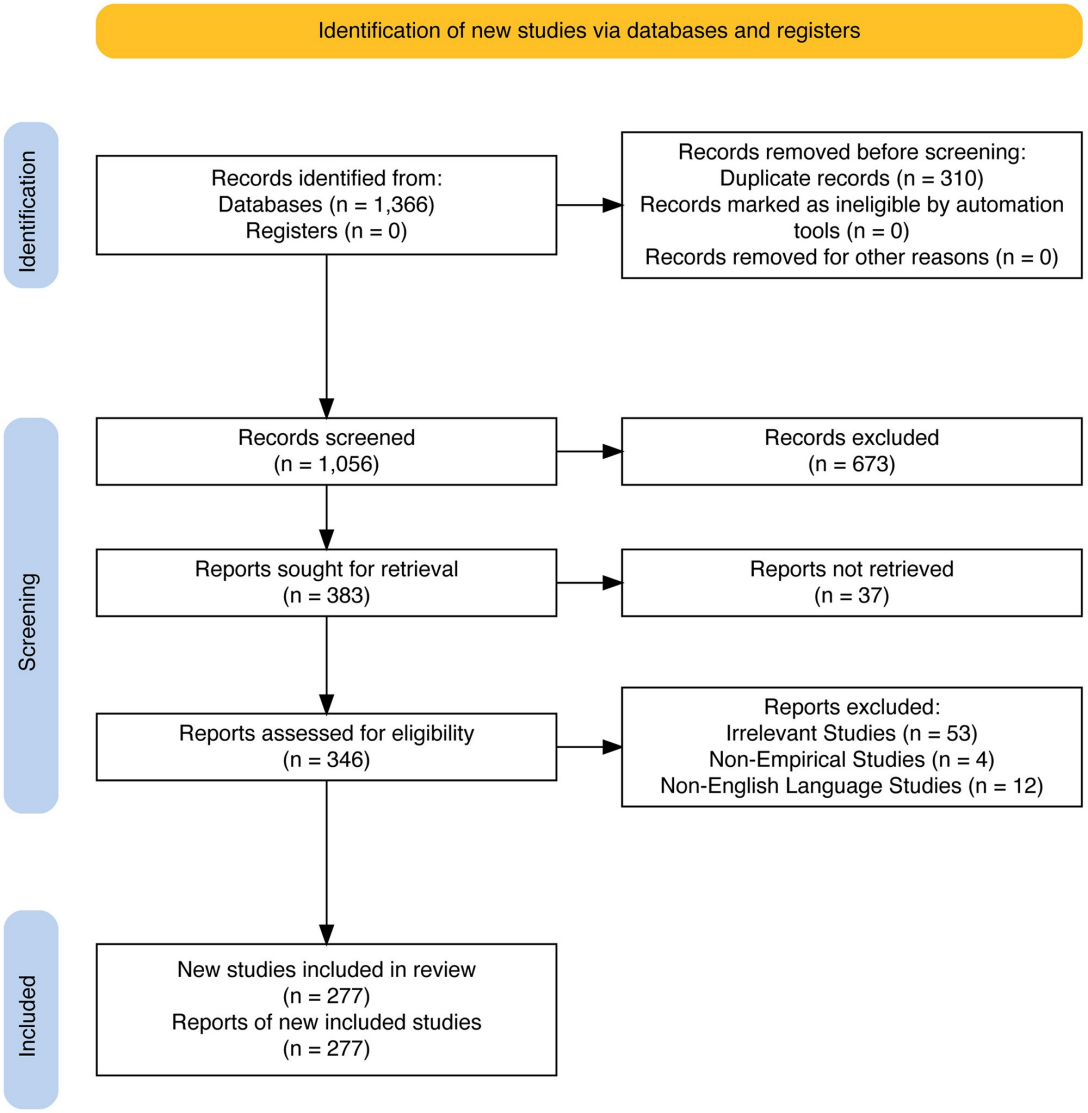
PRISMA flow diagram.

The process of study selection followed the inclusion and exclusion criteria and the PRISMA 2020 statement [8]. Fig 1 illustrates the overall process of study selection.

### Data charting

The data charting process in a scoping review is a critical step to structure and synthesize the vast amount of data collected from various sources. This process facilitates the understanding and interpretation of broad thematic patterns and trends within the scope of the review. To ensure replicability and transparency, the following detailed steps were undertaken: 1) Development of a Data Extraction Template: Before charting the data, a structured data extraction form was designed to ensure consistent and systematic collection of data across all studies; 2) Data Extraction: Each article selected for inclusion was read in detail, and relevant data were extracted and entered into the template. 3) Updating Data Extraction Template: The data extraction template was iteratively updated as needed when new relevant categories of data emerged during the charting process.

The data extraction form (see Table 3) was designed to provide a solid structure for data charting. The descriptive key elements and characteristics of the included studies were collected, such as general citation information (title, authors, year, and study types), research methods, types of ICT, subjects, settings, regions, training needs, and detailed information of the instruments used in the studies (the name, origin, and content of the surveys). Some of the categories were predefined by predictable elements, such as the use of different research methods, i.e., quantitative research, qualitative research, and mixed-method research.

**Table 3:** Data extraction form.

**ID**	
**Title**	
**Authors**	
**Year**	
**Study Types**	Journal
	Thesis
	Proceedings
	Manuscript
**Topic Categories**	
**Research Methods**	Mixed Methods
	Quantitative Research
	Qualitative Research
**Types of ICT**	General or Specific
**Subjects**	
**Settings**	Pre-school
	Primary School
	Secondary School
	Higher Education
**Demographic Information**	Yes
	No
**Surveys**	(Name, Origin, Content)
**Qualitative Methods**	Interview/Focus Group
	Observation
	Others
**Training Needs**	Yes
	No
**Memo**	

The selection of literature descriptors was guided by several criteria to ensure comprehensive coverage of the topic and relevance to the research questions: 1) Relevance to Research Questions: Descriptors were specifically chosen to reflect the key aspects of the research questions, such as types of ICT tools, attitudes towards ICT, and the educational context; 2) Consistency Across Studies: Descriptors that frequently appeared across multiple studies were included to maintain consistency and comparability of data. 3) Theoretical Framework: Theoretical frameworks discussed in the literature influenced the choice of descriptors to ensure that they were grounded in existing knowledge; 4) Feedback from Experts: Preliminary lists of descriptors were reviewed by experts in educational technology to ensure their appropriateness and comprehensiveness; 5) Pilot Testing: Initial descriptors were pilot tested on a subset of articles to assess their effectiveness in capturing relevant data before finalizing them for the full review. The extracted information, therefore, provides an overall perspective on the themes emerging from the selected studies relating to the research questions. Key information was extracted to S3 Table. The missing data were marked “unspecified” or “NA” in the data extraction process.

### Data synthesis

The final stage of this scoping review was to synthesize and report the findings based on the research questions. Having a plan for data extraction and focusing on the descriptive nature of the studies during the charting stage enabled a straightforward description of the literature by creating established categories and subcategories. The purpose of this final stage included narrative synthesis and interpretation of the findings.

## Results

### Overview

The initial search yielded 1366 studies; however, these were reduced to a total of 277 studies by using the inclusion and exclusion criteria to refine the selection of the included studies in the final synthesis. The included studies were generally from four different sources: journal articles, conference proceedings, theses, and other manuscripts. The charting revealed that seven different topic categories were identified from the included studies, and the majority of the studies focused on ICT beliefs in general (n = 173). Other types of topics with regard to ICT beliefs included teacher ICT training programs (n = 35), technology competence and literacy (n = 22), comparison of ICT beliefs (n = 12), relationship studies (n = 15), ICT beliefs modeling (n = 10), and the perceptions of ICT-based teaching and learning (n = 10). 60% of the reviewed studies employed quantitative methods, whereas 25% and 15% of the studies adopted mixed methods and qualitative methods, respectively. A brief summary of the frequency and percentage of literature descriptors of the included studies is provided in Table 4.

**Table 4:** Frequency and percentage of literature descriptors.

Variables and Factors	Frequency	Percentage
**Study Types**		
Journal	233	84.12%
Proceedings	14	5.05%
Thesis	29	10.47%
Manuscript	1	0.36%
**Topic Categories**		
ICT Beliefs	173	62%
Teacher ICT Training Program	35	13%
Technology Competence and Literacy	22	8%
ICT Beliefs Comparison	12	4%
Relationship Studies	15	5%
ICT Beliefs Modeling	10	4%
Perception of ICT Based Teaching & Learning	10	4%
**Research Methods**		
Mixed	69	25%
Quantitative	167	60%
Qualitative	41	15%
**Types of ICT**		
General	160	57.76%
Computer ^a^	47	16.97%
Internet	14	5.05%
Digital Storytelling	11	3.79%
Wiki/Blog	5	1.81%
Portable Devices ^b^	4	1.44%
Web 2.0	3	1.08%
Others ^c^	33	11.91%
**Subject of the Teaching**		
Unspecified	116	41.88%
Mixed	43	15.52%
Language	34	12.27%
Science	24	8.66%
Math	24	8.66%
Sports	7	2.53%
Computer	7	2.53%
Health	4	1.44%
Chemistry	3	1.08%
STEM	3	1.08%
Biology	2	0.72%
Social Studies	2	0.72%
Physics	2	0.72%
Art	2	0.72%
Geography	2	0.72%
Music	1	0.36%
Special Education	1	0.36%
**Settings**		
Pre-school	11	4%
Primary School	42	15%
Secondary School	15	5%
Mixed	42	15%
Unspecified	167	60%
**Regions of Study**		
Turkey	79	N/A ^e^
United States	67	N/A
China	14	N/A
Singapore	13	N/A
Australia	10	N/A
Indonesia	9	N/A
Malaysia	7	N/A
United Kingdom	5	N/A
Others ^d^	78	N/A
**Training Needs**		
Yes	94	34%
No	182	66%
**Demographic Information**		
Yes	160	58%
No	117	42%

^a^ Computer included: Computer Programming, Computer Program, Computer Games, Computer Generated Art Imagery, Computer Algebra Systems.

^b^ Portable Devices included: Tablets, iPad, PDA, and Handheld Technology.

^c^ Other types of ICT included: Science Lab, E-Picture Books, Online Discussion Board, e-Portfolios, Drawing, E-health, Social Media, Graphing Calculator, VBL (video-based case learning), Online Technologies, Dynamic Visualization, Audio-visual Technology, Slides, Interactive Whiteboard, WebQuests, Computing Technologies, Digital Text, Website, Visual Arts, New Media, Digital Library, Video Production Technology, Learning Management System, 3D Modeling, 3D Printing, Virtual Reality, Augmented Reality, Chatbots, and ChatGPT.

^d^ Other countries included: Belgium, Canada, Cyprus, Czech, Estonia, Finland, Germany, Ghana, Greece, India, Indonesia, Iran, Israel, Kazakhstan, Kuwait, Malta, Morocco, Netherland, New Zealand, Nigeria, Norway, Poland, Portugal, Romania, Rome, Russia, Saudi Arabia, Serbia, Slovenia, South Africa, South Korea, Spain, Sweden, Swiss, Tanzania, Thailand, Uganda, United Arab Emirates, and Yemen.

^e^ Not Applicable in percentage counting because some studies included multiple regions.

The majority of the ICT described in the studies was ICT in general (not specifying one particular type of ICT tool) (n = 160), followed by computers (n = 47), internet (n = 14), digital storytelling (n = 11), wiki/blog (n = 5), portable devices (n = 4), web 2.0 technologies (n = 3), and other types of technologies (n = 33). The teaching subject of the pre-service teachers involved was not specified or mentioned in some studies, whereas nearly 58% of the studies reported the teaching subject of the participants. Additionally, 60% of the studies did not provide the settings or the level of teaching of the pre-service teachers; however, in those studies that identified the settings, primary and secondary school settings made up one-fifth. Only eleven studies included in this review on empirical studies of pre-service teachers were set in pre-schools.

In addition, the included sources came from 47 different countries, which geographically reflected that Turkey (n = 79) and the United States (n = 67) showed a greater state of interest in pre-service ICT beliefs, followed by China (n = 14), Singapore (n = 13), Australia (n = 10), Indonesia (n = 9), Malaysia (n = 7), the United Kingdom (n = 5), and other countries (n = 78). Surprisingly, more than one-third of the studies mentioned training needs in the discussion, and 58% of the studies collected the participants’ demographic information. More information in terms of topic categories and research methods in the included studies is provided in the next section.

### Review question 1

The contribution to review question 1 comes in the form of extracted information from the included studies relating to the seven identified themes, namely, ICT beliefs in general, teacher ICT training program, technology competence and literacy, ICT belief comparison, relationship studies, and the perception of ICT-based teaching and learning. These seven themes represent the most discussed issues regarding the ICT beliefs of pre-service teachers. The most frequently noted theme was that of studies on the description of pre-service teachers’ ICT beliefs in general [e.g., 9–15], and the other most frequently noted topic was the measurement of pre-service teachers’ ICT beliefs in teacher ICT training programs [e.g., 16–18]. Topics on technology competence and literacy mainly focused on the confidence and self-efficacy of pre-service teachers with regard to digital literacy and competence [e.g., 19–25].

In addition, other topics like comparison studies were mainly focused on differences in the ICT beliefs of pre-service teachers from different regions or departments [26–29] and on comparisons between in-service teachers and pre-service teachers’ ICT beliefs [30]. The literature on the perceptions of ICT-based learning and teaching reflects pre-service teachers’ attitudes towards the use of ICT tools in a certain way [31–34].

### Review question 2

The design of the included empirical studies in this scoping review mostly relied on previous research and was therefore progressive or cumulative in nature. The two most commonly used kinds of instruments for quantitative data collection in the selected studies were the Technology Attitude Scale [e.g. 35–37] and the Computer Attitude Scale [e.g. 27, 38–40]. Other surveys in the selected studies adopted the Attitude towards Educational Technology Scale [26], Technology Perception Scale [18, 41–43], General Attitudes Towards Digital Technologies [14], Digital Technologies Survey [44], Technology Acceptance Scale [45], Attitude Scale for Digital Technology [46], Attitude Scale towards the ICT [47], Internet Attitude Scale [38, 48–51], Web 2.0 Attitude Scale [43], TPACK Confidence Scale [22, 52–54], or Attitude Scale towards Instructional Technologies [55].

Most studies adopted or modified the categories of the Technology Acceptance Model (TAM) into the instruments aforementioned; for example, Teo [56] extended the TAM model in their study by including perceived usefulness (PU), perceived ease of use (PEU), subjective norms (SNs), facilitating conditions (FCs), technological complexity (TC), and attitudes towards computer use (ATCU). The most commonly used original sources of the surveys adopted in the selected studies can be tracked from the citations of previous studies [57–61], and other common original sources of surveys can also be tracked [56, 62–65].

As for qualitative data collection methods, interviews or focus groups [e.g. 15, 35, 66–76], open-ended questions [23, 41, 77–80], observations [e.g. 53, 71, 78, 81], and written responses [e.g. 72, 82–87] were the top-four approaches. Other approaches, such as portfolios, lesson plans, digital stories, micro-teaching, discussion, blogging, metaphors, artifacts, drawing, comics, visual associated activity, journals, and document reviews, were also included in qualitative studies.

Direct questions in interviews and focus groups were mostly used and focused on the experience of the perception and use of the technology in teaching; for example, Al-Awidi and Alghazo [88] used four categories of experience to elicit pre-service teachers’ ICT beliefs: (1) mastery experience: ‘‘What experiences affected your choice and decision to integrate technology in your teaching?”; (2) vicarious experience: ‘‘Was there anyone who had an effect on your beliefs to integrate technology in your teaching?” and ‘‘How did those people affect you?”; (3) social persuasion: ‘‘How have people around you encouraged you to utilize technology?”; and (4) psychological and emotional states: ‘‘How do you feel when you integrate technology in your teaching?”.

## Discussion

### Review outcomes and recommendations

This scoping review employed a systematic approach to identify studies addressing pre-service teachers’ ICT beliefs. This approach was underpinned by the PRISMA statement [6] and the framework developed by Arksey and O’Malley [3]. By uncovering a considerable volume of studies, outlining the framework of the existing literature, and extracting the key data from a cataloged database of the literature in a comprehensive manner, the research revealed the crucial factors in terms of measuring pre-service teachers’ ICT beliefs, exploring unresolved issues within the current literature in terms of pre-service teachers’ beliefs regarding different technologies, and providing clear directions for the development of further educational research and practice.

Two research questions were answered by synthesizing the key data extracted from the selected studies in terms of the topic categories and methodologies in the literature on pre-service teachers’ ICT beliefs. These added rigor to the scoping review process and thus serve as strengths. This also contributes to the methodological aspect of conducting the scoping review systematically by providing and expatiating the approaches adopted in the research reports, which could help move the relevant research field forward. The predominance of quantitative studies highlights a methodological bias, underscoring the need for qualitative explorations to capture nuanced perspectives. Regional imbalances in study distribution suggest systemic barriers to ICT research in underrepresented areas. Future research should prioritize these regions to ensure a more equitable understanding of ICT beliefs globally and integrate qualitative approaches by providing deeper insights into contextual factors influencing ICT beliefs.

Seven themes were categorized in this scoping review, and the majority of studies focused on the perception of pre-service teachers regarding ICT in general or regarding one specific ICT tool. Very few studies focused on differences in ICT beliefs at a regional or national level or on the perception of multiple technologies. Even though a few studies considered pre-service teachers’ beliefs on trending technologies, such as portable devices, there has a few studies regarding the perception of innovative and cutting-edge technologies from pre-service teachers. Given the evidence that a large portion of the reviewed studies did not specify the subjects or levels of pre-service teachers for their future teaching, it is suggested that researchers should provide basic demographic information to a greater extent to provide future readers and researchers the opportunity to understand the background of the research settings. Potential ICT training needs should be discussed for future studies to develop relevant professional training programs for pre-service teachers in order to move forward and make a difference in this research field. It is also suggested that future research on pre-service teachers’ ICT beliefs could be based more on cutting-edge technologies, such as wearable devices, artificial intelligence tools, virtual reality, augmented reality, extended reality etc., by providing comprehensive demographic information and research backgrounds and contributing to the analysis of corresponding training needs for these ICT tools.

The significance of understanding pre-service teachers’ beliefs about ICT lies in its potential to influence their readiness to integrate technology into classrooms effectively. These beliefs influence how future teachers will use ICT in their classrooms, impacting the quality of education and the success of technological integration. The integration of ICT in teaching is essential for modern education, offering enhanced learning outcomes, greater flexibility, and innovative teaching methods. On the one hand, pre-service teachers need continue to embrace and overcome the barriers to fully harness the potential of ICT in education. On the other hand, the research conducted in the future need to be diversified and be able to catch the trend of the technology development. The integration of ICT in teaching still faces challenges, such as lack of resources, time, access, and technical support. Future studies could focus on the training needs for the process of pedagogical evolution, where teachers could trial new ICT tools and develop new strategies for ICT-supported learning.

### Challenges and limitations

In this study, a scoping review was used as a rapid method for mapping and synthesizing the existing literature in this particular topic area and for identifying knowledge gaps for future research; however, the researcher still encountered challenges in terms of the methodology itself and the process of conducting the review. Scoping reviews are considered to be a rapid review method to synthesize knowledge in a systematic but simplified manner [89]; however, they are not a rapid process [90]. After developing a search strategy, obtaining a comprehensive entry of the existing literature, and selecting studies using inclusion and exclusion criteria, the data extraction and charting processes were demanding. Thus, in this scoping review, through prioritizing the purpose of finding the knowledge gap for further research, an optimized balance was achieved.

In order to generate and capture a sufficient breadth of coverage and substantial range of the literature relating to the topic of interest, this scoping review included non-peer-reviewed articles or manuscript in English only, which raises the possibility of excluding relevant studies in other languages in this review. Scoping reviews share part of the process of systematic review methodology; however, they are not intended to assess the quality of the scoped literature. While the lack of certain literature was identified, since the quality of the selected studies could not be assessed, there may be further unidentified gaps [91]. Furthermore, with the lack of quality control, compared to systematic review, there can be limited understanding and acceptance of labeling the review category based on numerous types of rapid review methods. For instance, by evaluating the purpose, nature, and process of the review, this review could either be labeled as a mapping review [91] or a scoping review [3]. Therefore, after the completion of this scoping review, it is imperative to reach a consensus and advance the current guidelines for scoping reviews and mapping reviews, which could improve the transparency and acceptance of rapid review methods.

The data extraction process involves some judgment rather than being a purely mechanical summary. For example, some authors did not refer to “demographic information was collected”, but they nevertheless listed the detailed information of the participants, such as the subjects, teaching grade, age, and gender. Although this was a major advantage in the process of summarizing the topics and methodologies in the included studies, other attributions, such as the sample size of the data, strategies of data analysis, and findings, could also be considered and cataloged more extensively in the data extraction and charting process based on the results and nature of the empirical research.

Since the completeness of this scoping review was determined by time constraints, it still needs to be updated in the future to cover the latest literatures. Technology in education is a rapidly evolving field, with continual advancements that could potentially alter the landscape of pre-service teacher education significantly. The inclusion of studies up to July 2024 in this review was necessary to manage the scope of the review effectively and to provide a definitive conclusion point for the initial phase of analysis. It is acknowledged that this time stamp limits the inclusion of subsequent developments after the time period of the search date (19 July 2024), which may have introduced new perspectives or technologies not considered in this review. However, it is worth mentioning that the task of updating a comprehensive scoping review is large, and it cannot be undertaken without “the perpetual availability of ongoing resources and personnel” [90]. However, this limitation highlights the need for future research to explore historical and non-English contributions to provide a holistic perspective. These limitations are not detrimental to the report presented in this review but do indicate further considerations for conducting the review, interpreting the findings and planning research in the future.

## Conclusions

This scoping review produced a comprehensive literature synthesis from a large volume of literature pertaining to pre-service teachers’ ICT beliefs. Through the comprehensive review, the research has mapped the prevailing perspectives, methodologies, and findings across diverse educational settings and geographic contexts. The analysis has confirmed that while there is a general acknowledgment of the importance of ICT in teacher education, there remains a significant diversity in beliefs about its utility and implementation. These beliefs are influenced by a variety of factors including individual confidence, institutional support, and exposure to technology during teacher training.

The scoping review also identified a number of research gaps in the literature that need to be addressed in future research, and it enables future researchers to carry out more in-depth reviews, such as systematic reviews, based on the current literature mapping. There is limited research exploring the longitudinal impact of pre-service teachers’ beliefs about ICT on their actual teaching practices once they enter the workforce. Few studies address the impact of cultural and regional differences on ICT beliefs among pre-service teachers. In addition, there is a need for more empirical research focusing on the effectiveness of specific interventions designed to alter or enhance pre-service teachers’ beliefs and competencies in ICT.

This study not only serves as a foundational resource for academics and practitioners interested in the nexus of ICT and teacher education but also sets the stage for future research endeavors. The resulting repository in this review could provide preliminary information and may be useful to researchers interested in the topic of pre-service teachers’ ICT beliefs. Future reviews should extend the review period to include the latest research. Regular updates to the scoping review could ensure that the synthesis remains current with technological advancements and shifts in educational theory and practice. Future reviews should also consider including a section on emerging technologies and their potential impact on education by scanning the horizon for new innovations in educational technology and predicting how these might influence future research directions. By addressing these elements in future studies, researchers can significantly enhance the quality and relevance of their reviews, by providing valuable, timely insights that reflect the latest developments in the field of education technology.

In conclusion, this scoping review has laid a substantial groundwork for understanding the current landscape of pre-service teachers’ ICT beliefs. It highlights the importance of these beliefs in shaping future educational practices and underscores the critical need for further research that can guide policy and practice in teacher education. Through such efforts, the educational sector can better harness the potential of ICT to foster more engaging and effective teaching environments.

## Supporting information

S1 Table PRISMA checklist. (PDF)

S2 Table Study selection. (PDF)

S3 Table Overview of included studies. (PDF)
