# Supplementary material for: Integrating ICT in education: A scoping review of pre-service teachers’ ICT beliefs
Source: PLoS One. 2025 Feb 7;20(2):e0317591. doi: 10.1371/journal.pone.0317591 (PMC11805408; doi:10.1371/journal.pone.0317591)
Supplement: S2 Table — (PDF) [file pone.0317591.s002.pdf]

Integrating ICT in education: A scoping review of pre-service teachers' ICT beliefs

S2 Table. Study Selection

Some Factors in the Development of Self-Efficacy Beliefs for Computer Use among Teacher Education Students	Albion, Peter R.	2001	Included	
Impact of a verbal persuasion treatment on teacher education students' attitudes and self-efficacy for computer technology	DuBay, Tracy Lynn; Gredler, Margaret	2001	Included	
An investigation of traditional and constructivist models for Internet training and attitudes of pre -service teachers	Zhang, Chenfeng; Koontz, Franklin	2001	Included	
Effects of inservice training on the technology practices and attitudes of physical education teachers	Berkowitz, R. J.; Tannehill, D. L.	2001	Excluded	Irrelavant Topic
Great Expectations?:: Brazilian Managerial Perceptions on Prospective European High-Technology Joint Ventures	De Mattos, Claudio; Neto, Cesar Gon??alves; Sanderson, Stuart	2001	Excluded	Irrelavant Topic
Preservice teachers' perceptions regarding the use of computers in supervising an elementary physical education field experience	Jones, D. L.; Garrahy, D.	2001	Excluded	Irrelavant Topic
Influences on perceptions of student violence: Is it more about the teacher than the student?	Johll, M. P.; Brant, C. R.; Najpaver, C. M.; Hoffman, P. M.; Dwyer, M. D.	2001	Excluded	Irrelavant Topic
Student Teachers' Perception and Practice of the Teachers' Role when Teaching with Computers	Wang, Yu-Mei	2001	Excluded	Irrelavant Topic
Understanding student teachers' perceptions on the teaching and learning of english as a foreign language through their analysis of computer generated materials	Afonso, Carlos	2001	Excluded	Full Text Not Available
Student teachers eliciting mentors?€? practical knowledge and comparing it to their own beliefs	Zanting, A.; Verloop, N.; Vermunt, J. D.	2001	Excluded	Irrelavant Topic
Student teachers' perceptions of the Internet: Motivation, influences, and use	Almeida, C.; Morais, C.; Miranda, L.; Viseu, F.; Martinho, H.	2002	Included	
Comparison of the perceptions of university faculty and pre -service students' technology skills and integration of technology in selected elementary education courses	Best, Linda M.; Tidwell, Monte G.	2002	Included	
Using electronic bulletin boards and journals to enhance pre-service	Burkett, Ruth S.; Barron, Ann E.	2002	Included	

teachers self-efficacy and attitudes toward the use of computers				
Prospective high school mathematics teachers?€? attitudes toward integrating computers in their future teaching	Hazzan, O.	2002	Included	
Integrating technology into preservice teacher education programs: A study of preparedness, attitudes, and self -efficacy	Hornung, Claire Smith; Moe, Alden	2002	Included	
Individual differences in nurse and teacher training students' attitudes toward and use of information technology	Wishart, J.; Ward, R.	2002	Included	
A description and comparison of RN -BSN nursing students' perceptions of student -teacher relationships in traditional and Internet distance education nursing courses	Al-Saleh, Mary Margaret; Allen, Lois Ryan	2002	Excluded	Irrelavant Topic
Attitudes Toward Integration as Perceived by Preservice Teachers Enrolled in an Integrated Mathematics, Science, and Technology Teacher Education Program	Berlin, Donna F.; White, Arthur L.	2002	Excluded	Irrelavant Topic
Integrating learning technology into classrooms: The importance of teachers' perceptions	Chris, Cope; Peter, Ward	2002	Excluded	Irrelavant Topic
Student teachers' beliefs about science, design and technology: influences on planning for activities in the primary classroom	Davies, Daniel John	2002	Excluded	Full Text Not Available
?€?Teaching is not at all what it's advertised to be!?€?: A narrative approach to examining student teachers' knowledge and beliefs about teaching	Pittard, M. Michele; Phillion, JoAnn; Finders, Margaret J.	2002	Excluded	Irrelavant Topic
Students teacher's perceptions of instructional technology: Developing materials based on a constructivist approach	??ahin, T. Y.	2003	Excluded	Irrelavant Topic
Impact of socially desirable instrumentality and socially desirable expressiveness on female pre -service teachers' computer attitudes	Huang, Chih-Hsun; McNeil, Sara	2003	Included	
Pre-service geography teachers' self perceptions of readiness for technology implementation	Lee, Robert Earl; McLean, Robert S.	2003	Included	
A study of pre -service teachers' attitudes concerning the use of technology in the classroom	Sheeran, Linda Rae; Jordan, Patricia Lamphere	2003	Included	
Patient attitudes to the internet and analysis of the potential role of a dedicated colorectal website - A prospective study	Birchley, D.; Pullan, R.; DeFriend, D.	2003	Excluded	Irrelavant Topic

To Change Perceptions of Technology Programs	Hansen, John W.	2003	Excluded	Irrelavant Topic
It Is a Gender Issue! Changes in Attitude towards Science in a Technology Based K-8 Pre-Service Preparation Science Classroom	Norby, Rena Faye	2003	Excluded	Irrelavant Topic
Student Teachers' Perceptions of Instructional Technology: Developing Materials Based on a Constructivist Approach	Sahin, Tugba Yanpar	2003	Excluded	Irrelavant Topic
Core French Teachers and Technology: Classroom Application and Belief Systems	Turnbull, Miles; Lawrence, Geoff	2003	Excluded	Irrelavant Topic
The Effects of Case-Based Learning on Early Childhood Pre-Service Teachers' Beliefs about the Pedagogical Uses of ICT	Angeli, Charoula	2004	Included	
The Impact of Training in Technology Assisted Instruction on Skills and Attitudes of Pre-Service Teachers	Benson, Linda F.; Farnsworth, Briant J.; Bahr, Damon L.; Lewis, Valerie K.; Shaha, Steven H.	2004	Included	
The study on the pre-service teachers' attitudes and belief on the integration of infonnation technology in teaching	Chang, Y. T.; Lai, Y. Z.; Hwang, B. C.; Hsu, Y. S.; Cantoni, L.; McLoughlin, C.	2004	Included	
Technology disposition of teacher education students: Beliefs, attitudes, self-concepts, and competence	Jung, Eun Joo; Rhodes, Dent M.	2004	Included	
The Effect of Electronic Scaffolding for Technology Integration on Perceived Task Effort and Confidence of Primary Student Teachers	Angeli, Charoula; Valanides, Nicos	2004	Excluded	Irrelavant Topic
Teaching and learning with technology: Kuwaiti mathematics pre - service teachers' competencies and attitudes	Mohammad, Anwar H.; Marshall, J. Daniel	2004	Included	
Erratum: Patient attitudes to the internet and analysis of the potential role of a dedicated colorectal website - A prospective study (Annals of the Royal College of Surgeons of England (2003) 85 (398-401))	Birchley, D.; Pullan, R.; DeFriend, D.	2004	Excluded	Irrelavant Topic
Pedagogical Strategies to Increase Pre-Service Teachers' Confidence in Computer Learning	Chen, Li-Ling	2004	Excluded	Irrelavant Topic
An Exploratory Study of the Effect a Self-Regulated Learning Environment Has on Pre-Service Primary Teachers' Perceptions of Teaching Science and Technology	Corrigan, Gerry; Taylor, Neil	2004	Excluded	Irrelavant Topic
"Is It Friday Yet?" (Perceptions of First-Year Music Teachers)	Fredrickson, William E.; Neill, Sheri	2004	Excluded	Irrelavant Topic
Will "The Way They Teach" Be "The Way They Have Learned"? Pre-Service Teachers' Beliefs Concerning Computer Embedding in Math	Gorev, D.; Gurevich, I.; Barabash, M.	2004	Excluded	Irrelavant Topic

Teaching				
Physical education teacher educators' perceptions of pre -service teachers' technology competencies and skills	Lindauer, Jeffrey R.; Wittenburg, David	2004	Excluded	Irrelavant Topic
Turkish Pre-Service Science Teachers' Understanding of Science and Their Confidence in Teaching It	Tekkaya, Ceren; Cakiroglu, Jale; Ozkan, Ozlem	2004	Excluded	Irrelavant Topic
Staying connected: Student teachers?€? perceptions of computer?€?mediated discussions	Assaf, L. C.	2005	Included	
Speculations on the Insights and Perceptions of Professor William E. Warner Regarding the Status of Technology Education and Its Future	Buffer, James J.	2005	Excluded	Irrelavant Topic
Teacher Pedagogical Beliefs: The Final Frontier in Our Quest for Technology Integration?	Ertmer, Peggy A.	2005	Excluded	Irrelavant Topic
Teachers' and Pre-Service Teachers' Gendered Beliefs: Students and Computers	Forgasz, Helen	2005	Excluded	Irrelavant Topic
Gender and Attitudes of Pre-Service Second Language Teachers Towards Technology in Teacher Preparation Programs	Guo, R. X.	2005	Excluded	Full Text Not Available
Higher order applications of technology: Perception of WebQuests among pre-service teachers	Ho, H. J.; Choi, C. C.; L. EArning eDvantage; Litespeed Education PTE LTD; Microsoft,; Smart Technologies; promethean - enrich, enligthen inspire	2005	Included	
Climbing the Stairs: Pre-Service Social Studies Teachers' Perceptions of Technology Integration	Lipscomb, George B.; Doppen, Frans H.	2005	Excluded	Irrelavant Topic
Pre-Service Art Teacher Negative Attitudes and Perceptions of Computer-Generated Art Imagery: Recommendations for Pre-Service Art Education Programs	Lu, Li-Fen Lilly	2005	Included	
Experienced and Inexperienced Internet Users among Pre-Service Teachers: Their Use and Attitudes toward the Internet	Luan, Wong Su; Fung, Ng Siew; Nawawi, Mokhtar; Hong, Tang Sai	2005	Included	
Student teachers' attitudes to and use of computers to teach mathematics in the primary classroom	Mc Alister, M.; Dunn, J.; Quinn, L.	2005	Included	
Are We Assuming Too Much? Exploring Students' Perceptions of Their Computer Competence	Messineo, Melinda; DeOllos, Ione Y.	2005	Excluded	Irrelavant Topic
Pre -service teachers' beliefs and experiences with computing	Porfilio, Bradley J.; Bromley, Hank	2005	Included	

technology and male centered computing culture				
The effect of handheld technology use in pre -service social studies education on the attitudes of future teachers toward technology integration in social studies	van 't Hooft, Mark A.; Crowe, Alicia R.	2005	Included	
Malaysian female pre-service teachers online: Exploring their Internet use and attitudes	Wong, S. L.; Ng, S. F.; Tang, S. H.; L. EArning eDvantage; Litespeed Education PTE LTD; Microsoft,; Smart Technologies; promethean - enrich, enligthen inspire	2005	Included	
Developing a Technology Attitude Scale for Pre-Service Chemistry Teachers	Yavuz, Soner	2005	Excluded	Irrelavant Topic
The Teacher Research Update Experience: Perceptions of Practicing Science, Mathematics, and Technology Teachers	Barnes, Marianne B.; Hodge, Emilia M.; Parker, Melinda; Koroly, Mary Jo	2006	Excluded	Irrelavant Topic
The Impact of Modeling Technology Integration on Pre-Service Teachers' Technology Confidence	Adamy, Peter; Boulmetis, John	2006	Included	
Turkish Prospective Teachers' Perceptions regarding the Use of Computer Games with Educational Features	Can, Gulfidan; Cagiltay, Kursat	2006	Included	
Perceptions of Beginning Teacher Education Students regarding Educational Technology	Friedman, Adam; Kajder, Sara	2006	Included	
Analyzing Pre-School Student Teachers' and Their Cooperating Teachers' Attitudes towards the Use of Educational Technology	Kabadayi, Abdulkadir	2006	Included	
Using a Student-Centred Learning Approach to Teach a Discrete Information Technology Course: The Effects on Malaysian Pre-Service Teachers' Attitudes toward Information Technology	Luan, Wong Su; Bakar, Kamariah Abu; Hong, Tang Sai	2006	Included	
Transferring and transforming technology education: A study of norwegian teachers' perceptions of ideas from design & technology	Bungum, B.	2006	Excluded	Irrelavant Topic
A prospective evaluation of the attitudes of patients, physicians and nurses using a computer-assisted quality of life instrument (LCSS-QL) in a multicenter clinical trial in non-small cell lung cancer (NSCLC)	Gralla, R. J.; Hollen, P. J.; Leighl, N.; Meharchand, J. M.; Krieger, H.; Solow, H.	2006	Excluded	Irrelavant Topic
Chapter 6: The effects of professional development on technological competency and the attitudes urban physical education teachers have toward using technology	Ince, M. L.; Goodway, J. D.; Ward, P.; Lee, M. A.	2006	Excluded	Irrelavant Topic

Relationship between Pre-Service and Practising Teachers' Confidence and Beliefs about Using ICT	Jamieson-Proctor, Romina; Finger, Glenn	2006	Included	
What we are really doing with ICT in physical education: a national audit of equipment, use, teacher attitudes, support, and training	Thomas, A.; Stratton, G.	2006	Excluded	Irrelavant Topic
Impact of STS issue oriented instruction on pre-service elementary teachers' views and perceptions of Science, Technology, and Society	Amirshokoohi, Aidin; Akerson, Valarie L.	2007	Excluded	Irrelavant Topic
The influence of a digital storytelling experience on pre-service teacher education students' attitudes and intentions	Gakhar, Sonia; Thompson, Ann; Schmidt, Denise; Hegland, Susan	2007	Included	
Factors Influencing Pre-Service Science Teachers' Perception of Computer Self-Efficacy	Hakverdi, Meral; Gucum, Berna; Korkmaz, Hunkar	2007	Included	
Prospective Mathematics Teachers' Attitudes Towards Learning Mathematics with Technology	Ipek, A. Sabri; Berigel, Muhammed; Albayrak, Mustafa	2007	Included	
Closing the gap: Pre-service teachers' perceptions of an ICT based, student centred learning curriculum	Lee, C. B.; Teo, T.; Chai, C. S.; Choy, D.; Tan, A.; Seah, J.; Blackboard,; E. M. C. Computer Systems; Echo,	2007	Included	
Attitudes of pre-service elementary teachers towards graphing calculator technologies in learning and teaching science	Lyublinskaya, I.; Zhou, G.	2007	Included	
Gender Differences in Attitudes towards Information Technology among Malaysian Student Teachers: A Case Study at Universiti Putra Malaysia	Su Luan, Wong; Atan, Hanafi	2007	Included	
That just shows I can't do it: Goal orientation and attitudes concerning help amongst pre service teachers	Dickhauser, O.; Butler, R.; Tonjes, B.	2007	Excluded	Irrelavant Topic
"It's Not My Job": K-12 Teacher Attitudes toward Students' Heritage Language Maintenance. Bilingual Research Journal, 30, 453-477. Jin Sook Lee and Eva Oxelson, 2006	Michael-Luna, Sara	2007	Excluded	Irrelavant Topic
New literacies with an attitude: Transformative teacher education through digital video learning tools	Miller, S. M.; Borowicz, S.	2007	Excluded	Irrelavant Topic
The perception of special and general education teachers of inclusion of students with emotional and behavioral disorders (EBD) as it relates to ?€?collaboration?€? and ?€?student-teacher?€? relationships	Robbins-Etlen, Carol A.; Hickman, Peggy	2007	Excluded	Irrelavant Topic

Attitudes toward information technology of teachers of the gifted - Implications for gifted education	Shaunessy, E.	2007	Excluded	Irrelavant Topic
An Information and Communications Technology (ICT)-Enabled Method for Collecting and Collating Information about Pre-Service Teachers' Pedagogical Beliefs regarding the Integration of ICT	Vallance, Michael	2007	Excluded	Irrelavant Topic
Effectiveness of Quantitative Skills, Qualitative Skills, and Gender in Determining Computer Skills and Attitudes: A Causal Analysis	Varank, Ilhan	2007	Excluded	Irrelavant Topic
The Impact of Internet Virtual Physics Laboratory Instruction on the Achievement in Physics, Science Process Skills and Computer Attitudes of 10th-Grade Students	Yang, Kun-Yuan; Heh, Jia-Sheng	2007	Excluded	Irrelavant Topic
Faculty perceptions of technology integration in the teacher education curriculum: A survey of two Ghanaian universities	Yidana, Issifu; Turner, Sandra V.	2007	Excluded	Irrelavant Topic
Transforming teacher education to support multicultural technology pedagogy: An assessment of preservice teachers' beliefs about multiculturalism and diversity	Bowser, Audrey Denise; Davis, Nicola; McShay, James	2008	Excluded	Irrelavant Topic
Beyond Motivation: ESL/EFL Teachers' Perceptions of the Role of Computers	Kim, Hoe Kyeung	2008	Included	
Learner to Teacher: EFL Student Teachers' Perceptions on Internet-Assisted Language Learning and Teaching	Kuo, Ming-Mu	2008	Included	
A Study of Pre-Service Teachers' Attitudes about Computers and Mathematics Teaching: The Impact of Web-Based Instruction	Lin, Cheng-Yao	2008	Included	
Beliefs about Using Technology in the Mathematics Classroom: Interviews with Pre-Service Elementary Teachers	Lin, Cheng-Yao	2008	Included	
Gender differences in the usage and attitudes toward the internet among student teachers in a public Malaysian university	Luan, W. S.; Fung, N. S.; Atan, H.	2008	Included	
A Longitudinal Assessment of Teacher Education Students' Confidence toward Using Technology	Milman, Natalie B.; Molebash, Philip E.	2008	Included	
The Evaluation of the Student Teachers' Attitudes toward Internet and Democracy	Oral, Behcet	2008	Included	
Learners' Attitudes to Wiki Technology in Problem Based, Blended	Robertson, Ian	2008	Included	

Learning for Vocational Teacher Education				
Efficacy of a planned behavior model: Beliefs that contribute to computer usage intentions of student teachers and experienced teachers	Smarkola, Claudia	2008	Included	
Pre-Service Teachers' Attitudes towards Computer Use: A Singapore Survey	Teo, Timothy	2008	Included	
Pre-service physical education teachers' attitudes toward, and use of, handheld technology	Wegis, Heidi M.	2008	Included	
User?€?s Gestural Exploration of Different Virtual Agents?€? Expressive Profiles (Short Paper)	Courgeon, Matthieu; Martin, Jean-Claude; Jacquemin, Christian	2008	Excluded	Irrelavant Topic
Spanish Teachers' Beliefs and Practices on Computers in the Classroom	Cummings, Anne	2008	Excluded	Irrelavant Topic
Using a voting system in conjunction with interactive whiteboard technology to enhance learning in the English language classroom	Cutrim, Euline Schmid	2008	Excluded	Irrelavant Topic
The keys for ICT integration in K-12 education: Teachers' perceptions and usage	Goktas, Y.; Yildirim, Z.; Yildirim, S.	2008	Excluded	Irrelavant Topic
The ?€?I?€?m feeling lucky syndrome?€?: teacher-candidates?€? knowledge of web searching strategies	Laverty, Corinne; Reed, Brenda; Lee, Elizabeth	2008	Excluded	Irrelavant Topic
Computer-Based Business Simulation Games as Tools for Learning: A Comparative Study of Student and Teacher Perceptions	Lindh, J.; Hrastinski, S.; Bruhn, C.; Mozgira, L.; Connolly, T.; Stansfield, M.	2008	Excluded	Irrelavant Topic
Changes in Learning Process Caused by the Implementation of ICT in Education in Estonian In-Service and Pre-Service Teachers Perceptions	Luik, Piret; Kukemelk, Hasso	2008	Excluded	Irrelavant Topic
Collaborative learning and ethics in online courses: In search of instructional techniques that promote ethical behavior	Moore, Margaret J	2008	Excluded	Irrelavant Topic
Teacher competences and pupil achievement in pre-school and school	Nordenbo, Sven Erik; Larsen, Michael S??gaard; Tiftik??i, Neriman; Wendt, Rikke Eline; ??stergaard, Susan	2008	Excluded	Irrelavant Topic
Applications and Exploration of ICT in Teaching History and Foreign Languages: Students and Teachers Attitudes, Perceptions and Evaluation	Pamouktsoglou, A.; Antonopoulos, J.; Kalouri, O.; Lytras, M. D.; Carroll, J. M.; Damiani, E.; Avison, D.; Vossen, G.; OrdonezDePablos, P.	2008	Excluded	Irrelavant Topic

Waldorf education and the neurodevelopment of intelligence: An integrative review	Prouty, Steven Edward	2008	Excluded	Irrelavant Topic
Preschool Teacher Perceptions of Assistive Technology and Professional Development Responses	Stoner, Julia B.; Parette, Howard P.; Watts, Emily H.; Wojcik, Brian W.; Fogal, Tina	2008	Excluded	Irrelavant Topic
Understanding Pre-Service Teachers' Computer Attitudes: Applying and Extending the Technology Acceptance Model	Teo, T.; Lee, C. B.; Chai, C. S.	2008	Excluded	Irrelavant Topic
Beliefs about Teaching and Uses of Technology among Pre-Service Teachers	Teo, Timothy; Chai, Ching Sing; Hung, David; Lee, Chwee Beng	2008	Excluded	Irrelavant Topic
A Survey on ICT Usage and the Perceptions of Social Studies Teachers in Turkey	Yasemin, Gulbahar; Ismail, Guven	2008	Excluded	Irrelavant Topic
ICT in Teacher Education: Examining Needs, Expectations and Attitudes	Zhang, Zuochen; Martinovic, Dragana	2008	Excluded	Irrelavant Topic
Trickle down Mathematics: Adult Pre-Service Elementary Teachers Gain Confidence in Mathematics--Enough to Pass It Along?	Ashun, Mary Apea; Reinink, John	2009	Excluded	Irrelavant Topic
ICTs for higher education	Balasubramanian, K; Clarke-Okah, Willie; Daniel, John; Ferreira, Frances; Kanwar, Asha; Kwan, Angela; Lesperance, John; Mallet, Joshua; Umar, Abdurrahman; West, Paul	2009	Excluded	Irrelavant Topic
Exploring Teachers' Beliefs About Teaching Knowledge: Where Does It Come From? Does It Change?	Buehl, Michelle M.; Fives, Helenrose	2009	Excluded	Irrelavant Topic
A STUDY ON SCIENCE TEACHERS' ATTITUDES TOWARD INFORMATION AND COMMUNICATION TECHNOLOGIES IN EDUCATION	Cavas, B.; Cavas, P.; Karaoglan, B.; Kisla, T.	2009	Excluded	Irrelavant Topic
Pre-Service Elementary Teachers' Beliefs about Use of the Internet in the Social Studies Classroom	Acikalin, Mehmet	2009	Included	
Ninth Graders' Learning Interests, Life Experiences and Attitudes Towards Science & Technology	Chang, Shu-Nu; Yeung, Yau-Yuen; Cheng, May Hung	2009	Excluded	Irrelavant Topic
Examining Perceptions of Systematic Integration of Instructional Technology in a Teacher Education Program	Allsopp, David H.; McHatton, Patricia Alvarez; Cranston-Gingras, Ann	2009	Included	
The pre-service teachers' competency perceptions regarding technology planning	Birinci, G.; Kilicer, K.; Uzunboylu, H.; Cavus, N.	2009	Included	

Assessing Pre-Service Teacher Attitudes and Skills with the Technology Integration Confidence Scale	Browne, Jeremy	2009	Included	
Primary student-teachers' perceptions of the role of digital literacy in their lives	Burnett, Catherine M.	2009	Included	
Singaporean and Taiwanese pre-service teachers' beliefs and their attitude towards ICT use: A comparative study	Chai, C. S.; Hong, H. Y.; Teo, T.	2009	Included	
"Even if No-One Looked at It, It Was Important for My Own Development": Pre-Service Teacher Perceptions of Professional Portfolios	Chitpin, Stephanie; Simon, Marielle	2009	Excluded	Irrelavant Topic
Investigating prospective computer teachers' perceptions on e-learning	Cobanoglu, I.; Ates, A.; Ilic, U.; Yilmaz, E.; Uzunboylu, H.; Cavus, N.	2009	Included	
Influence of the Familiarization with "Scratch" on Future Teachers' Opinions and Attitudes about Programming and ICT in Education	Fesakis, G.; Serafeim, K.; Acm,	2009	Included	
A programmatic profile of the uses, skills, and beliefs of preservice teacher education students and their instructors regarding online technologies	Freidhoff, Joseph R.; Dickson, W. Patrick	2009	Included	
Turkish Pre-Service Science and Mathematics Teachers' Computer Related Self-Efficacies, Attitudes, and the Relationship between These Variables	Pamuk, Savas; Peker, Deniz	2009	Included	
Personal Attitudes and Technology: Implications for Preservice Teacher Reflective Practice	Shoffner, Melanie	2009	Included	
Examining the Relationship between Student Teachers' Self-Efficacy Beliefs and Their Intended Uses of Technology for Teaching: A Structural Equation Modelling Approach	Teo, Timothy	2009	Included	
The Impact of Subjective Norm and Facilitating Conditions on Pre-Service Teachers' Attitude toward Computer Use: A Structural Equation Modeling of an Extended Technology Acceptance Model	Teo, Timothy	2009	Included	
The attitudes of prospective teachers' on trusting internet resources	Tezer, Murat; Bicen, H??seyin	2009	Included	
Determination of attitudes of students teachers towards the utilization of technology: creating a technology tree	Yucel, A. S.; Kocak, C.; Uzunboylu, H.; Cavus, N.	2009	Included	

Determining of student teachers self-confidence using technology in instruction	Erdemir, N.; Bakirci, H.; Eyduran, E.	2009	Excluded	Irrelavant Topic
A course on gender equity in education: Does it affect gender role attitudes of preservice teachers?	Erden, F. T.	2009	Excluded	Irrelavant Topic
CRP-Education	Findlay, Judith Blair	2009	Excluded	Irrelavant Topic
Investigating Technologies in Teacher Education: Does PowerPoint Enhance Retention or Influence Attitudes?	Giles, Rebecca M.; Baggett, Paige V.	2009	Excluded	Irrelavant Topic
Assistive Technology Approaches for Large-Scale Assessment: Perceptions of Teachers of Students with Visual Impairments	Johnstone, C.; Thurlow, M.; Altman, J.; Timmons, J.; Kato, K.	2009	Excluded	Irrelavant Topic
Changes in Attitudes Towards Science-Technology-Society of Pre-service Science Teachers	Kaya, O.; Yager, R.; Dogan, A.	2009	Excluded	Irrelavant Topic
An analysis of turkish prospective teachers' perceptions about technology in education	Koksal, M. S.; Yaman, S.	2009	Excluded	Irrelavant Topic
Technology Teachers' Beliefs About Biotechnology and Its Instruction in South Korea	Kwon, Hyuksoo; Chang, Mido	2009	Excluded	Irrelavant Topic
The Perception of African Teachers on Strategy and Technology in Teaching and Learning Islamic Education	Lubis, M. A.; Yusoff, Nmrn; Hamzah, M. I.; Wekke, I. S.; Perlovsky, L.; Dionysiou, D. D.; Zadeh, L. A.; Kostic, M. M.; GonzalezConcepcion, C.; Jaberg, H.	2009	Excluded	Irrelavant Topic
?€?Intellectual challenge is as necessary as breathing?€? Laurence Wright Interviewed by Brian Pearce	Pearce, Brian	2009	Excluded	Irrelavant Topic
Perceptions That May Affect Teachers' Intention to Use Technology in Secondary Mathematics Classes	Pierce, Robyn; Ball, Lynda	2009	Excluded	Irrelavant Topic
Pre-Service Music Teachers' Perceptions of Social Justice and/or Social Consciousness as it Relates to Music Education	Riley, P. E.	2009	Excluded	Irrelavant Topic
Pre-Service Music Teachers' Perceptions of Social Justice and/or Social Consciousness as it Relates to Music Education	Riley, Patricia E.	2009	Excluded	Irrelavant Topic
It's not all about school: Ways of disrupting pre-service teachers' perceptions of pedagogy and communication	Ryan, M.; Healy, A.	2009	Excluded	Irrelavant Topic
How the gifted brain learns	Sousa, David A	2009	Excluded	Irrelavant Topic
Identifying the perceptions and experiences of adult students in	Tobin, Patricia Venecia	2009	Excluded	Irrelavant Topic

online art programs				
High-stakes testing and teacher burnout in public high school teachers	Tucker, Gail	2009	Excluded	Irrelavant Topic
Effect of the computer based game on pre-service teachers' achievement, attitudes, metacognition and motivation in chemistry	Tuysuz, C.	2009	Excluded	Irrelavant Topic
Teachers' Perceptions of Technology Integration in the United Arab Emirates School Classrooms	Abdurrahman Ghaleb, Almekhlafi; Farouq Ahmad, Almeqdadi	2010	Excluded	Irrelavant Topic
GULF TEACHERS' PERCEPTIONS OF TECHNOLOGY USE IN HIGHER EDUCATION	Bunts-Anderson, K.; Chova, L. G.; Belenguer, D. M.; Torres, I. C.	2010	Excluded	Irrelavant Topic
Social justice pedagogy across the curriculum: The practice of freedom	Chapman, Thandeka K; Hobbel, Nikola	2010	Excluded	Irrelavant Topic
Perceptions of a computer-based instruction system in special education: High school teachers and students views	Chiang, H. Y.; Jacobs, K.	2010	Excluded	Irrelavant Topic
THE MATHEMATICS' EDUCATION CHALLENGE IS TWO-FOLD, THE TEACHER'S CHALLENGE TO MAKE IT REAL, RELEVANT AND RIVETING AGAINST THE STUDENTS' CHALLENGE OF PRECONCEPTIONS, MISCONCEPTIONS AND NO PERCEPTION	D'Arcy-Warmington, A.; Chova, L. G.; Belenguer, D. M.; Torres, I. C.	2010	Excluded	Irrelavant Topic
The attitudes of student teachers towards educational technologies according to their status of receiving teaching application lessons	Alkan, Fatma; Erdem, Emine	2010	Included	
A study of the attitudes of university of North Carolina education faculty toward the use of computer-based simulation in pre-service teacher education methods courses	Englebert, Mary F.; Gretes, John A.	2010	Excluded	Irrelavant Topic
Pre-service teachers' perceptions about web 2.0 technologies	Baltaci-Goktalay, Sehnaz; Ozdilek, Zehra	2010	Included	
Comparison of the attitudes of the pre-service teachers from SESMTE and SESSTE departments against computer use (The case of Mugla University, Turkey)	Can, S.; Uzunboylu, H.	2010	Included	
Teacher candidates' real success situation on computers and their attitudes towards computer technology in the faculties of education	Ekizoglu, N.; Tezer, M.; Bozer, M.; Keser, H.; Ozcinar, Z.; Kanbul, S.	2010	Included	
Perceptions of prospective teachers towards technology use in class	Eyyam, R.; Menevis, I.; Dogruer, N.; Osam, U. V.; Vefali, G. M.;	2010	Included	

	Kufi, E. O.; Arkin, E. I.			
INVESTIGATION OF PRE-SERVICE TEACHERS' PERCEPTIONS ABOUT CONCEPT OF TECHNOLOGY THROUGH METAPHOR ANALYSIS	Gok, B.; Erdogan, T.	2010	Included	
A Needs Assessment Survey to Investigate Pre-Service Teachers' Knowledge, Experiences and Perceptions about Preparation to Using Educational Technologies	Koc, Mustafa; Bakir, Nesrin	2010	Included	
Literacy 2.0: Reading and writing in 21st century classrooms	Frey, Nancy; Fisher, Douglas	2010	Excluded	Irrelavant Topic
Assessing Problem Solving Strategy Differences within Online and Face-to-Face Courses and Their Relationship to Pre-Service Teachers' Competence and Confidence for Integrating Technology into Teaching	Peterson, Sharon L.	2010	Included	
A Path Analysis of Pre-Service Teachers' Attitudes to Computer Use: Applying and Extending the Technology Acceptance Model in an Educational Context	Teo, Timothy	2010	Included	
Measuring the Effect of Gender on Computer Attitudes among Pre-Service Teachers: A Multiple Indicators, Multiple Causes (MIMIC) Modeling	Teo, Timothy	2010	Included	
Exploring Attitudes towards Computer Use among Pre-Service Teachers from Singapore and the UK: A Multi-Group Invariance Test of the Technology Acceptance Model (TAM)	Teo, Timothy; Noyes, Jan	2010	Included	
Technology Integration in Secondary Physical Education: Teachers' Attitude and Practice	Gibbone, A.; Silverman, S. J.	2010	Excluded	Irrelavant Topic
Pre-Service English Language Teachers' Perceptions of Computer Self-Efficacy and General Self-Efficacy	Topkaya, Ece Zehir	2010	Included	
Teacher Education Students' Perceptions of the Value of Handouts Accompanying Teacher Educators' Computer-Generated Slide Presentations	Yilmazel-Sahin, Yesim; Oxford, Rebecca L.	2010	Included	
Pre-service chemistry teachers?€? attitude toward ICT in Xi?€?an	Zhou, Qing; Zhao, Yingmin; Hu, Jiani; Liu, Yang; Xing, Lijuan	2010	Included	
Achievement gap and sustainability: A case study of an elementary school bridging the achievement gap	Gray, Sandra Jean	2010	Excluded	Irrelavant Topic

TEACHERS' ATTITUDES TOWARD USE OF TECHNOLOGY: A RESEARCH FOR INFORMATICS TECHNOLOGY USE IN EDUCATION OF PRIMARY SCHOOLS	Gulluoglu, S. S.; Chova, L. G.; Belenguer, D. M.; Torres, I. C.	2010	Excluded	Irrelavant Topic
Early Childhood Pre-Service Teachers' Perceptions of Teaching Technology to Children in Japan and the United States	Izumi-Taylor, Satomi; Ito, Yoko; Gibbons, Andrew	2010	Excluded	Irrelavant Topic
Changing Teacher Practice Via Peer Facilitation: The Impact of a Peer Facilitator in a Collaborative Professional Development Model	Kheel, Marina Lang	2010	Excluded	Irrelavant Topic
The Effects of Computer-Assisted Instruction Designed According to 7E Model of Constructivist Learning on Physics Student Teachers' Achievement, Concept Learning, Self-Efficacy Perceptions and Attitudes	Kocakaya, Serhat; Gonen, Selahattin	2010	Excluded	Irrelavant Topic
Integrating Laptop Computers into Classroom: Attitudes, Needs, and Professional Development of Science Teachers—A Case Study	Klieger, Aviva; Ben-Hur, Yehuda; Bar-Yossef, Nurit	2010	Excluded	Irrelavant Topic
EXAMINING TEACHER CANDIDATES' ATTITUDES AND SELF-EFFICACY PERCEPTIONS TOWARDS THE COMPUTER ASSISTED EDUCATION	Kutluca, T.; Ekici, G.	2010	Excluded	Irrelavant Topic
Teaching the arts to engage English language learners	Latta, Margaret Macintyre; Chan, Elaine	2010	Excluded	Irrelavant Topic
PRE-SERVICE TEACHERS' ACADEMIC PERFORMANCE, ATTITUDE AND MOTIVATION TOWARD STUDYING A TECHNOLOGY COURSE IN A NON-NATIVE LANGUAGE	Almekhlafi, A. G.; Almazroui, K. M.; Chova, L. G.; Belenguer, D. M.; Torres, I. C.	2010	Excluded	Full Text Not Available
LIMITING THE EFFECTS OF THE MEDIA ON BODY IMAGE AND EATING ATTITUDES OF WOMEN PRE-SERVICE ELEMENTARY TEACHERS: A PILOT STUDY WITH NEW TECHNOLOGIES	Llorente, E.; de Eulate, L. P.; Chova, L. G.; Belenguer, D. M.; Torres, I. C.	2010	Excluded	Irrelavant Topic
Teacher value beliefs associated with using technology: Addressing professional and student needs	Ottenbreit-Leftwich, A. T.; Glazewski, K. D.; Newby, T. J.; Ertmer, P. A.	2010	Excluded	Irrelavant Topic
Setting for success: Student teachers' perceptions of the key elements that support successful, final year continuous placement experiences. It's relationships, not teaching skills!	Richardson, S.; Horsley, M.	2010	Excluded	Irrelavant Topic
Reviewing the relations between teachers' knowledge and pupils'	Rohaan, E. J.; Taconis, R.; Jochems, W. M. G.	2010	Excluded	Irrelavant Topic

attitude in the field of primary technology education				
The perceptions of students, teachers, and educational officers in Ghana on the role of computer and the teacher in promoting the first five principles of instruction	Sarfo, F. K.; Ansong-Gyimah, K.	2010	Excluded	Irrelavant Topic
Investigating preservice computer teachers' attitudes towards distance education	Simsek, A.; Iskenderoglu, T.; Iskenderoglu, M.; Keser, H.; Ozcinar, Z.; Kanbul, S.	2010	Excluded	Irrelavant Topic
The Attitudes of the Prospective Mathematics Teachers towards Instructional Technologies and Material Development Course	Uyangor, Sevinc Mert; Ece, Denizhan Karaca	2010	Excluded	Irrelavant Topic
A multi-case study of the demographic, culture, and climate characteristics of urban Christian schools that have narrowed the achievement gap in mathematics in grades 4 and 8	Washington, Cheryl Jefflyn Daniels	2010	Excluded	Irrelavant Topic
THE RELATIONSHIP BETWEEN PROSPECTIVE TEACHERS' COMPUTER LITERACY PERCEPTION AND THEIR ATTITUDES TOWARDS INTERNET USAGE	Yanik, C.	2010	Excluded	Non-English Language
AN APPROACH TO MAKING POSITIVE CHANGES IN PRE-SERVICE TEACHERS' ATTITUDES TOWARD MATHEMATICS AND OPERATING AND APPLICATION SKILLS FOR INFORMATION TECHNOLOGY DEVICES	Yoshida-Miyauchi, K.; Terashima, K.; Pinto, M. M. F.; Kawasaki, T. F.	2010	Excluded	Irrelavant Topic
Teacher Perceptions of Their Roles and Adoption of Educational Technology: Challenges in the Chinese Context	Zhu, Chang	2010	Excluded	Irrelavant Topic
IT TEACHERS' PERCEPTION ABOUT USING GAMES IN EDUCATION	Akilli, G. K.; Islim, O. F.; Sevim, N.; Torres, I. C.; Chova, L. G.; Martinez, A. L.	2011	Excluded	Irrelavant Topic
Exploring the goals, content, and methods of entrepreneurship professors: A multiple case study	Albornoz, Carlos A	2011	Excluded	Irrelavant Topic
Exploring Adult Digital Literacy Using Learners' and Educators' Perceptions and Experiences: The Case of the Second Chance Schools in Greece	Athanassios, Jimoyiannis; Maria, Gravani	2011	Excluded	Irrelavant Topic
The integration of technology within physical education teacher education: Perceptions of the faculty	Baert, Helena; Gorman, Dean	2011	Excluded	Irrelavant Topic
Biology teachers' perceptions about Science-Technology-Society (STS) education	Bettencourt, C.; Velho, J. L.; Almeida, P. A.; Elsevier Science, B. V.	2011	Excluded	Irrelavant Topic

Computer and instructional technologies preservice teachers' attitudes regarding distance education	Durmus, A.; Kaya, S.; Yalin, H. I.; Adiloglu, F.; Boz, H.; Karatas, S.; Ozdamli, F.	2011	Excluded	Irrelavant Topic
Teaching Students to Read Like Detectives: Comprehending, Analyzing and Discussing Text	Fisher, Douglas; Frey, Nancy	2011	Excluded	Irrelavant Topic
I ☺ Spanish: K–8 Attitudes toward Learning Spanish with Computers	Garc,; xed,; a-Villada, Eduardo	2011	Excluded	Irrelavant Topic
PERCEPTIONS ABOUT INFORMATION TECHNOLOGIES IN EDUCATION FROM SCHOOL TEACHERS IN LATIN AMERICA	Garcia-Urrea, S. C.; Chova, L. G.; Belenguer, D. M.; Martinez, A. L.	2011	Excluded	Irrelavant Topic
REIFICATION AND ABSTRACTION: DEVELOPING MATHEMATICAL CONTENT KNOWLEDGE THROUGH DISCOURSE AROUND ALGEBRAIC REASONING AND PROOF	Gillow-Wiles, Henry; Niess, Margaret L	2011	Excluded	Irrelavant Topic
PERCEPTIONS OF PRESERVICE TEACHERS REGARDING THE INTEGRATION OF INFORMATION AND COMMUNICATION TECHNOLOGIES IN TURKISH EDUCATION FACULTIES	Akbulut, Y.; Odabasi, H. F.; Kuzu, A.	2011	Included	
Age-related differences in ICT access and confidence among pre-service teachers	Albion, P. R.; Jamieson-Proctor, R.; Finger, G.	2011	Included	
Singaporean Pre-Service Teachers' Beliefs about Epistemology, Teaching and Learning, and Technology	Chai, Ching Sing; Wong, Benjamin; Teo, Timothy	2011	Included	
Perceived Usefulness, Perceive Ease of Use, Computer Attitude, and Using Experience of Web 2.0 Applications as Predictors of Intent to Use Web 2.0 by Pre-service Teachers for Teaching	Chiou, Yu-Fang; Franklin, Teresa	2011	Included	
TRAINEE TEACHERS' ATTITUDES ABOUT MATERIALS AND TECHNOLOGY USE IN MATHEMATICS EDUCATION	Dogan, M.; Yang, W. C.; Majewski, M.; De Alwis, T.; Karakirk, E.	2011	Included	
Investigation Of Relationship Between Preservice Teachers?€? Unethical Computer Using Behavior And Attitudes Towards The Using Of Internet	Kaya, Sinan; Durmus, Alpaslan	2011	Included	
Attitude of Teacher Candidates toward Making Computer Supported Education	Onder, F.; Celik, P.; Silay, I.; Karahoca, A.; Kanbul, S.	2011	Included	
A Mixed Methods Comparison of Teacher Education Faculty Perceptions of the Integration of Technology into Their Courses and	Teclehaimanot, Berhane; Mentzer, Gale; Hickman, Torey	2011	Included	

Student Feedback on Technology Proficiency				
An Assessment of the Influence of Perceived Enjoyment and Attitude on the Intention to Use Technology among Pre-Service Teachers: A Structural Equation Modeling Approach	Teo, Timothy; Noyes, Jan	2011	Included	
Investigating Pre-Service Early Childhood Teachers' Attitudes towards the Computer Based Education in Science Activities	Yilmaz, Nursel; Alici, Sule	2011	Included	
In Search of Pre-Service EFL Certificate Teachers' Attitudes towards Technology	Yuksel, G.; Kavanoz, S.; Karahoca, A.; Kanbul, S.	2011	Included	
into a Higher Education Curriculum	Mor, Yishay; Kuflik, Tsvika	2011	Excluded	Irrelavant Topic
Exploring the motivational orientations of graduate students in distance education programs	Nolot, Sandra K	2011	Excluded	Irrelavant Topic
STUDENT TEACHERS' PERCEPTIONS OF USING INFORMATION AND COMMUNICATION TECHNOLOGY IN KINDERGARTEN	Ihmeideh, F.; Chova, L. G.; Torres, I. C.; Martinez, A. L.	2011	Excluded	Full Text Not Available
A New ICT Curriculum for Primary Education in Flanders: Defining and Predicting Teachers' Perceptions of Innovation Attributes	Ruben, Vanderlinde; Johan van, Braak	2011	Excluded	Irrelavant Topic
Information on the Internet about colorectal cancer: Patient attitude and potential toward Web browsing. A prospective observational study	Sajid, M. S.; Shakir, A. J.; Baig, M. K.	2011	Excluded	Irrelavant Topic
Turkish primary school teachers' perceptions of school culture regarding ICT integration	Tezci, Erdo; x11f,; an,	2011	Excluded	Irrelavant Topic
A study of elementary school teachers' perceptions of the impact of direct instruction on closing the achievement gap for special education students in reading	Wright, Charlotte White	2011	Excluded	Irrelavant Topic
Assistive Technology Competencies of Teachers of Students with Visual Impairments: A Comparison of Perceptions	Zhou, L.; Smith, D. W.; Parker, A. T.; Griffin-Shirley, N.	2011	Excluded	Irrelavant Topic
The investigate attitude of primary pre-service teachers regarding science and technology laboratory	Acisli, S.; Metin, M.; Kolomu, A.; Baskan, G. A.; Ozdamli, F.; Kanbul, S.; Ozcan, D.	2012	Excluded	Irrelavant Topic
Analysing the Relationship between ICT Experience and Attitude toward E-Learning Comparing the Teacher and Student Perspectives	Akaslan, D.; Law, E. L. C.; Ravenscroft, A.; Lindstaedt, S.; Kloos, C. D.; HernandezLeo, D.	2012	Excluded	Irrelavant Topic

in Turkey				
Framing the adoption of serious games in formal education	Arnab, Sylvester; Berta, Riccardo; Earp, Jeffrey; De Freitas, Sara; Popescu, Maria; Romero, Margarida; Stanescu, Ioana; Usart, Mireia	2012	Excluded	Irrelavant Topic
BELIEFS OVER ICT IN PRE-SERVICE TEACHER: OPPORTUNITIES AND LIMITS FOR INNOVATION	Garrido, J. M.; Chova, L. G.; Torres, I. C.; Martinez, A. L.	2012	Excluded	Full Text Not Available
Motivational issues of faculty in Saudi Arabia	Cader, Akram Abdul	2012	Excluded	Irrelavant Topic
The Effect of Technology Based Course Material Use on the Science Teaching Self-Efficacy Beliefs of Pre-service Elementary Teachers	Cayci, B.	2012	Excluded	Irrelavant Topic
Pre-service students' perceptions and experiences of digital storytelling in diverse classrooms	Chigona, A.	2012	Excluded	Irrelavant Topic
The Development of Epistemic Relativism versus Social Relativism via Online Peer Assessment, and their Relations with Epistemological Beliefs and Internet Self-efficacy	Chin-Chung, Tsai	2012	Excluded	Irrelavant Topic
One Science Teacher's Professional Development Experience: A Case Study Exploring Changes in Students' Perceptions of Their Fluency with Innovative Technologies	Ebenezer, J.; Columbus, R.; Kaya, O. N.; Zhang, L.; Ebenezer, D. L.	2012	Excluded	Irrelavant Topic
Student Teachers of Technology and Design: Can Short Periods of STEM-Related Industrial Placement Change Student Perceptions of Engineering and Technology?	Gibson, Ken S.	2012	Excluded	Irrelavant Topic
Pre-Service Career and Technical Education Teachers' Perceptions of Their Subject Matter and Learning to Teach It: An Action Research Study	Green, Jeffrey M.	2012	Excluded	Irrelavant Topic
Are we protecting those who protect us? Stress and law enforcement in the 21 st century	Griffin, Jennifer Diehl	2012	Excluded	Irrelavant Topic
KNOWLEDGE AND ATTITUDES REGARDING THE USE OF EDUCATIONAL TECHNOLOGY AMONG TEACHER CANDIDATES AND COLLEGE PROFESSORS IN THE TEACHERS EDUCATION PROGRAM AT THE UNIVERSITY OF PUERTO RICO	Rosales-Mejia, W.; Vernaza-Hernandez, V.; Chova, L. G.; Martinez, A. L.; Torres, I. C.	2012	Excluded	Full Text Not Available
The effect of student teaching experience on preservice elementary teachers' self-efficacy beliefs for technology integration in the UAE	Al-Awidi, Hamed Mubarak; Alghazo, Iman Mohammad	2012	Included	

Digital Storytelling in Writing: A Case Study of Student Teacher Attitudes toward Teaching with Technology	Bumgarner, Barri L.	2012	Included	
Pre-Service Science and Technology Teachers' Efficacy Beliefs about Information and Communication Technologies (ICT) Usage and Material Design	Bursal, Murat; Yigit, Nevzat	2012	Included	
Student teachers' perception of the VBL system to enhance technology integration competencies	Chang, Y. F.; Chen, Y. C.; Hsu, C. L.; Croucher, Foundation; Sino-British Fellowship, Trust; Wu Jieh Yee Charitable, Foundation	2012	Included	
OPINIONS AND ATTITUDES OF STUDENTS TEACHERS' TOWARD ICT USE IN EDUCATION	Chisalita, O.; Cretu, C.; Frunzeti, T.; Jugureanu, R.; Ciolan, L.; Radu, C.	2012	Included	
Pre-Service Students' Perceptions and Experiences of Digital Storytelling in Diverse Classrooms	Condy, Janet; Chigona, Agnes; Gachago, Daniela; Ivala, Eunice; Chigona, Agnes	2012	Included	
Digital Opportunities within the Aboriginal Teacher Education Program: A Study of Preservice Teachers' Attitudes and Proficiency in Technology Integration	Dragon, Karon; Peacock, Kim; Norton, Yvonne; Steinhauer, Evelyn; Snart, Fern; Carbonaro, Mike; Boechler, Patricia	2012	Included	
Investigation of pre-service teachers' attitude s toward using the computer in teaching and learning mathematics	Duru, A.; Peker, M.; Birgin, O.	2012	Included	
Blog-Enhanced ICT Courses: Examining Their Effects on Prospective Teachers' ICT Competencies and Perceptions	Goktas, Yuksel; Demirel, Turgay	2012	Included	
Prospective EFL Teachers' Perceptions of ICT Integration: A Study of Distance Higher Education in Turkey	Hismanoglu, Murat	2012	Included	
The Impact of a Curricular Innovation on Prospective EFL Teachers' Attitudes towards ICT Integration into Language Instruction	Hismanoglu, Murat	2012	Included	
Exploring Taiwanese pre-service fitness trainers' attitudes toward ICT and their teaching self-confidence	Hsu, C. T.; Hsu, H. T.; Tsai, P. C.; Lee, M. H.	2012	Included	
PERCEPTIONS OF PROSPECTIVE INFORMATION TECHNOLOGIES TEACHERS TOWARDS FATIH PROJECT AND ITS COMPONENTS	Kaya, K. Y.; Tisoglu, S.; Ucak, S. S. K.; Kadioglu, E. A.; Chova, L. G.; Torres, I. C.; Martinez, A. L.	2012	Included	
Prospective Teachers' Perceptions of using Technology in Three Different Ways	Kobak, Mevhibe; Taskin, Nazli Ruya	2012	Included	
Preschool teachers' internet attitude and their internet self-efficacy: A	Lin, Y. H.; Liang, J. C.; International Institute of Applied,	2012	Included	

comparative study between pre-service and in-service teachers in taiwan	Informatics; Kyushu, University; Res. Inst. Inf. Technol. Kyushu, Univ			
Perceptions of Teachers of Students with Visual Impairments Regarding Assistive Technology: A Follow-up Study to a University Course	Kamei-Hannan, C.; Howe, J.; Herrera, R. R.; Erin, J. N.	2012	Excluded	Irrelavant Topic
Experiencing New Technology: Exploring Pre-Service Teachers' Perceptions and Reflections upon the Affordances of Social Media	Redman, Christine; Trapani, Fiona; Australian Association for Research in, Education	2012	Included	
Exploring Pre-Service Teachers' Beliefs about Using Web 2.0 Technologies in K-12 Classroom	Sadaf, Ayesha; Newby, Timothy J.; Ertmer, Peggy A.	2012	Included	
Student Teachers' Perceptions of Computerized Laboratory Practice For Science Teaching: A Comparative Analysis	Srisawasdi, Niwat	2012	Included	
The Impact of an Intensive Experience on Prospective Teachers' Perception of the Uses of Digital, Interactive Text among K-12 Students	Stonier, Francis W.	2012	Included	
Modelling the Influences of Beliefs on Pre-Service Teachers' Attitudes towards Computer Use	Teo, Timothy	2012	Included	
Determination of the Pre-Service Science and Classroom Teachers?€? Attitudes Towards Science Teaching and Technology and Relationship Between These Attitudes	Ugras, Mustafa; Altunbas, Seda; Ay, Kemalettin; Cil, Erol	2012	Included	
" They are here to stay": How rural northeast Alabama public elementary schools create an environment inclusive of Hispanic students a qualitative multiple case study	Keith, Leah	2012	Excluded	Irrelavant Topic
Pre-Service Biology Teachers' Attitudes towards ICT Using In Biology Teaching	Yapici, I. U.; Hevedanli, M.; Isman, A.; Liu, E. Z. F.; Kiyici, M.	2012	Included	
Relating Use of Digital Technology by Pre-Service Teachers to Confidence: A Singapore Survey	Yeung, Alexander Seeshing; Lim, Kam Ming; Tay, Eng Guan; Lam-Chiang, Audrey Cheausim; Hui, Chenri	2012	Included	
Use of social networking sites among shinas college of technology students in Oman	Kindi, Salim Said Ali Al; Alhashmi, Saadat M	2012	Excluded	Irrelavant Topic
Should Teachers Hold an Open Attitude to Students Using Digital Devices in the Classroom: A Pilot Study	Lam, P.; Tong, A. D.; Lam, P.	2012	Excluded	Irrelavant Topic

Elementary School Students' Perceptions of the New Science and Technology Curriculum by Gender	Mehmet Nuri, G.; xf,; mleksiz,	2012	Excluded	Irrelavant Topic
Developing an attitude scale towards using instructional technologies for pre-service teachers	Metin, M.; Kaleli Yilmaz, G.; Co??kun, K.; Biri????i, S.	2012	Excluded	Irrelavant Topic
Learning to Lead: Online Learning Principals' and Counselors' Perceptions of a District's Virtual School Support Services and Desires for Professional Development	Ross, Lisa Harrison	2012	Excluded	Irrelavant Topic
RELATIONSHIP BETWEEN TEACHERS' ICT COMPETENCY, CONFIDENCE LEVEL, AND SATISFACTION TOWARD ICT TRAINING PROGRAMMES: A CASE STUDY AMONG POSTGRADUATE STUDENTS	Tasir, Z.; Abour, K. M. E.; Abd Halim, N. D.; Harun, J.	2012	Excluded	Irrelavant Topic
TO INVESTIGATE OF SCIENCE - TECHNOLOGY TEACHERS' AND PROSPECTIVE TEACHERS' ATTITUDES TOWARDS TECHNOLOGY	Timur, B.; Timur, S.; Yilmaz, S.	2012	Excluded	Non-English Language
EFFECTS OF THE PROFESSIONAL DEVELOPMENT PROGRAM ON TURKISH TEACHERS: TECHNOLOGY INTEGRATION ALONG WITH ATTITUDE TOWARDS ICT IN EDUCATION	Uslu, O.; Bumen, N. T.	2012	Excluded	Irrelavant Topic
Learners' perceptions and illusions of adaptivity in computer-based learning environments	Vandewaetere, Mieke; Vandercruysse, Sylke; Clarebout, Geraldine	2012	Excluded	Irrelavant Topic
Identification of Stem Concepts Associated with Junior Livestock Projects: A Delphi Study	Wooten, Kate	2012	Excluded	Irrelavant Topic
The state of the African American male	Zamani-Gallaher, Eboni M; Polite, Vernon C	2012	Excluded	Irrelavant Topic
TEACHERS AND STUDENTS' PERCEPTION TOWARDS THE USE OF INFORMATION AND COMMUNICATION TECHNOLOGY FOR TEACHING ENGLISH IN BAHRAIN GOVERNMENTAL SECONDARY SCHOOLS	Ahmed, H. S.; Alajab, A. M.; Chova, L. G.; Martinez, A. L.; Torres, I. C.	2013	Excluded	Irrelavant Topic
The Reasons for Information Technologies Pre-Service Teachers to Prefer Teaching Profession and Their Attitudes towards the Profession	Akdag, Mustafa	2013	Excluded	Irrelavant Topic
Measuring performance excellence: Key performance indicators for	Ballard, Paul J	2013	Excluded	Irrelavant Topic

institutions accepted into the Academic Quality Improvement Program (AQIP)				
EXAMINING A MIDDLE SCHOOL SCIENCE OLYMPIAD PROGRAM THROUGH SELF-STUDY	Barr, Donald J	2013	Excluded	Irrelavant Topic
Adoption and use of technology in early education The interplay of extrinsic barriers and teacher attitudes	Blackwell, C. K.; Lauricella, A. R.; Wartella, E.; Robb, M.; Schomburg, R.	2013	Excluded	Irrelavant Topic
Seeing eye-to-eye on ICT: Science student and teacher perceptions of laptop use across 14 Australian schools	Crook, S. J.; Sharma, M. D.; Wilson, R.; Muller, D. A.	2013	Excluded	Irrelavant Topic
Seeing through the eyes of African-American female math teachers: Their counter-narratives and how it influences their math identity	DuRant, Winnie Lashaun	2013	Excluded	Irrelavant Topic
Developing a New Computer Game Attitude Scale for Taiwanese Early Adolescents	Eric Zhi-Feng, Liu; Chun-Yi, Lee; Jen-Huang, Chen	2013	Excluded	Irrelavant Topic
Middle School Special Education Teachers' Perceptions and Use of Assistive Technology in Literacy Instruction	Flanagan, S.; Bouck, E. C.; Richardson, J.	2013	Excluded	Irrelavant Topic
ICT INTEGRATION IN TEACHER EDUCATION: PAKISTAN "ATTITUDE OF PROSPECTIVE TEACHERS TOWARDS ICT"	Tahira, S. S.; Saadi, A. M.; Chova, L. G.; Martinez, A. L.; Torres, I. C.	2013	Excluded	Full Text Not Available
Student Teachers' Perceptions about the Impact of Internet Usage on Their Learning and Jobs	Gialamas, Vasilis; Nikolopoulou, Kleopatra; Koutromanos, George	2013	Excluded	Irrelavant Topic
The Brave New Online World of Teens and a Call to Action for Educators	Heslip, Joseph Charles	2013	Excluded	Irrelavant Topic
Examining the Potential Use of Geospatial-Informatics Technologies to Engage Northern Canadian First Nation Youth in Environmental Initiatives	Isogai, Andrea Danielle	2013	Excluded	Irrelavant Topic
Investigation of pre-service teachers' self-efficacy beliefs of educational Internet use	Kahraman, S.; Yilmaz, Z. A.; Erkol, M.; Yal??in, S. A.	2013	Excluded	Irrelavant Topic
THE PERCEPTIONS OF STUDENTS TOWARDS THE CLASSROOM MANAGEMENT TECHNIQUES USED BY SCIENCE AND TECHNOLOGY TEACHERS	Kiraz, A.; Omag, K.	2013	Excluded	Irrelavant Topic
Pre-Service ESL teachers' perceptions of parody integration in digital stories	Abdul Latiff, A.; Mat Daud, N.	2013	Included	

Using constructivist and collaborative approach to enhance pre-service teachers' attitude toward computer in computer course: Learning and using MS Excel functions in problem-based scenarios	Efendioglu, A.; Berkant, H. G.; Cukurova, B.; Uzunboylu, H.; Ozdamli, F.	2013	Included	
On the Cusp of Change: Examining Pre-Service Teachers' Beliefs about ICT and Envisioning the Digital Classroom of the Future	Fluck, A.; Dowden, T.	2013	Included	
INTEGRATING LEARNING TECHNOLOGY INTO THE CLASSROOM: THE IMPORTANCE OF PRE-SERVICE TEACHERS' AND LECTURERS' PERCEPTIONS	Fredrick, S.; Andrew, L.; Chova, L. G.; Martinez, A. L.; Torres, I. C.	2013	Included	
Drawing on technology: An investigation of preservice teacher beliefs in the context of an introductory educational technology course	Funkhouser, Beverly J.; Mouza, Chrystalla	2013	Included	
Turkish and Singaporean Pre-service Physics Teachers' Beliefs about Teaching and Use of Technology	Gurcay, D.; Wong, B.; Chai, C. S.	2013	Included	
The Gamer Generation Teaches School: The Gaming Practices and Attitudes towards Technology of Pre-Service Teachers	Hayes, Elisabeth; Ohrnberger, Maryellen	2013	Included	
Pre-Service Teachers' Perception of and Technology Competency at Creating and Using E-Picture Books	Park, Yong Joon; Yang, Youjin	2013	Included	
Teacher-Education Student Perceptions for Stages of Concern Related to Integrating Technology	Quadrini, Virginia Horak	2013	Included	
Changing mindsets: The attitude of pre-service teachers on technology for teaching	Sabiescu, A.; Van Zyl, I.; Pucciarelli, M.; Cantoni, L.; Bytheway, A.; Chigona, W.; Tardini, S.; International Development Research, Centre; Google,; Ibm,; Ipid,; Microsoft,	2013	Included	
The ECE Pre-Service Teachers' Perception on Factors Affecting the Integration of Educational Computer Games in Two Conditions: Selecting versus Redesigning	Sancar Tokmak, Hatice; Ozgelen, Sinan	2013	Included	
Pre-Service ELT Teachers' Attitudes Towards Computer Use: A Turkish Survey	Saricoban, A.	2013	Included	
The Effect of Dynamic Software on Prospective Mathematics Teachers' Perceptions Regarding Information and Communication Technology	Tatar, Enver	2013	Included	
Student Teachers' Attitude towards ePortfolios and Technology in	Tur, G.; Marin, V. I.; GarciaPenalvo, F. J.	2013	Included	

Education				
NOTE FOR EDITOR: iPad Learning Ecosystem: Developing Challenge-Based Learning Using Design Thinking	Marin, Catalina; Hargis, Jace; Cavanaugh, Cathy	2013	Excluded	Irrelavant Topic
'Just enough to make you take it seriously': exploring students' attitudes towards peer assessment	McGarr, Olliver; Clifford, Amanda Marie	2013	Excluded	Irrelavant Topic
Prospective biology, physics and chemistry teachers' attitudes toward information and communication technologies	Ozarslan, M.; ??etin, G.; Sarita??, T.	2013	Excluded	Non-English Language
STUDENT AND TEACHER PERCEPTIONS OF COMPUTERS AND MOBILE DEVICES FOR FOREIGN LANGUAGE LEARNING	Pagel, J. W.; Lambacher, S. G.; Chova, L. G.; Martinez, A. L.; Torres, I. C.	2013	Excluded	Irrelavant Topic
The effects of online professional development on higher education teachers' beliefs and intentions towards learning facilitation and technology	Rienties, B.; Brouwer, N.; Lygo-Baker, S.	2013	Excluded	Irrelavant Topic
Changing Computer Programming Education; The Dinosaur that Survived in School An explorative study about educational issues based on teachers' beliefs and curriculum development in secondary school	Rolandsson, L.; Ieee,	2013	Excluded	Irrelavant Topic
Study site experiences and attitudes toward prospective assessments of suicidal ideation and behavior in clinical trials: Results of an internet-based survey	Stewart, M.; Butler, A.; Alphs, L.; Chappell, P. B.; Feltner, D. E.; Lenderking, W. R.; Mahableshwarkar, A. R.; Makumi, C. W.; DuBrava, S.	2013	Excluded	Irrelavant Topic
THE EFFECTS OF COMPUTER AIDED TEACHING APPLICATIONS ON GRAPHICAL SKILL, ATTITUDE AND PERFORMANCES OF PRE-SERVICE TEACHERS	Uyan, T.; Onen, A. S.	2013	Excluded	Non-English Language
The Use of Confidence Intervals as a Meta-Analytic Lens to Summarize the Effects of Teacher Education Technology Courses on Preservice Teacher TPACK	Young, Jamaal R.; Young, Jemimah L.; Hamilton, Christina	2013	Excluded	Irrelavant Topic
Pre-service teachers' attitudes toward technology: Scale development study	Aydin, F.; Karaa, F. N.	2014	Excluded	Irrelavant Topic
Pre-service EFL Teacher's Attitudes towards Computer Assisted Language Learning (CALL)	Ba????z, Tutku; ??ubuk??u, Feryal	2014	Excluded	Irrelavant Topic
Open Participatory Engagement Network (OPEN): An instructional	Baker III, Fredrick W	2014	Excluded	Irrelavant Topic

design meta-framework for creating participatory networked learning environments				
Policies related to the implementation of openness at research intensive universities in the United States: a descriptive content analysis	Baker III, Fredrick William	2014	Excluded	Irrelavant Topic
Don't let little Johnny keep you from your money: A critical analysis of the implementation of neoliberal accountability on an elementary school	Beach, Mary G	2014	Excluded	Irrelavant Topic
Analyzing the Attitudes of Physical Education and Sport Teachers towards Technology	Bisgin, H.	2014	Excluded	Irrelavant Topic
The new ecology of biliteracy in California: An exploratory study of the early implementation of the State Seal of Biliteracy	DeLeon, Tanya M	2014	Excluded	Irrelavant Topic
Burnout in Prospective Elementary School Teachers: Is It Related to Reasons for Choosing the Elementary School Teaching Major, Beliefs about the Teaching Career and Satisfaction with the Choice?	Dundar, Sahin	2014	Excluded	Irrelavant Topic
The Relationships among Chinese Practicing Teachers' Epistemic Beliefs, Pedagogical Beliefs and Their Beliefs about the Use of ICT	Feng, Deng; Ching Sing, Chai; Chin-Chung, Tsai; Min-Hsien, Lee	2014	Excluded	Irrelavant Topic
Newman's Error Analysis and mathematical language: Diagnosing mathematical errors on word problems made my 4th graders who attend a low SES school	Flagg, Valerie Lemon	2014	Excluded	Irrelavant Topic
Changing teachers, changing students? The impact of a teacher-focused intervention on students' computer usage, attitudes, and anxiety	Gibson, P. A.; Stringer, K.; Cotten, S. R.; Simoni, Z.; O'Neal, L. J.; Howell-Moroney, M.	2014	Excluded	Irrelavant Topic
Transition from brick and mortar to online teaching: Middle-grade teachers' perspectives	Griffin, Cathy	2014	Excluded	Irrelavant Topic
The relationship between the humanistic values of pre-service teachers of information technologies and their attitudes towards teaching profession	Gunduz, S.; Ao, S. I.; Burgstone, J.; Ao, S. I.; Douglas, C.; Grundfest, W. S.; Douglas, C.; Burgstone, J.; Ao, S. I.; Iaeng Society of Artificial Intelligence; Iaeng Society of Bioinformatics; Iaeng Society of Computer Science; Iaeng Society of Data Mining; Iaeng Society of Electrical Engineering; et al.,	2014	Excluded	Irrelavant Topic
Learning Modalities and Delivery Systems for Officer Professional	Hadziomerovic, Aida; Vollick, Shaun; Budgell, Glen	2014	Excluded	Irrelavant Topic

Development Period 3: Literature Review				
The Determining the Relationship between Turkish Language Teachers' Attitudes towards Computer Education and Technology	Kahraman, E.; Iseri, K.; Unal, E.	2014	Excluded	Irrelavant Topic
Perception of Primary School Students, Parents and Teachers toward the Use of Computers, the Internet and Social Networking sites	Kanthawongs, P.; Kanthawongs, P.; Chatsupakul, K.; Kamwachirapitak, R.	2014	Excluded	Irrelavant Topic
Patterns of Peer Interaction and Mechanisms Governing Social Network Structure in Two Massively Open Online Courses for Educators	Kellogg, Shaun B	2014	Excluded	Irrelavant Topic
"It Is Like Putting Fire in the Children's Hands": A Comparative Case Study of Pre-Service Teachers' Knowledge of and Beliefs about Education for Democracy in an Established and Emerging Post-Conflict Democracy	Lanahan, Brian K.; Phillips, Michele S.	2014	Excluded	Irrelavant Topic
The Impact of Project-Based Learning on Pre-Service Teachers' Technology Attitudes and Skills	Alexander, Curby; Knezek, Gerald; Christensen, Rhonda; Tyler-Wood, Tandra; Bull, Glen	2014	Included	
Analysis of self-efficacy perception of the science teacher candidates intended for information technology: Example of Sakarya University faculty of education	Balcin, M. D.; Ari, E.; Erdogan, Y.; Besoluk, S.	2014	Included	
Technology and Engineering Education Doctoral Students' Perceptions of Their Profession	Martin, Gene; Ritz, John; Kosloski, Michael	2014	Excluded	Irrelavant Topic
The Impact of Authentic Learning Exercises on Pre-Service Teachers' Motivational Beliefs towards Technology Integration	Banas, Jennifer R.; York, Cindy S.	2014	Included	
Closing the Gap Between Attitudes and Perceptions About ICT-Enhanced Learning Among Pre-service STEM Teachers	Barak, Miri	2014	Included	
Pre-service EFL teachers' attitudes towards Computer Assisted Language Learning (CALL)	Basoz, T.; Cubukcu, F.; Laborda, J. C.; Ozdamli, F.; Maasoglu, Y.	2014	Included	
Student teachers' attitudes and beliefs towards using ICT within inclusive education and practice	Beacham, N.; McIntosh, K.	2014	Included	
Pre-service elementary education teachers self-efficacy beliefs regarding technology integration in the classroom environment: A case study	Blakeney, Kimberly P.; Larson, Mark L.; Warren, Elizabeth	2014	Included	

An investigation of special education teachers' perceptions of the effectiveness of a systematic 7-step Virtual Worlds Teacher Training Workshop for increasing social skills	Nussli, Natalie	2014	Excluded	Irrelavant Topic
ICT USE AND ATTITUDES AMONG SECONDARY EDUCATION TEACHERS IN ROMANIA	Ogrezeanu, A.; Ogrezeanu, A.; Roceanu, I.	2014	Excluded	Irrelavant Topic
THE PERCEPTION OF THE PHYSICAL EDUCATION TEACHERS CONCERNING THE USE OF THE AUDIOVISUAL TECHNOLOGIES IN SHAPING THE MOTOR SKILLS	Constantin, P.; Sgem,	2014	Included	
CONTEMPORARY PRACTICES OF TECHNOLOGY AND ITS AFFORDANCES: PERCEPTIONS OF PRE-SERVICE TEACHERS ON THE UTILIZATION OF TECHNOLOGY IN TEACHING AND LEARNING PRACTICE	Delaney, S.; Trapani, F.; Chandler, P.; Redman, C.; Chova, L. G.; Martinez, A. L.; Torres, I. C.	2014	Included	
Pre-Service Teachers' Perceptions of the Internet and Online Courses: The Case of an American Pacific Island University	Inoue-Smith, Yukiko	2014	Included	
The Relationship between Attitudes of Prospective Physical Education Teachers towards Education Technologies and Computer Self-Efficacy Beliefs	Kalemoglu Varol, Yaprak	2014	Included	
Changing Pre-Service Mathematics Teachers' Beliefs about Using Computers for Teaching and Learning Mathematics: The Effect of Three Different Models	Karatas, Ilhan	2014	Included	
Attitudes and beliefs student teachers hold toward technology integration	Karr, Darci L.; Steckelberg, Allen	2014	Included	
"I can do it": Does confidence and perceived ability in learning new ICT skills predict pre-service health professionals' attitude towards engaging in e-healthcare?	Lam, M. K.; Nguyen, M.; Lowe, R.; Nagarajan, S. V.; Lincoln, M.; Schaper, L. K.; Grain, H.; Martin-Sanchez, F.	2014	Included	
Enhancing pre-service teachers' self-efficacy beliefs for technology integration through lesson planning practice	Lee, Youngju; Lee, Jihyun	2014	Included	
Technology Integration in a Science Classroom: Preservice Teachers' Perceptions	Rehmat, Abeera P.; Bailey, Janelle M.	2014	Included	
Assessing the perceptions of prospective teachers related to computer skills according to various variables	Tuncer, M.; Kaysi, F.	2014	Included	

Pre-service mathematics teachers' knowledge development and belief change within a technology-enhanced mathematics course	Zambak, Vecihi Serbay; Tyminski, Andrew M.	2014	Included	
Learning with their peers: Using a virtual learning community to improve an in-service Biology teacher education program in Brazil	Rolando, Luiz Gustavo Ribeiro; Salvador, Daniel F??bio; Souza, Andr?? Henrique Silva; Luz, Mauricio RMP	2014	Excluded	Irrelavant Topic
The Long Shadow of Doctoral Candidate Status. Case Study-Poland	Szadkowski, Krystian	2014	Excluded	Irrelavant Topic
Enhancing dialogue to reduce transactional distance: a case of using mobile mediated social media in a virtual group activity	Tunjera, Nyarai	2014	Excluded	Irrelavant Topic
Deciding on Science: An Analysis of Higher Education Science Student Major Choice Criteria	White, Stephen Wilson	2014	Excluded	Irrelavant Topic
Assessing computer attitudes: Does it matter for teacher education in developing countries?	Wong, S. L.; Ogata, H.; Lomicka-Anderson, L.; Chai, C. S.; Hampel, R.; Hayashi, Y.; Vassileva, J.; Liu, C. C.; Chen, W.; Hsu, J.; Lan, Y. J.; Mason, J.; Yamada, M.; Shyu, H. Y.; Weerasinghe, A.; Wu, Y. T.; Zhang, L.; Kinshuk,; Matsubara, Y.; Miao, Y.; Ogata, H.; Kong, S. C.; Chang, M.; Jong, M. S. Y.; Kuo, R.; Robson, R.; Wasson, B.; Kashihara, A.; Cress, U.; Jansen, M.; Oshima, J.; Yin, C.; Zhang, J.; Chinn, C.; Kansai-Osaka st Century Association; Kddi Foundation; Support Center for Advanced Telecommunications Technology Research, Foundation; Tateisi, Science; Technology, Foundation; The Telecommunications Advancement, Foundation; et al.,	2014	Excluded	Irrelavant Topic
Variables Predicting Prospective Biology Teachers' Acceptance Perceptions Regarding Gene Technology	Yilmaz, Mirac; Demirhan, Haydar	2014	Excluded	Irrelavant Topic
UTILIZING A MOODLE-BASED E-LEARNING PLATFORM IN ELT: AN AUTOETHNOGRAPHY	Anas, Ismail	2015	Excluded	Irrelavant Topic
Exploring the dual-natured impact of digital technology on student-classroom engagement in a Texas public high school	Ayers, Joseph J	2015	Excluded	Irrelavant Topic
Internationalizing higher education: Language matters	Bamond Lozano, Victoria M; Strotmann, Birgit	2015	Excluded	Irrelavant Topic
Cross-cultural comparison of motor competence in children from Australia and Belgium	Bardid, Farid; Rudd, James Robert; Lenoir, Matthieu; Polman, Remco; Barnett, Lisa M	2015	Excluded	Irrelavant Topic
"We Make It Controversial": Elementary Preservice Teachers' Beliefs about Race	Buchanan, Lisa Brown	2015	Excluded	Irrelavant Topic

Improving Science Student Teachers' Self-perceptions of Fluency with Innovative Technologies and Scientific Inquiry Abilities	Calik, M.; Ebenezer, J.; Ozsevgec, T.; Kucuk, Z.; Artun, H.	2015	Excluded	Irrelavant Topic
Pre-service teachers' developing technological pedagogical content knowledge (Tpack) and beliefs on the use of technology in the k-12 mathematics classroom: A review of the literature	Crompton, H.	2015	Excluded	Irrelavant Topic
The Relationship between Primary School Teacher and Student Attitudes towards Science and Technology	Denessen, E.; Vos, N.; Hasselman, F.; Louws, M.	2015	Excluded	Irrelavant Topic
How Are The Pre-Service Teachers' Attitude Levels Towards Educational Measurement And Evaluation Course And Does It Differ According To The Demographic Characteristics?	Dogan, N.; Kara, Y.; Uzunboylu, H.	2015	Excluded	Irrelavant Topic
Teacher attitudes and best practices with ICT faculty Adult Continuing Education in Andalusia	Fernandez Batanero, J. M.; Torres Gonzalez, J. A.	2015	Excluded	Irrelavant Topic
Teacher Attitudes towards ICT in the Context of Inclusive Education	Fernandez-Batanero, J. M.; Ruiz, M. J. C.; Alves, G. R.; Felgueiras, M. C.	2015	Excluded	Irrelavant Topic
UX of social network Edmodo in undergraduate engineering students	G??mez, Ang??lica; Ruiz, ??ngel Alberto Magre????n; Orcos, Lara	2015	Excluded	Irrelavant Topic
The Transformative Experience in Engineering Education	Goodman, Katherine Ann	2015	Excluded	Irrelavant Topic
I just do not have time for new ideas: resistance, resonance and micro-mobilisation in a teaching community of practice	Houghton, Luke; Ruutz, Aaron; Green, Wendy; Hibbins, Ray	2015	Excluded	Irrelavant Topic
Relationships between teachers ' pedagogical beliefs, subject cultures, and mediation practices of students' use of digital technology	Karaseva, A.; Siibak, A.; Pruulmann-Vengerfeldt, P.	2015	Excluded	Irrelavant Topic
Integrated use of multiple social software tools and face-to-face activities to support self-regulated learning: a case study in a higher education context	Laru, Jari; J??rvel??, Sanna	2015	Excluded	Irrelavant Topic
PRE-SERVICE TEACHER BELIEFS AND ATTITUDES RELATED TO ICT FOR SCIENCE EDUCATION AT EARLY YEARS. A PRELIMINARY STUDY	Quesada, A.; Ariza, M. R.; Chova, L. G.; Martinez, A. L.; Torres, I. C.	2015	Excluded	Full Text Not Available
A Framework for Implementing Inquiry-Based Learning in the Elementary Classroom	Lister, Christopher Andrew Paul	2015	Excluded	Irrelavant Topic
From Doing to Being: Nurturing Professional Learning Communities	MacKinney, Bennett	2015	Excluded	Irrelavant Topic

With Peer Observation				
Catalina MARIN, MEd Faculty, Higher Colleges of Technology (HCT) Abu Dhabi Women's College (ADWC) Abu Dhabi, UAE	MARIN, Catalina; HARGIS, Jace; CAVANAUGH, Cathy	2015	Excluded	Irrelavant Topic
Uso de las TIC's en el dise??o y validaci??n de contenidos pedag??gicos de Anatom??a y Fisiolog??a enfocados al aprendizaje de t??cnicas procedimentales de Enfermer??a como profesi??n	Moreano Barrag??n, Elsie Paulina	2015	Excluded	Irrelavant Topic
Self-belief and confidence to teach arts and digital technology in K-6 classrooms: Perspectives from pre-service teachers	Narelle, L.; Susanne, G.	2015	Excluded	Irrelavant Topic
The Effects of Irrational Beliefs on Academic Motivation and Academic Self-Efficacy of Candidate Teachers of Computer and Instructional Technologies Education Department	Ozer, E. A.; Akgun, O. E.; Alevriadou, A.	2015	Excluded	Irrelavant Topic
Understanding Instructors Curriculum Planning Process for the Refinement and Dissemination of a Digital Platform to Share Transportation Education Materials	Peters, Allie	2015	Excluded	Irrelavant Topic
Pre-Service Teachers' Perceptions of ICT Integration in Teacher Education in Turkey	Aslan, Aydin; Zhu, Chang	2015	Included	
Exploring Student Teachers' Perceptions of the Influence of Technology in Learning and Teaching Mathematics	Bansilal, Sarah	2015	Included	
Computer anxiety and attitudes toward using internet in english language classes among iranian postgraduate student teachers	Bolandifar, S.; Noordin, N.	2015	Included	
Investigation of Pre-Service Physical Education Teachers' Attitudes Towards Computer Technologies (Case of Turkey)	Can, Suleyman	2015	Included	
The correlation between pre-service teachers?€? attitudes towards technology and achievement in material design course	Findikoglu, F.; Alci, B.; Karatas, H.	2015	Included	
Modeling pre-service teachers' perception of future internet usage for professional educational purposes	Gungoren, O. C.; Horzum, M. B.	2015	Included	
Using Digital technology-generated dynamic visualization in science education-perceptions of pre-service science teachers	Kahraman, S.; Demir, Y.; Demir, N.	2015	Included	
Digital Literacy for Primary Teachers	Savage, Moira	2015	Excluded	Irrelavant Topic
TPACK Competencies and Technology Integration Self-Efficacy	Keser, Hafize; Karaoglan Yilmaz, Fatma Gizem; Yilmaz, Ramazan	2015	Included	

Perceptions of Pre-Service Teachers				
The pedagogic beliefs of Indonesian teachers in inclusive schools	Sheehy, Kieron; Budiyanto	2015	Excluded	Irrelavant Topic
Learning Styles and Perceptions of Student Teachers of Computer-Supported Collaborative Learning Strategy Using Wikis	Li, Kai Ming	2015	Included	
The Relationship between Pre-Service Teachers' Basic Technology Competence, Technology Self-Efficacy and Perceptions of Adopting Educational Applications on iPads for Classroom Use	Martindale, Rebecca; Gartin, Barbara C.	2015	Included	
Pre-service science teachers?€? perceptions of technology literacy	Ozkan, G.; Tombak, B.	2015	Included	
Evaluating Pre-Service Teachers' Perceptions of Technology-Enhanced Field Experiences	Peterson, Leah; Jacobs, Howard	2015	Included	
After-hours mobile technology use and its effect on burnout experienced by student affairs professionals	Stark, Anne R	2015	Excluded	Irrelavant Topic
Effects of Creating Digital Stories on Foreign Language Education Pre-Service Teachers' TPACK Self-Confidence	Sancar-Tokmak, Hatice; Yanpar-Yelken, Tugba	2015	Included	
Perceptions of teachers regarding the implementation of the internet in education	Stosic, L.; Stosic, I.	2015	Excluded	Irrelavant Topic
Teacher students' perceptions of their digital competence	Svensson, M.; Baelo, R.; Soare, E.; Langa, C.	2015	Included	
Digital storytelling as a tool for teaching: Perceptions of pre-service teachers	Tiba, C.; Condy, J.; Chigona, A.; Tunjera, N.	2015	Included	
Predictive Power of Prospective Physical Education Teachers' Attitudes towards Educational Technologies for Their Technological Pedagogical Content Knowledge	Varol, Yaprak Kalemoglu	2015	Included	
The investigation of attitude and readiness of information and communication technologies pre-service teachers toward web based learning	Yagci, M.; Sirakaya, D. A.; Ozudogru, G.; Iaman, A.; Eskicumali, A.	2015	Included	
The Relationships among Pre-Service Mathematics Teachers' Beliefs about Mathematics, Mathematics Teaching, and Use of Technology in China	Yang, Xinrong; Leung, Frederick K. S.	2015	Included	
Beliefs about Educational Technology: a view from a pre-service teacher biography in school and university	Varela, F. F.; Barujel, A. G.	2015	Excluded	Non-English Language

Planting the seeds of change: Examining the role of a civic organization in teaching youth about citizenship in post-revolutionary Egypt	Waly, Salma Gehad	2015	Excluded	Irrelavant Topic
Improving schools through data-based decision making: an assessment of data use in primary schools in Ethiopia	YIBRIE, AHMED	2015	Excluded	Irrelavant Topic
INSTRUCTIONAL SCREENCAST: A RESEARCH CONCEPTUAL FRAMEWORK	ABDULRAZAK, Muhammad Razuan; MOHAMADALI, Ahmad Zamzuri	2016	Excluded	Irrelavant Topic
Perceptions of Arabic language teachers toward their use of technology at the Omani basic education schools	Al Musawi, A.; Al Hashmi, A.; Kazem, A. M.; Al Busaidi, F.; Al Khaifi, S.	2016	Excluded	Irrelavant Topic
Integration of digital technologies into play-based pedagogy in Kuwaiti early childhood education: teachers' views, attitudes and aptitudes	Aldhafeeri, F.; Palaiologou, I.; Folorunsho, A.	2016	Excluded	Irrelavant Topic
Design guidelines for Arabic mobile learning application based on cognitive theory of multimedia learning	Al-Rikabi, Mohammed Abbas Neamah	2016	Excluded	Irrelavant Topic
Impact of STS Issue Oriented Instruction on Pre-Service Elementary Teachers' Views and Perceptions of Science, Technology, and Society	Amirshokoohi, Aidin	2016	Excluded	Irrelavant Topic
Transforming Assessment: Using a Rubric Approach Effectively to Support Learning	Anderson, Steven; Graham, S	2016	Excluded	Irrelavant Topic
The reality of STEM education, design and technology teachers' perceptions: a phenomenographic study	Bell, D.	2016	Excluded	Irrelavant Topic
Identifying and Formulating Teachers' Beliefs and Motivational Orientations for Computer Science Teacher Education	Bender, Elena; Schaper, Niclas; Caspersen, Michael E.; Margaritis, Melanie; Hubwieser, Peter	2016	Excluded	Irrelavant Topic
Development and validation of the anthropogenic climate change dissenter inventory	Bentley, Andrew PK; Petcovic, Heather L; Cassidy, David P	2016	Excluded	Irrelavant Topic
THE WAYS CHARACTER STRENGTHS SUPPORT K?€?8 MATHEMATICS?€? AND THE COMMON CORE STATE STANDARDS	Bier, Melinda C; Berkowitz, Marvin W; Sherblom, Stephen A; Coulter, Bob	2016	Excluded	Irrelavant Topic
Effect of Inquiry-Based Computer Simulation Modeling on Pre-Service Teachers' Understanding of Homeostasis and Their Perceptions of Design Features	Chabalengula, Vivien; Fateen, Rasheta; Mumba, Frackson; Ochs, Laura Kathryn	2016	Excluded	Irrelavant Topic

A reliability generalization of the parental authority questionnaire	Dean, Lynn M	2016	Excluded	Irrelavant Topic
Exploring the use of educational technology in primary education: Teachers' perception of mobile technology learning impacts and applications' use in the classroom	Domingo, M. G.; Gargante, A. B.	2016	Excluded	Irrelavant Topic
Student-teachers' pedagogical beliefs: Learner-centred or teacher-centred when using ICT in the science classroom?	du Plessis, A.	2016	Excluded	Irrelavant Topic
FUTURE TEACHERS' DIGITAL COMPETENCE: WHAT IS THE PERCEPTION OF CURRENT STUDENTS TEACHERS'?	Esteve-Mon, F. M.; Gisbert-Cervera, M.; Lazaro-Cantabrana, J. L.	2016	Excluded	Non-English Language
FROM STUDENT-TEACHERS TO NOVICE TEACHERS: CHANGING ATTITUDES TOWARDS INTEGRATION OF TECHNOLOGY IN TEACHING MATHEMATICS	Gurevich, I.; Stein, H.; Gorev, D.; Chova, L. G.; Martinez, A. L.; Torres, I. C.	2016	Excluded	Full Text Not Available
Chemistry Teachers' Journey through Modeling Instruction: From Workshop to Classroom	Frick, Tasha	2016	Excluded	Irrelavant Topic
Opportunities for learning given to prospective mathematics teachers: between ritual and explorative instruction	Heyd-Metzuyanim, Einat; Tabach, Michal; Nachlieli, Talli	2016	Excluded	Irrelavant Topic
"I wonder what you know?" teachers designing requests for factual information	Houen, Sandy; Danby, Susan; Farrell, Ann; Thorpe, Karen	2016	Excluded	Irrelavant Topic
Perceptions of teacher education candidates to digital and learning technologies	Bull, P. H.; Mansaray, M.; Patterson, G. C.; Dunston, Y.	2016	Excluded	Full Text Not Available
Collaborative Learning and Support Environment for Teachers in Native American Pueblo Schools in New Mexico	Kilde, Josephine	2016	Excluded	Irrelavant Topic
University student and teacher perceptions of teacher roles in promoting autonomous language learning with technology outside the classroom	Lai, C.; Yeung, Y.; Hu, J. J.	2016	Excluded	Irrelavant Topic
Why Do Students Use Mobile Technology for Social Purposes during Class? Modeling Teacher Credibility, Learner Empowerment, and Online Communication Attitude as Predictors	Ledbetter, A. M.; Finn, A. N.	2016	Excluded	Irrelavant Topic
Pre-service teachers and technology integration: International cases and generational attitudes toward technology in education	Mulder, D. J.	2016	Excluded	Full Text Not Available
Making learning authentic: An educational case study describing	Lund, Stephanie	2016	Excluded	Irrelavant Topic

student engagement and motivation in a project-based learning environment				
Awarding teaching excellence: 'what is it supposed to achieve?' Teacher perceptions of student-led awards	Madriaga, M.; Morley, K.	2016	Excluded	Irrelavant Topic
EXPLORING ATTITUDES TO ICT AMONGST PRE-SERVICE TEACHERS ON ENTRY ONTO A TEACHER EDUCATION PROGRAMME IN SPAIN	McGarr, O.; Gavaldon, G.; Chova, L. G.; Martinez, A. L.; Torres, I. C.	2016	Excluded	Irrelavant Topic
Evaluating teachers?€? professional development initiatives: towards an extended evaluative framework	Merchie, Emmelien; Tuytens, Melissa; Devos, Geert; Vanderlinde, Ruben	2016	Excluded	Irrelavant Topic
Too Important to Ignore: A Post-Intentional Phenomenological Investigation of Teaching Pre-Service Early Childhood Teachers About Infants and Toddlers	Pearson, Jolene A	2016	Excluded	Irrelavant Topic
Designing the Myth: Pattern Language to Assist with the Designing of Garments at the Drawing Stage Oblikovanje mita: jezikovni vzorci kot pomo?? pri oblikovanju obla??il v fazi risanja	Purgaj, Jure; Jev??nik, Simona	2016	Excluded	Irrelavant Topic
Constructivist and cognitive multimedia learning theories as tools for training teachers on how to integrate technology using the TPACK framework for teaching Arabic online	Rasha, Essam	2016	Excluded	Irrelavant Topic
Attitudes to and Understanding of Risk of Acquisition of HIV Over Time: Design and Methods for an Internet-based Prospective Cohort Study Among UK Men Who Have Sex With Men (the AURAH2 Study)	Sewell, J.; Speakman, A.; Phillips, A. N.; Cambiano, V.; Lampe, F. C.; Gilson, R.; Asboe, D.; Nwokolo, N.; Clarke, A.; Ogilvy, A.; Collins, S.; Rodger, A. J.	2016	Excluded	Irrelavant Topic
Teachers and Students' Perception of Mobile Technology in Teaching and Learning	Sotiloye, B.; Bodunde, H.; Adebiyi, A.; Aduradola, R.; Bernadas, C.; Minchella, D.	2016	Excluded	Irrelavant Topic
TEACHERS' USE AND ATTITUDES OF ICT IN HIGHER EDUCATION: EXPLORING CAMPUS AND DISTANCE EDUCATION	Svensson, L.; Lundin, J.; Chova, L. G.; Martinez, A. L.; Torres, I. C.	2016	Excluded	Irrelavant Topic
Research on the Development of Middle School Mathematics Pre-Service Teachers' Perceptions Regarding the Use of Technology in Teaching Mathematics	Akkaya, Recai	2016	Included	
Saudi arabian science and mathematics pre-service teachers'	Alblaihed, Munthir Abdullah	2016	Included	

perceptions and practices of the integration of technology in the classroom				
Digital Storytelling and Its Tools for Language Teaching: Perceptions and Reflections of Pre-Service Teachers	Asik, A.	2016	Included	
Attitudes of Turkish EFL Student Teachers towards Technology Use	Baz, Esra Harmandaoglu	2016	Included	
The attitudes of pre-service teachers attending the schools of physical education and sports of the universities in Turkey towards education technologies	Can, S.	2016	Included	
A Comparison of Swiss and Turkish Pre-Service Science Teachers' Attitudes, Anxiety and Self-Efficacy Regarding Educational Technology	Efe, H??lya Aslan; Efe, Rifat; Y??cel, Sait	2016	Included	
The impact of a paired grouping pre-service technology integration course on student participant attitudes, proficiency, and technological knowledge toward technology	Giles, Linda Michelle; Tyler-Wood, Tandra	2016	Included	
Comparison of pre-service and in-service teachers' attitudes and perceived abilities toward integrating digital technologies into the classroom	Gomez, Jeannette R.; Garrett, Sherrye D.; Kouzekanani, Kamiar	2016	Included	
Perceptions of Technology Engagement on Culturally Responsive Pre-Service Teachers	Greene-Clemons, Cheresa Denae	2016	Included	
TEACHERS' ATTITUDES TOWARDS TECHNOLOGY RICH EDUCATION IN BULGARIA	Terzieva, V.; Todorova, K.; Kademova-Katzarova, P.; Andreev, R.; Chova, L. G.; Martinez, A. L.; Torres, I. C.	2016	Excluded	Irrelavant Topic
A Technology Integration Education (TIE) Model for Millennial Preservice Teachers: Exploring the Canonical Correlation Relationships Among Attitudes, Subjective Norms, Perceived Behavioral Controls, Motivation, and Technological, Pedagogical, and Content Knowledge (TPACK) Competencies	Holland, D. D.; Piper, R. T.	2016	Included	
The relation between the attitudes of pre-service physical education teachers towards instructional technologies and material design course and their academic standing: Batman university sample	I??ikg??z, E.	2016	Included	
Effects of a Technology-Friendly Education Program on Pre-Service Teachers' Perceptions and Learning Styles	Kim, Dong-Joong; Choi, Sang-Ho	2016	Included	

Analysis of Turkish Prospective Science Teachers' Perceptions on Technology in Education	Koksal, Mustafa Serdar; Yaman, Suleyman; Saka, Yavuz	2016	Included	
Gameful design in the development of asynchronous online discussion activities: A case study	Trest, William Michael	2016	Excluded	Irrelavant Topic
Modelling Serbian pre-service teachers' attitudes towards computer use: A SEM and MIMIC approach	Teo, Timothy; Milutinovi??, Verica; Zhou, Mingming	2016	Included	
From OER to Open Education: Perceptions of Student Teachers after Creating Digital Stories with Creative Common Resources	Tur, G.; Urbina, S.; Moreno, J.	2016	Excluded	Irrelavant Topic
Effect of information technologies (It) pre-service teachers?€? learning approaches on their attitude towards programing	Yagci, M.	2016	Included	
Exploring Prospective English Language Teachers' Perceptions of the "Internet" through Metaphorical Conceptualizations	Yaman, Ismail	2016	Included	
Pre-Service Training and ICT Implementation in the Classroom: ELT Teachers' Perceptions	Zyad, Hicham	2016	Included	
Teacher self-efficacy, academic self-efficacy, and computer self-efficacy as predictors of attitude toward applying computer-supported education	Yesilyurt, E.; Ulas, A. H.; Akan, D.	2016	Excluded	Irrelavant Topic
Teacher perceptions on the use of digital gamified learning in tourism education: The case of South African secondary schools	Adukaite, A.; van Zyl, I.; Er, S.; Cantoni, L.	2017	Excluded	Irrelavant Topic
Perceptions of pre-service english teachers towards computer assisted language learning course	Akayo??lu, S.	2017	Excluded	Irrelavant Topic
Computer usage & constructivist approach of the basic class: Abilities & skills and attitudes of pre-service teachers	Alawi, G. A. A. A.; Shwal, M.; Nasreen, N.; Dalian Maritime, University	2017	Included	
The Tension in Pre-Service Teachers' Explorations of Tablet Technology for Literacy Purposes: Positive Beliefs and Practical Shortcomings	Al-Hazza, Tami Craft	2017	Included	
Perception of Pre-Service Teachers' towards the Teaching Practice Programme in College of Technology Education, University of Education, Winneba	Amankwah, Francis; Oti-Agyen, Philip; Sam, Francis Kwame	2017	Excluded	Irrelavant Topic
Personalized Adaptive Learning Impact on the Student Experience:	Asaad, Diana	2017	Excluded	Irrelavant Topic

Stakeholders' Perspectives				
The relationship among pre-service teachers' computer competence, attitude towards computer-assisted education, and intention of technology acceptance	Baturay, M. H.; G??k??earslan, ??; Ke, F.	2017	Included	
Associations among Teachers' Attitudes towards Computer-Assisted Education and TPACK Competencies	Baturay, M. H.; Gokcearslan, S.; Sahin, S.	2017	Excluded	Irrelavant Topic
Transforming Pre-Service Teachers' Beliefs and Understandings about Design and Technologies	Best, Marnie	2017	Included	
Investigating the attitudes of pre-service teachers towards technology based on various variables	Birkollu, S. S.; Yucesoy, Y.; Baglama, B.; Kanbul, S.	2017	Included	
Developing Signalong Indonesia: issues of happiness and pedagogy, training and stigmatisation	Budiyanto; Sheehy, Kieron; Kaye, Helen; Rofiah, Khofidotur	2017	Excluded	Irrelavant Topic
MUSICAL EDUCATION AND DIGITAL TECHNOLOGIES: THE USE OF LEARNING OBJECTS AND THE PERCEPTION OF TEACHERS	Chamorro, A.; Gitahy, R. R. C.; Tercariol, A. A. D.; dos Santos, D. A. D.	2017	Excluded	Irrelavant Topic
"You Can Do it Anywhere": Student and Teacher Perceptions of an Online Sexuality Education Intervention	Chen, E.; Barrington, C.	2017	Excluded	Irrelavant Topic
Kwaliteiten van Mbo-docenten voor Passend Onderwijs	Dekker, Ineke	2017	Excluded	Irrelavant Topic
Supporting K-12 Online Learners: Developing a Mentorship Program	Edwards, Clea	2017	Excluded	Irrelavant Topic
Perceptions of Digital Competency among Student Teachers: Contributing to the Development of Student Teachers' Instructional Self-Efficacy in Technology-Rich Classrooms	Elstad, Eyvind; Christophersen, Knut-Andreas	2017	Included	
Comparability in Balanced Assessment Systems for State Accountability	Evans, Carla M; Lyons, Susan	2017	Excluded	Irrelavant Topic
Pre-service teachers' perceptions of the competence dimensions of digital literacy and of psychological and educational measures	Garc??a-Mart??n, Judit; Garc??a-S??nchez, Jes??s-Nicasio	2017	Included	
Pre-Service Teachers' Attitude towards Information and Communication Technology Usage: A Ghanaian Survey	Gyamfi, Stephen Adu	2017	Included	
The Effect of Student Teaching Experience and Teacher Beliefs on Pre-Service Teachers' Self-Efficacy and Intention to Use Technology	Han, Insook; Shin, Won Sug; Ko, Yujung	2017	Included	

in Teaching				
The Relationship among Pre-Service EFL Teachers' Beliefs about Language Learning, Pedagogical Beliefs, and Beliefs about ICT Integration	Inayati, Dian; Emaliana, Ive	2017	Included	
A Comprehensive Analysis on Technopedagogical Education Competency and Technology Perception of Pre-Service Teachers: Relation, Levels and Views	Incik, Eylem Yalcin; Akay, Cenk	2017	Included	
Teacher Beliefs Regarding Learning, Pedagogy, and the Use of Technology in Higher Education	Jaaskela, P.; Hakkinen, P.; Rasku-Puttonen, H.	2017	Excluded	Irrelavant Topic
A Critical Examination of Senior Executive Leadership Succession Planning and Management with Implications for Underrepresented Minorities	Jackson, LeKeisha D	2017	Excluded	Irrelavant Topic
Cultivating Critical Mindsets in the Digital Information Age: Teaching Meaningful Web Evaluation	Johnson, Angela Kwasnik	2017	Excluded	Irrelavant Topic
An Investigation of Technological Pedagogical Content Knowledge, Self-Confidence, and Perception of Pre-Service Middle School Mathematics Teachers towards Instructional Technologies	Karatas, Ilhan; Tunc, Mutlu Piskin; Yilmaz, Nurbanu; Karaci, Gulzade	2017	Included	
Perception and Possibilities of ICT Tools in the Education from the Teachers' Perspective	Klement, M.; Dostal, J.; Bartek, K.	2017	Excluded	Irrelavant Topic
De Zelfgestuurde Leerdoelen en de Deelname aan Leeractiviteiten van startende, ervaren en zeer ervaren Leerkrachten in het Primair Onderwijs	Knoop, Natalie	2017	Excluded	Irrelavant Topic
A Review of the Importance of Peer Instruction Argumentative Strategy (PIAS) in Science Learning	Kola, Aina Jacob	2017	Excluded	Irrelavant Topic
Educational Process: International Journal	Kola, Aina Jacob	2017	Excluded	Irrelavant Topic
Differences and developments in attitudes and self-efficacy of prospective chemistry teachers concerning the use of ICT in education	Krause, M.; Pietzner, V.; Dori, Y. J.; Eilks, I.	2017	Included	
Aging Workers in Changing Labor Markets and Career Learning	Krekanova, Vera	2017	Excluded	Irrelavant Topic
Students' perceptions of teacher impact on their self-directed	Lai, C.; Li, X. S.; Wang, Q.	2017	Excluded	Irrelavant Topic

language learning with technology beyond the classroom: cases of Hong Kong and US				
Therapeutic education before Ramadan: Can it change the beliefs of the Moroccan diabetic patient and dissuade him from fasting? (Prospective study about 190 patients)	Laidi, S.; El Aziz, S.; Chadli, A.	2017	Excluded	Irrelavant Topic
Risky-play at school. Facilitating risk perception and competence in young children	Lavrysen, Ann; Bertrands, Els; Leyssen, Leene; Smets, Lieve; Vanderspikken, Anja; De Graef, Peter	2017	Excluded	Irrelavant Topic
Teach it to sustain it! Environmental attitudes of Hungarian teacher training students in Serbia	Major, L.; Namestovski, Z.; Horak, R.; Bagany, A.; Krekic, V. P.	2017	Excluded	Irrelavant Topic
Urban High School Teachers' Perceptions of Their Pre-service Training in Classroom Management Strategies	Martin, Jeff Huber	2017	Excluded	Irrelavant Topic
Digital competence in students of educational degrees. Analysis of future teachers' competence and perception	Mendez, V. G.; Martin, A. R.; Rodriguez, M. D. M.	2017	Excluded	Irrelavant Topic
Early Career Teacher Candidate TPACK Development: Implementation of a Learning Activity Types Short Course	Mourlam, Daniel; Bleecker, Heather	2017	Excluded	Irrelavant Topic
Pre-Service and Mentor Teachers' Perceptions Regarding the Level of Technology Integration in the Curriculum	Moye, Gatsy A.	2017	Excluded	Irrelavant Topic
E-Portfolio Assessment in Measuring Soft Skills in Teacher Education Program: Preliminary Findings	Muhammad, Azliza; Lebar, Othman; Mokshein, Siti Eshah; Baharom, Sadiah	2017	Excluded	Irrelavant Topic
Participation and Performance on Paper-and Computer-Based Low-Stakes Assessments	Nissen, Jayson M; Jariwala, Manher; Close, Eleanor W; Van Dusen, Ben	2017	Excluded	Irrelavant Topic
The impact of digital stories on preservice teacher beliefs about English language learners	Pappamihiel, N. Eleni; Ousley-Exum, Denise; Ritzhaupt, Albert	2017	Excluded	Irrelavant Topic
Inside the ?€?body box?€?: exploring feedback in higher education	Pentassuglia, Monica	2017	Excluded	Irrelavant Topic
Managing Digital Learning Environments: Student Teachers' Perception on the Social Networking Services Use in Writing Courses in Teacher Education	Prasojo, Lantip Diat; Habibi, Akhmad; Mukminin, Amirul; Muhaimin,; Taridi, Muhammad; Ikhsan,; Saudagar, Ferdiaz	2017	Included	
Education in cyberculture: teachers' perceptions about the integration of digital technologies in higher education	Rabello, C. R. L.; Tavares, K. C. D.	2017	Excluded	Non-English Language
Pre-Service Teachers' Self-Efficacy Beliefs towards Educational	Raphael, Christina; Mtebe, Joel S.	2017	Included	

Technologies Integration in Tanzania				
Integrating ICT into teacher education programs from a TPACK perspective: Exploring perceptions of university lecturers	Reyes Jr, Vicente Chua; Reading, Christine; Doyle, Helen; Gregory, Sue	2017	Excluded	Irrelavant Topic
Implementing Project SIED: Special Education Teachers' Perceptions of a Simplified Technology Decision-Making Process for App Identification and Evaluation	Schmidt, M. M.; Lin, M. F. G.; Paek, S.; MacSuga-Gage, A.; Gage, N. A.	2017	Excluded	Irrelavant Topic
Ethics, Epistemologies, and Inclusive Pedagogy	Sheehy, Kieron	2017	Excluded	Irrelavant Topic
A Case Study on the Facilitation of Positive Behavior Interventions and Supports in a Public Elementary School	Shumway, John Todd	2017	Excluded	Irrelavant Topic
Understanding the relationship between teachers' pedagogical beliefs and technology use in education: a systematic review of qualitative evidence (vol 65, pg 555, 2017)	Tondeur, J.; van Braak, J.; Ertmer, P.; Ottenbreit-Leftwich, A.	2017	Excluded	Irrelavant Topic
Internet and democracy: Is the Internet an important predictor for physical education teacher candidates' attitudes towards democracy?	Unlu, H.	2017	Excluded	Irrelavant Topic
Enhancing Students’ Computer Programming Performances, Critical Thinking Awareness and Attitudes towards Programming<br>An Online Peer-Assessment Attempt	Wang, Xiao-Ming; Hwang, Gwo-Jen; Liang, Zi-Yun; Wang, Hsiu-Ying	2017	Excluded	Irrelavant Topic
Factors Influencing training effectiveness: Evidence from public sector in Bahrain	Yaqoot, Ehsan Saeed; Wan Mohd Noor, Wan Shakizah; Mohd Isa, Mohd Faizal	2017	Excluded	Irrelavant Topic
Towards a Strategic Blend in Education: A review of the blended learning literature	Yeigh, Tony; Sell, Ken; Lynch, David; Willis, Royce; Smith, Richard; Provost, Steve; Turner, David	2017	Excluded	Irrelavant Topic
A Case Study on Specialised Content Knowledge Development with Dynamic Geometry Software: The Analysis of Influential Factors and Technology Beliefs of Three Pre-Service Middle Grades Mathematics Teachers	Zambak, Vecihi S.; Tyminski, Andrew M.	2017	Included	
?€?Intellectual challenge is as necessary as breathing?€?: An Interview with Laurence Wright	Pearce, Interviewer?€?Brian		Excluded	Irrelavant Topic
A mixed research-based model for pre-service science teachers' digital literacy: Responses to "which beliefs" and "how and why they interact" questions	Gunes, E.; Bahcivan, E.	2018	Included	

An Investigation Into the Gamification of E-Learning in Higher Education	Annansingh, Fenio	2018	Excluded	Irrelavant Topic
An Investigation of Pre-Service Primary School Teachers' Attitudes towards Digital Technology and Digital Citizenship Levels in Terms of Some Variables	Ciftci, Serdar; Aladag, Soner	2018	Included	
ClassBeacons: Designing Distributed Visualization of Teachers- Physical Proximity in the Classroom	An, Pengcheng; Bakker, Saskia; Ordanovski, Sara; Taconis, Ruurd; Eggen, Berry	2018	Excluded	Irrelavant Topic
I will do it but religion is a very personal thing?€?: teacher education applicants?€? attitudes towards teaching religion in Ireland	Heinz, M.; Davison, K.; Keane, E.	2018	Excluded	Irrelavant Topic
Mapping elementary school students' creativity in science process skills of life aspects viewed from their divergent thinking patterns	Subali, Bambang; Paidi, Paidi; Mariyam, Siti		Excluded	Irrelavant Topic
Student Teachers' Perceptions on Educational Technologies' Past, Present and Future	Orhan Goksun, Derya; Filiz, Ozan; Kurt, Adile Askim	2018	Included	
The importance of attitudes toward technology for pre-service teachers' technological, pedagogical, and content knowledge: Comparing structural equation modeling approaches	Scherer, Ronny; Tondeur, Jo; Siddiq, Fazilat; Baran, Evrim	2018	Included	
Using Gartner?€?s Hype Cycle as a basis to analyze research on the educational use of ubiquitous computing	Laru, Jari; J??rvel??, Sanna		Excluded	Irrelavant Topic
Virtual Reality & Augmented Reality in het primair onderwijs	de Lange, Robin; Lodewijk, Maarten		Excluded	Irrelavant Topic
