# Supplementary material for: Integrating ICT in education: A scoping review of pre-service teachers’ ICT beliefs
Source: PLoS One. 2025 Feb 7;20(2):e0317591. doi: 10.1371/journal.pone.0317591 (PMC11805408; doi:10.1371/journal.pone.0317591)
Supplement: S3 Table — (PDF) [file pone.0317591.s003.pdf]

Integrating ICT in education: A scoping review of pre-service teachers' ICT beliefs

S3 Table. Overview of Included Studies

Author	Year	Title	Study Types	Topic Categories	Research Methods	Types of ICT	Subject	Settings	Regions	Demographic Info	Training Needs
Avsec	2018	Pre-service teachers' attitudes towards technology, engagement in active learning, and creativity as predictors of ability to innovate	Journal Article	ICT Beliefs	Quantitative	General	Unspecified	Primary School	Slovenia	Yes	No
Adelana	2023	Exploring pre-service teachers' intention to use virtual reality: A mixed method approach	Journal Article	ICT Beliefs	Mixed	virtual reality	Educational Technology	Unspecified	Nigeria	Yes	Yes
Amador	2021	Prospective teachers' appraisals of technology platforms: comparing perception and complexity	Journal Article	ICT Beliefs	Mixed	LessonSketch & GoAnimate	Math	Mixed	United States	No	No
Bahcivan	2019	Investigating the Relations Among Pre-Service Teachers' Teaching/Learning Beliefs and Educational Technology Integration Competencies: a Structural Equation Modeling Study	Journal Article	ICT Beliefs Modeling	Quantitative	General	Unspecified	Unspecified	Turkey	No	No
Aşik	2018	Digital storytelling and	Journal	ICT Beliefs	Mixed	Digital	Language	Unspecified	Turkey	Yes	No

		its tools for language teaching: Perceptions and reflections of pre-service teachers	Article			Storytelling					
Benzer	2019	The effect of computer-aided 3D modeling activities on pre-service teachers' spatial abilities and attitudes towards 3d modeling	Journal Article	ICT Beliefs	Quantitative	3D modeling	Computer	Unspecified	Turkey	Yes	Yes
Brändle	2023	Self-assessments, attitudes, and motivational orientations towards the use of digital media in teaching a comparison between student teachers of different subject clusters	Journal Article	ICT Beliefs Comparison	Quantitative	digital media	Mixed	Unspecified	German	No	Yes
Farjon	2019	Technology integration of pre-service teachers explained by attitudes and beliefs, competency, access, and experience	Journal Article	ICT Beliefs	Quantitative	General	Mixed	Secondary School	Netherland	Yes	No
Genol	2023	Spanish Trainees Teachers' Attitudes Toward the Use of Technology in Education: Variables Involved	Journal Article	ICT Beliefs	Quantitative	General	Unspecified	Unspecified	Spain	Yes	Yes
Küsel	2023	Investigating U.S. and German pre-service	Journal Article	ICT Beliefs	Quantitative	General	Mixed	Mixed	Germany and the	Yes	Yes

		teachers' beliefs regarding digital technology							US		
Lee	2018	Internet-based epistemic beliefs, engagement in online activities, and intention for constructivist ICT integration among pre-service teachers	Journal Article	ICT Beliefs	Quantitative	Internet	Unspecified	Primary School	China	Yes	No
Merjovaara	2024	Early childhood pre-service teachers' attitudes towards digital technologies and their relation to digital competence	Journal Article	ICT Beliefs	Quantitative	General	Unspecified	Pre-school	Finland	Yes	No
Baek	2020	Pre-service teachers' perception of technology competencies based on the new ISTE technology standards	Journal Article	Technology Competence and Literacy	Mixed	General	Mixed	Primary School	South Korea	Yes	No
Bates	2019	The Perception of Pennsylvania Pre-Service Teachers Regarding Their Preparedness to Utilize and Integrate Educational Technology into the Classroom	Journal Article	ICT Beliefs	Quantitative	General	Mixed	Mixed	United States	Yes	Yes
Wu	2023	The effects of a gamified online course on pre-service teachers'	Journal Article	ICT Beliefs	Quantitative	General	Unspecified	Unspecified	United States	No	No

		confidence, intention, and motivation in integrating technology into teaching									
Başal	2020	Perceptions of pre-service English teachers towards the use of digital badges	Journal Article	ICT Beliefs	Mixed	digital badges	Language	Unspecified	Turkey	Yes	No
AitAli	2023	Exploring the TPACK of prospective nursing educators: A national study	Journal Article	ICT Beliefs	Quantitative	General	Health	Unspecified	Morocco	Yes	No
Blume	2020	Games people (don't) play: An analysis of pre-service EFL teachers' behaviors and beliefs regarding digital game-based language learning	Journal Article	Perception of ICT Based Teaching & Learning	Quantitative	Game	Language	Mixed	Germany	No	No
Anbalagan	2021	A study of B. Ed. Student Teachers' Attitudes Towards Digital Libraries in Madurai District	Journal Article	ICT Beliefs	Quantitative	Digital Library	Unspecified	Unspecified	India	Yes	No
Belda-Medina	2022	Integrating augmented reality in language learning: pre-service teachers' digital competence and attitudes through the TPACK framework	Journal Article	ICT Beliefs	Mixed	Augmented Reality	Language	Unspecified	Spain	Yes	Yes
BUENO-	2021	Pre-service teachers'	Journal	ICT Beliefs	Mixed	General	Health	Primary	Spain	No	Yes

ALAST UEY		perceptions and training contributions towards ICT use	Article					School			
Camilleri	2021	Student teachers and their attitudes towards ICT: Lessons learned from three different countries	Journal Article	ICT Beliefs	Mixed	General	Unspecified	Unspecified	Malta, Norway, and Spain	No	No
Atabek	2020	Pre-school and primary school pre-service teachers' attitudes towards using technology in music education	Journal Article	ICT Beliefs	Quantitative	General	Music	Mixed	Turkey	Yes	No
Dalim	2019	Digital storytelling for 21st century learning: A study on pre-service teachers' perception	Journal Article	ICT Beliefs	Quantitative	Digital Storytelling	Unspecified	Unspecified	Malaysia	Yes	No
Choi	2024	Pre-service English teachers' beliefs and practices in the use of digital technology: the case of a technology-enhanced teacher preparation course in South Korea	Journal Article	ICT Beliefs	Qualitative	General	Language	Unspecified	South Korea	No	
Avifah	2022	Pre-service EFL teachers' perception on educational video production technology: A needs analysis	Journal Article	ICT Beliefs	Quantitative	Video Production Technology	Language	Unspecified	Indonesi a	Yes	No

Awofala	2019	Attitudes toward computer, computer anxiety and gender as determinants of pre-service science, technology, and mathematics teachers' computer self-efficacy	Journal Article	ICT Beliefs	Quantitative	Computer	STEM	Unspecified	Nigeria	Yes	No
Essuman	2024	Perceptions of technology integration in algebra instruction among pre-service mathematics teachers: a case study in the Upper East region of Ghana	Journal Article	ICT Beliefs	Quantitative	General	Math	Unspecified	Ghana	No	Yes
Bakir	2020	Attitudes of prospective Turkish language teachers towards instructional technologies and material development course	Journal Article	ICT Beliefs	Quantitative	General	Language	Unspecified	Turkey	Yes	No
Bengel	2021	Modern technology in geography education—attitudes of pre-service teachers of geography on modern technology	Journal Article	ICT Beliefs	Quantitative	General	Geography	Unspecified	Germany	Yes	Yes
Çoban	2022	Using virtual reality technologies in STEM education: ICT pre-service teachers'	Journal Article	ICT Beliefs	Mixed	virtual reality	STEM	Unspecified	Turkey	Yes	No

		perceptions									
Dinc	2019	Prospective teachers' perceptions of barriers to technology integration in education	Journal Article	ICT Beliefs	Qualitative	General	Unspecified	Primary School	United States	No	Yes
Dong	2021	Pre-service early childhood teachers' attitudes and intentions: young children's use of ICT	Journal Article	ICT Beliefs	Mixed	General	Unspecified	Pre-school	China	Yes	Yes
Eyal	2023	Pre-Service Teachers' Attitudes toward Integrating Digital Games in Learning as Cognitive Tools for Developing Higher-Order Thinking and Lifelong Learning	Journal Article	ICT Beliefs	Mixed	digital games	Unspecified	Unspecified	Israel	Yes	No
Doğru	2020	An investigation of pre-service visual arts teachers' perceptions of computer self-efficacy and attitudes towards web-based instruction	Journal Article	Perception of ICT Based Teaching & Learning	Quantitative	visual arts	Art	Unspecified	Turkey	Yes	No
Fořtová	2021	"And My Screen Wouldn't Share...": efl Student-Teachers' Perceptions of ict in Online Teaching Practice and Online Teaching	Journal Article	ICT Beliefs	Qualitative	General	Language	Secondary School	Czeck	Yes	No
Hossain	2021	A Study of Pre-Service	Journal	ICT Beliefs	Quantitative	General	Unspecified	Unspecified	India	Yes	No

		Student's Attitude Towards Use of Helpful Technology in Teaching Learning Process	Article								
Gavaldon	2019	Exploring pre-service teachers' future intentions to use technology through the use of comics	Journal Article	ICT Beliefs	Qualitative	General	Unspecified	Unspecified	Spain	No	No
Heath	2021	What pre-service teacher technology integration conceals and reveals: "Colorblind" technology in schools	Journal Article	ICT Beliefs	Qualitative	General	Mixed	Unspecified	United States	Yes	No
Hu	2022	Pre-service teachers' perceptions of adopting digital games in education: A mixed methods investigation	Journal Article	ICT Beliefs	Mixed	digital games	Unspecified	Unspecified	United States	Yes	Yes
Gan	2022	Pre-service EFL teachers' motivational beliefs about instructional use of technology: development and validation of a scale	Journal Article	ICT Beliefs	Quantitative	General	Language	Secondary School	China	No	No
Juandi	2023	The perception of prospective mathematics teachers towards technology use in learning during the Covid-19 pandemic	Journal Article	ICT Beliefs	Quantitative	General	Math	Unspecified	Indonesia	No	No

Istifci	2018	Perceptions of pre-service english teachers on using information communication technologies	Proceedings	ICT Beliefs	Mixed	General	Unspecified	Unspecified	Turkey	Yes	Yes
Izgi-Onbasili	2022	AN EXAMINATION OF PRE-SERVICE TEACHERS' EXPERIENCES IN CREATING A SCIENTIFIC DIGITAL STORY IN THE CONTEXT OF THEIR SELF CONFIDENCE IN TECHNOLOGICAL PEDAGOGICAL CONTENT KNOWLEDGE	Journal Article	ICT Beliefs	Mixed	General	Unspecified	Primary School	Turkey	Yes	No
Gloria	2016	COMPUTER SELF-EFFICACY AND ATTITUDE OF UNIVERSITY OF IBADAN PRE-SERVICE TEACHERS TOWARDS THE OF USE DIGITAL STORYTELLING AS A DELIVERY METHOD OF INSTRUCTION	Journal Article	ICT Beliefs	Mixed	Digital Storytelling	Mixed	Unspecified	Nigeria	No	No
Kuo	2024	An exploratory study of pre-service teachers'	Journal Article	ICT Beliefs	Quantitative	digital games	Unspecified	Unspecified	United States	Yes	No

		perceptions of technological pedagogical content knowledge of digital games									
Huda	2018	Investigating pre-service teachers about their competencies, experiences, and attitudes towards technology integration	Proceedings	ICT Beliefs	Quantitative	General	Mixed	Unspecified	Indonesia	Yes	No
Kapranov	2020	English goes digital: Framing pre-service teachers' perceptions of a learning management system in their EFL studies	Journal Article	ICT Beliefs	Qualitative	Learning Management System	Language	Unspecified	Norway	Yes	No
Karlsudd	2018	Cheating or legitimate support? Student-Teachers' attitudes toward digital tools in school	Journal Article	ICT Beliefs	Mixed	General	Unspecified	Mixed	Sweden	Yes	Yes
Lee	2020	Initial teacher education students' perceptions of technology and technology education in New Zealand	Journal Article	ICT Beliefs	Quantitative	General	Unspecified	Mixed	New Zealand	Yes	No
Luik	2019	Estonian in-service teachers' and pre-service teachers' perceptions of content, pedagogy, and	Proceedings	ICT Beliefs	Quantitative	General	Unspecified	Mixed	Estonia	Yes	No

		technology knowledge, based on the TPACK framework									
Luo	2023	The seewo interactive whiteboard (IWB) for ESL teaching: How useful it is?	Journal Article	ICT Beliefs	Qualitative	interactive whiteboard	Language	Unspecified	United States	Yes	Yes
Kulakhmetova	2022	Prospective teachers' attitude towards computer algebra systems (CAS) and their choice of using CAS in solving problems of systems of differential equations	Journal Article	ICT Beliefs	Quantitative	computer algebra systems	Math	Secondary School	Kazakhstan	No	No
Mertala	2019	Digital technologies in early childhood education - a frame analysis of preservice teachers' perceptions	Journal Article	ICT Beliefs	Qualitative	General	Unspecified	Pre-school	Finland	Yes	No
Laborda	2020	Foreign Language Pre-Service Teachers' Attitudes Towards Integrated Technology	Journal Article	ICT Beliefs	Quantitative	Wiki/Blog	Language	Unspecified	Spain	Yes	No
Nair	2024	Knowledge, Attitude and Usage of Information and Communication Technology (ICT) and Digital Resources in Pre-Service Teachers	Journal Article	ICT Beliefs	Mixed	General	Unspecified	Unspecified	India	No	Yes
Mukmini	2023	Social media use for	Journal	ICT Beliefs	Quantitative	General	Language	Unspecified	Indonesi	Yes	Yes

n		English writing (SMU-EW): Preservice English teachers	Article						a		
Nandini	2018	A study on attitude towards using new technologies among B.Ed. student teachers in relation to few biographical variables	Journal Article	ICT Beliefs	Quantitative	General	Unspecified	Unspecified	India	Yes	No
Nelson	2020	The impact of field experiences on prospective preservice teachers' technology integration beliefs and intentions	Journal Article	ICT Beliefs Modeling	Mixed	General	Unspecified	Unspecified	United States	Yes	No
Nelson	2022	Coursework, field experiences, and the technology beliefs and practices of preservice teachers	Journal Article	ICT Beliefs	Quantitative	General	Mixed	Unspecified	United States	No	No
Olivares	2018	ICT in the classroom: Primary education student teachers' perceptions of the interactive whiteboard during the teaching practicum	Journal Article	ICT Beliefs	Qualitative	interactive whiteboard	Unspecified	Primary School	United States	No	No
Polly	2023	Examining Pre-Service and In-Service Teachers' Perceptions of Their Readiness to Use Digital	Journal Article	ICT Beliefs Comparison	Quantitative	General	Language	Unspecified	United States	Yes	No

		Technologies for Teaching and Learning									
Nasreen	2018	Perception of preservice teachers towards ICT integration in teacher education in India	Proceedings	ICT Beliefs	Quantitative	General	Unspecified	Unspecified	India	No	No
Prastikawati	2021	Pre-service EFL teachers' perception on technology-based formative assessment in their teaching practicum	Journal Article	Perception of ICT Based Teaching & Learning	Mixed	General	Language	Unspecified	Indonesia	No	No
Putra	2022	Prospective elementary teachers' attitude toward technology-based mathematics assessment	Proceedings	Perception of ICT Based Teaching & Learning	Qualitative	General	Math	Primary School	Indonesia	Yes	No
Ridha	2023	Efl pre-service teachers perception of technology integration in English language instruction	Journal Article	ICT Beliefs	Qualitative	General	Language	Unspecified	Indonesia	No	No
Pozas	2024	An empirical study exploring pre-service teachers' profiles and their prospective ICT integration: is it a matter of attitudes, self-efficacy, self-concept or concerns?	Journal Article	ICT Beliefs	Quantitative	General	Unspecified	Mixed	Germany	Yes	Yes
Rowston	2020	The lived experiences of career-change pre-service teachers and the promise of meaningful technology pedagogy	Journal Article	ICT Beliefs	Qualitative	General	Unspecified	Mixed	Australia	Yes	Yes

		beliefs and practice									
Sergeeva	2024	Exploring pre-service teachers' ICT competence beliefs	Journal Article	ICT Beliefs	Quantitative	General	Unspecified	Unspecified	Russia	Yes	No
Shater	2022	The Effect of Using Virtual Classrooms on the Attitudes of Student Teachers Toward the Use of Technology in Education	Proceedings	ICT Beliefs	Quantitative	General	Unspecified	Unspecified	United Arab Emirates	Yes	No
Rosidin	2019	Attitude towards technology for pre-service science teachers in Indonesia: An exploratory factor analysis	Journal Article	ICT Beliefs	Quantitative	General	Mixed	Unspecified	Indonesia	No	No
Štemberger	2021	Attitudes Towards Using Digital Technologies in Education as an Important Factor in Developing Digital Competence: The Case of Slovenian Student Teachers	Journal Article	ICT Beliefs	Quantitative	General	Unspecified	Unspecified	Slovenia	Yes	No
Tamang	2023	Information and communication Technology (ICT) infrastructure and Attitude of Pre-service Teachers towards Information and	Journal Article	ICT Beliefs	Quantitative	General	Unspecified	Primary School	India	Yes	No

		communication Technology (ICT)									
Turmuzi	2024	Perceptions of Primary School Teacher Education Students to the Use of ChatGPT to Support Learning in the Digital Era	Journal Article	ICT Beliefs	Mixed	ChatGPT	Unspecified	Unspecified	Indonesia	No	No
Üçgül	2023	The perceptions of prospective ICT teachers towards the integration of 3D printing into education and their views on the 3D modeling and printing course	Journal Article	ICT Beliefs	Qualitative	3D printing	Unspecified	Unspecified	Turkey	Yes	Yes
Tomczyk	2020	Attitude to ICT and Self-Evaluation of Fluency in Using New Digital Devices, Websites and Software among Pre-Service Teachers	Journal Article	ICT Beliefs	Quantitative	New Media: New Digital Devices, Websites and Software	Unspecified	Unspecified	Poland	No	No
Yang	2023	Pre-service teachers' perceptions and intentions regarding the use of chatbots through statistical and lag sequential analysis	Journal Article	ICT Beliefs	Mixed	chatbots	Computer	Unspecified	China	No	Yes
Yimchang	2018	A Study on Early Childhood Teachers and Pre-service Teachers' Belief, Confidence, and	Journal Article	ICT Beliefs	Quantitative	General	Math	Pre-school	Thailand	No	No

		Using Technology in Teaching Mathematics for Early Childhood Students									
Shittu		CORRELATIONAL STUDY OF PRE-SERVICE SCIENCE TEACHERS TECHNOLOGY PREPAREDNESS, BELIEFS, ATTITUDE, AND INTENTION TOWARDS USE OF INFORMATION TECHNOLOGY FOR TEACHING: A CASE STUDY	Journal Article	Relationship Studies	Quantitative	General	Mixed	Unspecified	Nigeria	Yes	Yes
Lam, M. K.; Nguyen, M.; Lowe, R.; Nagarajan, S. V.; Lincoln, M.; Schaper, L. K.; Grain, H.;	2014	"I can do it": Does confidence and perceived ability in learning new ICT skills predict pre-service health professionals' attitude towards engaging in e-healthcare?	Journal Article	Correlation Studies	Qualitative	E-health	Health	Unspecified	Australia	No	No

Martin-Sanchez, F.											
Zambak, Vecihi S.; Tyminski, Andrew M.	2017	A Case Study on Specialised Content Knowledge Development with Dynamic Geometry Software: The Analysis of Influential Factors and Technology Beliefs of Three Pre-Service Middle Grades Mathematics Teachers	Journal Article	ICT Beliefs	Qualitative	Computer Program	Math	Unspecified	US	Yes	Yes
Efe, H??lya Aslan; Efe, Rifat; Y??cel, Sait	2016	A Comparison of Swiss and Turkish Pre-Service Science Teachers' Attitudes, Anxiety and Self-Efficacy Regarding Educational Technology	Journal Article	ICT Beliefs Comparison	Quantitative	General	Science	Unspecified	Turkey	Yes	Yes
Incik, Eylem Yalcin; Akay, Cenk	2017	A Comprehensive Analysis on Technopedagogical Education Competency and Technology Perception of Pre-Service Teachers: Relation, Levels and Views	Journal Article	ICT Beliefs	Mixed	General	Mixed	Mixed	Turkey	No	Yes
Milman, Natalie	2008	A Longitudinal Assessment of Teacher	Journal Article	Teacher ICT Training	Quantitative	General	Unspecified	Unspecified	US	Yes	No

B.; Molebash, Philip E.		Education Students' Confidence toward Using Technology		Program							
Teclehaimanot, Berhane; Mentzer, Gale; Hickman, Torey	2011	A Mixed Methods Comparison of Teacher Education Faculty Perceptions of the Integration of Technology into Their Courses and Student Feedback on Technology Proficiency	Journal Article	ICT Beliefs Comparison	Mixed	General	Math	Secondary School	US	Yes	Yes
Gunes, E.; Bahcivan, E.	2018	A mixed research-based model for pre-service science teachers' digital literacy: Responses to "which beliefs" and "how and why they interact" questions	Journal Article	Digital Literacy	Mixed	General	Science	Unspecified	Turkey	Yes	No
Koc, Mustafa; Bakir, Nesrin	2010	A Needs Assessment Survey to Investigate Pre-Service Teachers' Knowledge, Experiences and Perceptions about Preparation to Using Educational Technologies	Journal Article	ICT Beliefs	Mixed	General	Mixed	Mixed	US	Yes	Yes
Teo, Timothy	2010	A Path Analysis of Pre-Service Teachers' Attitudes to Computer	Journal Article	ICT Beliefs Modeling	Quantitative	General	Unspecified	Mixed	Singapore	Yes	Yes

		Use: Applying and Extending the Technology Acceptance Model in an Educational Context									
Freidhoff, Joseph R.; Dickson, W. Patrick	2009	A programmatic profile of the uses, skills, and beliefs of preservice teacher education students and their instructors regarding online technologies	Thesis	ICT Beliefs Comparison	Quantitative	Online Technologies	Mixed	Mixed	US	Yes	Yes
Sheeran, Linda Rae; Jordan, Patricia Lampher e	2003	A study of pre -service teachers' attitudes concerning the use of technology in the classroom	Thesis	ICT Beliefs Comparison	Mixed	General	Unspecified	Mixed	US	Yes	No
Lin, Cheng-Yao	2008	A Study of Pre-Service Teachers' Attitudes about Computers and Mathematics Teaching: The Impact of Web-Based Instruction	Journal Article	Teacher ICT Training Program	Quantitative	Website	Math	Primary School	US	Yes	No
Holland, D. D.; Piper, R. T.	2016	A Technology Integration Education (TIE) Model for Millennial Preservice Teachers: Exploring the Canonical Correlation Relationships Among	Journal Article	Relationship Studies	Quantitative	General	Unspecified	Mixed	US	No	No

		Attitudes, Subjective Norms, Perceived Behavioral Controls, Motivation, and Technological, Pedagogical, and Content Knowledge (TPACK) Competencies									
Albion, P. R.; Jamieson -Proctor, R.; Finger, G.	2011	Age-related differences in ICT access and confidence among pre-service teachers	Proceeding s	Technology Competence	Quantitative	General	Unspecified	Unspecified	Australia	No	No
Teo, Timothy; Noyes, Jan	2011	An Assessment of the Influence of Perceived Enjoyment and Attitude on the Intention to Use Technology among Pre-Service Teachers: A Structural Equation Modeling Approach	Journal Article	ICT Beliefs Modeling	Quantitative	General	Unspecified	Higher Education	Singapor e	Yes	Yes
Ciftci, Serdar; Aladag, Soner	2018	An Investigation of Pre-Service Primary School Teachers' Attitudes towards Digital Technology and Digital Citizenship Levels in Terms of Some Variables	Journal Article	Correlation Studies	Quantitative	General	Unspecified	Primary School	Turkey	Yes	No
Karatas,	2017	An Investigation of	Journal	ICT Beliefs	Quantitative	General	Math	Primary	Turkey	No	Yes

I.; Tunc, M. P.; Yilmaz, N.; Karaci, G.		Technological Pedagogical Content Knowledge, Self-Confidence, and perceptio of pre-Service Middle School Mathematics Techaers towards Instructinoal Technologies	Article					School			
Zhang, Chenfen g; Koontz, Franklin	2001	An investigation of traditional and constructivist models for Internet training and attitudes of pre -service teachers	Thesis	Teacher ICT Training Program	Quantitative	Internet	Unspecified	Unspecified	US	Yes	No
Balcin, M. D.; Ari, E.; Erdogan, Y.; Besoluk, S.	2014	Analysis of self-efficacy perception of the science teacher candidates intended for information technology: Example of Sakarya University faculty of education	Journal Article	Technology Competence	Quantitative	General	Science	Unspecified	Turkey	No	No
Koksal, Mustafa Serdar; Yaman, Suleyma n; Saka, Yavuz	2016	Analysis of Turkish Prospective Science Teachers' Perceptions on Technology in Education	Journal Article	ICT Beliefs	Quantitative	General	Science	Unspecified	Turkey	Yes	Yes
Kabadayi ,	2006	Analyzing Pre-School Student Teachers' and	Journal Article	ICT Beliefs	Quantitative	General	Unspecified	Pre-school	Turkey	No	No

Abdulkadir		Their Cooperating Teachers' Attitudes towards the Use of Educational Technology									
Browne, Jeremy	2009	Assessing Pre-Service Teacher Attitudes and Skills with the Technology Integration Confidence Scale	Journal Article	Technology Competence	Quantitative	General	Unspecified	Unspecified	US	No	No
Peterson, Sharon L.	2010	Assessing Problem Solving Strategy Differences within Online and Face-to-Face Courses and Their Relationship to Pre-Service Teachers' Competence and Confidence for Integrating Technology into Teaching	Thesis	Relationship Studies	Quantitative	General	Unspecified	Primary School	US	No	No
Tuncer, M.; Kaysi, F.	2014	Assessing the perceptions of prospective teachers related to computer skills according to various variables	Journal Article	Technology Competence	Quantitative	Computer	Science	Unspecified	Turkey	No	No
Onder, F.; Celik, P.; Silay, I.; Karahoca	2011	Attitude of Teacher Candidates toward Making Computer Supported Education	Journal Article	Perception of ICT Based Teaching & Learning	Quantitative	Computer	Science	Unspecified	Turkey	No	No

, A.; Kanbul, S.											
Karr, Darci L.; Steckelberg, Allen	2014	Attitudes and beliefs student teachers hold toward technology integration	Thesis	ICT Beliefs	Quantitative	General	Unspecified	Primary School	US	No	No
Lyublinskaya, I.; Zhou, G.	2007	Attitudes of pre-service elementary teachers towards graphing calculator technologies in learning and teaching science	Manuscript	ICT Beliefs	Mixed	Graphing Calculator	Science	Primary School	US	No	No
Baz, Esra Harmandaoglu	2016	Attitudes of Turkish EFL Student Teachers towards Technology Use	Journal Article	ICT Beliefs	Mixed	General	Language	Unspecified	Turkey	No	Yes
Lin, Cheng-Yao	2008	Beliefs about Using Technology in the Mathematics Classroom: Interviews with Pre-Service Elementary Teachers	Journal Article	ICT Beliefs	Qualitative	General	Math	Primary School	US	No	No
Kim, Hoe Kyeung	2008	Beyond Motivation: ESL/EFL Teachers' Perceptions of the Role of Computers	Journal Article	ICT Beliefs	Qualitative	Computer	Language	Unspecified	US	Yes	No
Goktas, Yuksel; Demirel, Turgay	2012	Blog-Enhanced ICT Courses: Examining Their Effects on Prospective Teachers'	Journal Article	ICT Beliefs	Mixed	Blog	Social Studies	Primary School	Turkey	Yes	Yes

		ICT Competencies and Perceptions									
Sabiescu, A.; Van Zyl, I.; Pucciarelli, M.; Cantoni, L.; Bytheway, A.; Chigona, W.; Tardini, S.; International Development Research, Centre; Google,; Ibm,; Ipid,; Microsoft,	2013	Changing mindsets: The attitude of pre-service teachers on technology for teaching	Journal Article	Teacher ICT Training Program	Qualitative	Digital Storytelling	Unspecified	Unspecified	South Africa	No	Yes
Karatas, Ilhan	2014	Changing Pre-Service Mathematics Teachers' Beliefs about Using Computers for Teaching and Learning	Journal Article	Teacher ICT Training Program	Quantitative	Computer	Math	Mixed	Turkey	No	Yes

		Mathematics: The Effect of Three Different Models									
Barak, Miri	2014	Closing the Gap Between Attitudes and Perceptions About ICT-Enhanced Learning Among Pre-service STEM Teachers	Journal Article	Perception of ICT Based Teaching & Learning	Mixed	General	STEM	Unspecified	US	No	Yes
Lee, C. B.; Teo, T.; Chai, C. S.; Choy, D.; Tan, A.; Seah, J.; Blackboard,; E. M. C. Computer Systems; Echo,	2007	Closing the gap: Pre-service teachers' perceptions of an ICT based, student centred learning curriculum	Journal Article	Teacher ICT Training Program	Mixed	General	Unspecified	Unspecified	Singapore	No	No
Gomez, Jeannette R.; Garrett, Sherrye D.; Kouzekanani,	2016	Comparison of pre-service and in-service teachers' attitudes and perceived abilities toward integrating digital technologies into the classroom	Thesis	ICT Beliefs Comparison	Quantitative	General	Mixed	Mixed	US	Yes	No

Kamiar											
Can, S.; Uzunboy lu, H.	2010	Comparison of the attitudes of the pre-service teachers from SESMTE and SESSTE departments against computer use (The case of Mugla University, Turkey)	Journal Article	ICT Beliefs Comparison	Quantitative	Computer	Mixed	Secondary School	Turkey	Yes	No
Best, Linda M.; Tidwell, Monte G.	2002	Comparison of the perceptions of university faculty and pre -service students' technology skills and integration of technology in selected elementary education courses	Thesis	ICT Beliefs Comparison	Quantitative	General	Unspecified	Primary School	US	Yes	No
Bolandif ar, S.; Noordin, N.	2015	Computer anxiety and attitudes toward using internet in english language classes among iranian postgraduate student teachers	Journal Article	ICT Beliefs	Mixed	Internet	Language	Unspecified	Iran	Yes	No
Alawi, G. A. A. A.; Shwal, M.; Nasreen, N.; Dalian	2017	Computer usage & constructivist approach of the basic class: Abilities & skills and attitudes of pre-service teachers	Journal Article	ICT Beliefs	Quantitative	Computer	Mixed	Unspecified	Yemen	No	No

Maritime , Universit y											
Delaney, S.; Trapani, F.; Chandler , P.; Redman, C.; Chova, L. G.; Martinez, A. L.; Torres, I. C.	2014	CONTEMPORARY PRACTICES OF TECHNOLOGY AND ITS AFFORDANCES: PERCEPTIONS OF PRE-SERVICE TEACHERS ON THE UTILIZATION OF TECHNOLOGY IN TEACHING AND LEARNING PRACTICE	Proceeding s	ICT Beliefs	Qualitative	General	Science	Mixed	Australia	Yes	Yes
Yucel, A. S.; Kocak, C.; Uzunboy lu, H.; Cavus, N.	2009	Determination of attitudes of students teachers towards the utilization of technology: creating a technology tree	Journal Article	ICT Beliefs	Quantitative	General	Chemistry	Unspecified	Turkey	No	No
Ugras, Mustafa; Altunbas , Seda; Ay,	2012	Determination of the Pre-Service Science and Classroom Teachers' Attitudes Towards Science Teaching and	Journal Article	ICT Beliefs	Quantitative	General	Science	Unspecified	Turkey	No	No

Kemalettin; Cil, Erol		Technology and Relationship Between These Attitudes									
Krause, M.; Pietzner, V.; Dori, Y. J.; Eilks, I.	2017	Differences and developments in attitudes and self-efficacy of prospective chemistry teachers concerning the use of ICT in education	Journal Article	ICT Beliefs	Mixed	General	Chemistry	Unspecified	Germany	Yes	No
Dragon, Karon; Peacock, Kim; Norton, Yvonne; Steinhauer, Evelyn; Snart, Fern; Carbonaro, Mike; Boechler, Patricia	2012	Digital Opportunities within the Aboriginal Teacher Education Program: A Study of Preservice Teachers' Attitudes and Proficiency in Technology Integration	Journal Article	Teacher ICT Training Program	Mixed	Computer	Unspecified	Primary School	Canada	Yes	No
Asik, A.	2016	Digital Storytelling and Its Tools for Language Teaching: Perceptions and Reflections of Pre-Service Teachers	Journal Article	Teacher ICT Training Program	Mixed	Digital Storytelling	Language	Unspecified	Turkey	Yes	No
Tiba, C.; Condy,	2015	Digital storytelling as a tool for teaching:	Journal Article	ICT Beliefs	Qualitative	Digital Storytelling	Unspecified	Unspecified	South Africa	No	Yes

J.; Chigona, A.; Tunjera, N.		Perceptions of pre-service teachers									
Bumgarner, Barri L.	2012	Digital Storytelling in Writing: A Case Study of Student Teacher Attitudes toward Teaching with Technology	Thesis	ICT Beliefs	Qualitative	Digital Storytelling	Unspecified	Mixed	US	No	Yes
Funkhouser, Beverly J.; Mouza, Chrystalla	2013	Drawing on technology: An investigation of preservice teacher beliefs in the context of an introductory educational technology course	Journal Article	Teacher ICT Training Program	Qualitative	Drawing	Unspecified	Primary School	US	No	No
Yagci, M.	2016	Effect of information technologies (It) pre-service teachers' learning approaches on their attitude towards programing	Journal Article	ICT Beliefs	Quantitative	General	Science	Unspecified	Turkey	Yes	No
Kim, Dong-Joong; Choi, Sang-Ho	2016	Effects of a Technology-Friendly Education Program on Pre-Service Teachers' Perceptions and Learning Styles	Journal Article	Teacher ICT Training Program	Quantitative	General	Unspecified	Unspecified	South Korea	No	No
Sancar-Tokmak,	2015	Effects of Creating Digital Stories on	Journal Article	Teacher ICT Training	Mixed	Digital Storytelling	Language	Unspecified	Turkey	Yes	No

Hatice; Yanpar-Yelken, Tugba		Foreign Language Education Pre-Service Teachers' TPACK Self-Confidence		Program							
Smarkola, Claudia	2008	Efficacy of a planned behavior model: Beliefs that contribute to computer usage intentions of student teachers and experienced teachers	Journal Article	ICT Beliefs	Mixed	Computer	Unspecified	Mixed	US	Yes	Yes
Lee, Youngju; Lee, Jihyun	2014	Enhancing pre-service teachers' self-efficacy beliefs for technology integration through lesson planning practice	Journal Article	Teacher ICT Training Program	Quantitative	General	Mixed	Mixed	South Korea	No	No
Peterson, Leah; Jacobs, Howard	2015	Evaluating Pre-Service Teachers' Perceptions of Technology-Enhanced Field Experiences	Thesis	ICT Beliefs	Quantitative	General	Unspecified	Unspecified	US	No	No
Allsopp, David H.; McHatton, Patricia Alvarez; Cranston-Gingras, Ann	2009	Examining Perceptions of Systematic Integration of Instructional Technology in a Teacher Education Program	Journal Article	Teacher ICT Training Program	Mixed	General	Special Education	Unspecified	US	No	Yes
Teo,	2009	Examining the	Journal	Relationship	Quantitative	General	Unspecified	Unspecified	Singapor	Yes	Yes

Timothy		Relationship between Student Teachers' Self-Efficacy Beliefs and Their Intended Uses of Technology for Teaching: A Structural Equation Modelling Approach	Article	Studies					e		
Luan, Wong Su; Fung, Ng Siew; Nawawi, Mokhtar; Hong, Tang Sai	2005	Experienced and Inexperienced Internet Users among Pre-Service Teachers: Their Use and Attitudes toward the Internet	Journal Article	ICT Beliefs	Quantitative	General	Mixed	Unspecified	Malaysia	Yes	No
Redman, Christine ; Trapani, Fiona; Australia n Associati on for Research in, Educatio n	2012	Experiencing New Technology: Exploring Pre-Service Teachers' Perceptions and Reflections upon the Affordances of Social Media	Journal Article	ICT Beliefs	Quantitative	General	Unspecified	Unspecified	Australia	No	Yes
Teo, Timothy;	2010	Exploring Attitudes towards Computer Use	Journal Article	ICT Beliefs	Quantitative	Computer	Unspecified	Unspecified	Singapor e	No	No

Noyes, Jan		among Pre-Service Teachers from Singapore and the UK: A Multi-Group Invariance Test of the Technology Acceptance Model (TAM)									
Sadaf, Ayesha; Newby, Timothy J.; Ertmer, Peggy A.	2012	Exploring Pre-Service Teachers' Beliefs about Using Web 2.0 Technologies in K-12 Classroom	Journal Article	ICT Beliefs	Qualitative	Web 2.0	Unspecified	Mixed	US	Yes	Yes
Yaman, Ismail	2016	Exploring Prospective English Language Teachers' Perceptions of the "Internet" through Metaphorical Conceptualizations	Journal Article	ICT Beliefs	Qualitative	Internet	Language	Unspecified	Turkey	No	No
Bansilal, Sarah	2015	Exploring Student Teachers' Perceptions of the Influence of Technology in Learning and Teaching Mathematics	Journal Article	ICT Beliefs	Qualitative	General	Math	Unspecified	South Africa	No	Yes
Hsu, C. T.; Hsu, H. T.; Tsai, P. C.; Lee,	2012	Exploring Taiwanese pre-service fitness trainers' attitudes toward ICT and their teaching self-confidence	Journal Article	ICT Beliefs	Quantitative	General	Sports	Unspecified	Taiwan	No	Yes

M. H.												
Hakverdi, Meral; Gucum, Berna; Korkmaz, Hunkar	2007	Factors Influencing Pre-Service Science Teachers' Perception of Computer Self-Efficacy	Journal Article	Technology Competence	Quantitative	General	Science	Primary School	Turkey	Yes	Yes	
Su Luan, Wong; Atan, Hanafi	2007	Gender Differences in Attitudes towards Information Technology among Malaysian Student Teachers: A Case Study at Universiti Putra Malaysia	Journal Article	Teacher ICT Training Program	Quantitative	General	Unspecified	Unspecified	Malaysia	Yes	No	
Luan, W. S.; Fung, N. S.; Atan, H.	2008	Gender differences in the usage and attitudes toward the internet among student teachers in a public Malaysian university	Journal Article	ICT Beliefs	Quantitative	Internet	Unspecified	Unspecified	Malaysia	No	No	
Ho, H. J.; Choi, C. C.; Looi, C. K.; Jonassen, D.; Ikeda, M.	2005	Higher Order Applications of Technology: Perception of WebQuests among Pre-service Teachers	Journal Article	ICT Beliefs	Qualitative	WebQuests	Language	Mixed	Taiwan	No	No	
DuBay, Tracy Lynn;	2001	Impact of a verbal persuasion treatment on teacher education	Thesis	Teacher ICT Training Program	Quantitative	General	Unspecified	Primary School	US	No	Yes	

Gredler, Margaret		students' attitudes and self-efficacy for computer technology									
Huang, Chih-Hsun; McNeil, Sara	2003	Impact of socially desirable instrumentality and socially desirable expressiveness on female pre -service teachers' computer attitudes	Thesis	ICT Beliefs	Quantitative	General	Unspecified	Unspecified	US	No	Yes
Yuksel, G.; Kavanoz, S.; Karahoca, A.; Kanbul, S.	2011	In Search of Pre-Service EFL Certificate Teachers' Attitudes towards Technology	Journal Article	ICT Beliefs	Quantitative	General	Language	Unspecified	Turkey	Yes	No
Wishart, J.; Ward, R.	2002	Individual differences in nurse and teacher training students' attitudes toward and use of information technology	Journal Article	ICT Beliefs Comparison	Quantitative	Computer	Unspecified	Unspecified	UK	Yes	No
Fesakis, G.; Serafeim, K.; Acm,	2009	Influence of the Familiarization with "Scratch" on Future Teachers' Opinions and Attitudes about Programming and ICT in Education	Journal Article	ICT Beliefs	Quantitative	Computer Programming	Unspecified	Pre-school	Greece	No	No
Fredrick,	2013	INTEGRATING	Proceeding	ICT Beliefs	Quantitative	General	Unspecified	Unspecified	Uganda	No	No

S.; Andrew, L.; Chova, L. G.; Martinez, A. L.; Torres, I. C.		LEARNING TECHNOLOGY INTO THE CLASSROOM: THE IMPORTANCE OF PRE-SERVICE TEACHERS' AND LECTURERS' PERCEPTIONS	s								
Hornung, Claire Smith; Moe, Alden	2002	Integrating technology into preservice teacher education programs: A study of preparedness, attitudes, and self - efficacy	Thesis	ICT Beliefs	Quantitative	Computer	Unspecified	Unspecified	US	Yes	No
Yilmaz, Nursel; Alici, Sule	2011	Investigating Pre-Service Early Childhood Teachers' Attitudes towards the Computer Based Education in Science Activities	Journal Article	ICT Beliefs	Quantitative	Computer	Science	Pre-school	Turkey	Yes	No
Cobanoglu, I.; Ates, A.; Ilic, U.; Yilmaz, E.; Uzunboylu, H.; Cavus, N.	2009	Investigating prospective computer teachers' perceptions on e-learning	Journal Article	Perception of ICT Based Teaching & Learning	Quantitative	General	Computer	Unspecified	Turkey	No	No

Birkollu, S. S.; Yucesoy, Y.; Baglama, B.; Kanbul, S.	2017	Investigating the attitudes of pre-service teachers towards technology based on various variables	Journal Article	ICT Beliefs	Quantitative	General	Mixed	Unspecified	Turkey	Yes	No
Can, Suleyman	2015	Investigation of Pre-Service Physical Education Teachers' Attitudes Towards Computer Technologies (Case of Turkey)	Journal Article	ICT Beliefs	Quantitative	Computer	Sports	Unspecified	Turkey	No	No
Duru, A.; Peker, M.; Birgin, O.	2012	Investigation of pre-service teachers' attitude s toward using the computer in teaching and learning mathematics	Journal Article	ICT Beliefs	Quantitative	Computer	Math	Primary School	Turkey	Yes	No
Gok, B.; Erdogan, T.	2010	INVESTIGATION OF PRE-SERVICE TEACHERS' PERCEPTIONS ABOUT CONCEPT OF TECHNOLOGY THROUGH METAPHOR ANALYSIS	Journal Article	ICT Beliefs	Qualitative	General	Unspecified	Primary School	Turkey	No	No
Kaya, Sinan; Durmus,	2011	Investigation Of Relationship Between Preservice Teachers'	Journal Article	ICT Beliefs	Quantitative	Internet	Science	Primary School	Turkey	Yes	No

Alpaslan		Unethical Computer Using Behavior And Attitudes Towards The Using Of Internet									
Kuo, Ming-Mu	2008	Learner to Teacher: EFL Student Teachers' Perceptions on Internet-Assisted Language Learning and Teaching	Journal Article	Perception of ICT Based Teaching	Quantitative	Internet	Language	Unspecified	Taiwan	Yes	Yes
Robertson, Ian	2008	Learners' Attitudes to Wiki Technology in Problem Based, Blended Learning for Vocational Teacher Education	Journal Article	ICT Beliefs	Quantitative	Wiki	Mixed	Unspecified	Australia	Yes	Yes
Li, Kai Ming	2015	Learning Styles and Perceptions of Student Teachers of Computer-Supported Collaborative Learning Strategy Using Wikis	Journal Article	ICT Beliefs	Mixed	Wiki	Unspecified	Unspecified	Hong Kong	No	No
Wong, S. L.; Ng, S. F.; Tang, S. H.; Looi, C. K.; Jonassen, D.; Ikeda, M.	2005	Malaysian Female Pre-service Teachers Online: Exploring Their Internet Use and Attitudes	Journal Article	ICT Beliefs	Quantitative	Internet	Unspecified	Unspecified	Malaysia	Yes	No
Prasojo, Lantip	2017	Managing Digital Learning Environments:	Journal Article	ICT Beliefs	Mixed	Social Media	Language	Unspecified	Indonesia	Yes	No

Diat; Habibi, Akhmad; Mukminin, Amirul; Muhaimin,; Taridi, Muhammad; Ikhsan,; Saudagar, Ferdiaz		Student Teachers' Perception on the Social Networking Services Use in Writing Courses in Teacher Education									
Teo, Timothy	2010	Measuring the Effect of Gender on Computer Attitudes among Pre-Service Teachers: A Multiple Indicators, Multiple Causes (MIMIC) Modeling	Journal Article	ICT Beliefs Modeling	Quantitative	Computer	Unspecified	Unspecified	Singapore	Yes	No
Gungoren, O. C.; Horzum, M. B.	2015	Modeling pre-service teachers' perception of future internet usage for professional educational purposes	Journal Article	ICT Beliefs Modeling	Quantitative	Internet	Mixed	Mixed	Turkey	Yes	No
Teo, Timothy; Milutinovi??, Verica;	2016	Modelling Serbian pre-service teachers' attitudes towards computer use: A SEM and MIMIC approach	Journal Article	ICT Beliefs Modeling	Quantitative	Computer	Math	Primary School	Serbia	Yes	Yes

Zhou, Mingming											
Teo, Timothy	2012	Modelling the Influences of Beliefs on Pre-Service Teachers' Attitudes towards Computer Use	Journal Article	ICT Beliefs Modeling	Quantitative	Computer	Unspecified	Unspecified	Singapore	No	No
Fluck, A.; Dowden, T.	2013	On the Cusp of Change: Examining Pre-Service Teachers' Beliefs about ICT and Envisioning the Digital Classroom of the Future	Journal Article	ICT Beliefs	Mixed	Computer	Unspecified	Unspecified	Australia	Yes	Yes
Chisalita, O.; Cretu, C.; Frunzeti, T.; Jugureanu, R.; Ciolan, L.; Radu, C.	2012	OPINIONS AND ATTITUDES OF STUDENTS TEACHERS' TOWARD ICT USE IN EDUCATION	Proceedings	ICT Beliefs	Quantitative	General	Unspecified	Mixed	Rome	No	No
Chiou, Yu-Fang; Franklin, Teresa	2011	Perceived Usefulness, Perceive Ease of Use, Computer Attitude, and Using Experience of Web 2.0 Applications as Predictors of Intent to Use Web 2.0 by Pre-	Thesis	ICT Beliefs	Quantitative	Web 2.0	Mixed	Mixed	US	Yes	No

		service Teachers for Teaching									
Friedman, Adam; Kajder, Sara	2006	Perceptions of Beginning Teacher Education Students regarding Educational Technology	Journal Article	ICT Beliefs	Qualitative	General	Mixed	Mixed	US	No	No
Elstad, Eyvind; Christophersen, Knut-Andreas	2017	Perceptions of Digital Competency among Student Teachers: Contributing to the Development of Student Teachers' Instructional Self-Efficacy in Technology-Rich Classrooms	Journal Article	Technology Competence	Quantitative	General	Mixed	Unspecified	Norway	No	Yes
Akbulut, Y.; Odabasi, H. F.; Kuzu, A.	2011	PERCEPTIONS OF PRESERVICE TEACHERS REGARDING THE INTEGRATION OF INFORMATION AND COMMUNICATION TECHNOLOGIES IN TURKISH EDUCATION FACULTIES	Journal Article	ICT Beliefs	Quantitative	General	Unspecified	Unspecified	Turkey	Yes	No
Kaya, K. Y.; Tisoglu, S.; Ucak, S. S. K.;	2012	PERCEPTIONS OF PROSPECTIVE INFORMATION TECHNOLOGIES TEACHERS TOWARDS	Journal Article	ICT Beliefs	Mixed	Mixed	Computer	Unspecified	Turkey	Yes	No

Kadioglu, E. A.; Chova, L. G.; Torres, I. C.; Martinez, A. L.		FATIH PROJECT AND ITS COMPONENTS									
Eyyam, R.; Menevis, I.; Dogruer, N.; Osam, U. V.; Vefali, G. M.; Kufi, E. O.; Arkin, E. I.	2010	Perceptions of prospective teachers towards technology use in class	Journal Article	ICT Beliefs	Quantitative	General	Unspecified	Unspecified	North Cyprus	Yes	No
Greene-Clemons, Cheresa Denae	2016	Perceptions of Technology Engagement on Culturally Responsive Pre-Service Teachers	Journal Article	ICT Beliefs	Mixed	General	Unspecified	Mixed	US	Yes	Yes
Shoffner, Melanie	2009	Personal Attitudes and Technology: Implications for Preservice Teacher Reflective Practice	Journal Article	ICT Beliefs	Qualitative	Blog	Mixed	Unspecified	US	No	Yes
Varol, Yaprak	2015	Predictive Power of Prospective Physical	Journal Article	ICT Beliefs	Quantitative	General	Sports	Unspecified	Turkey	No	No

Kalemoglu		Education Teachers' Attitudes towards Educational Technologies for Their Technological Pedagogical Content Knowledge									
Lin, Y. H.; Liang, J. C.; International Institute of Applied, Informatics; Kyushu, University; Res. Inst. Inf. Technol. Kyushu, Univ	2012	Preschool teachers' internet attitude and their internet self-efficacy: A comparative study between pre-service and in-service teachers in taiwan	Journal Article	ICT Beliefs Comparison	Quantitative	Internet	Unspecified	Pre-school	Taiwan	No	No
Lu, Li-Fen Lilly	2005	Pre-Service Art Teacher Negative Attitudes and Perceptions of Computer-Generated Art Imagery: Recommendations for	Journal Article	ICT Beliefs	Mixed	Computer Generated Art Imagery	Art	Unspecified	US	No	Yes

		Pre-Service Art Education Programs									
Yapici, I. U.; Hevedanli, M.; Isman, A.; Liu, E. Z. F.; Kiyici, M.	2012	Pre-Service Biology Teachers' Attitudes towards ICT Using In Biology Teaching	Journal Article	ICT Beliefs	Quantitative	General	Biology	Unspecified	Turkey	No	Yes
Zhou, Qing; Zhao, Yingmin; Hu, Jiani; Liu, Yang; Xing, Lijuan	2010	Pre-service chemistry teachers' attitude toward ICT in Xi'an	Journal Article	ICT Beliefs	Quantitative	General	Chemistry	Unspecified	China	No	No
Basoz, T.; Cubukcu, F.; Laborda, J. C.; Ozdamli, F.; Maasoglu, Y.	2014	Pre-service EFL teachers' attitudes towards Computer Assisted Language Learning (CALL)	Journal Article	Perception of ICT Based Teaching & Learning	Quantitative	Computer	Language	Unspecified	Turkey	No	No

Blakeney, Kimberly P.; Larson, Mark L.; Warren, Elizabeth	2014	Pre-service elementary education teachers self-efficacy beliefs regarding technology integration in the classroom environment: A case study	Thesis	Technology Competence	Qualitative	General	Unspecified	Primary School	US	No	Yes
Acikalin, Mehmet	2009	Pre-Service Elementary Teachers' Beliefs about Use of the Internet in the Social Studies Classroom	Journal Article	ICT Beliefs	Quantitative	Computer	Social Studies	Primary School	Turkey	Yes	No
Saricoban, A.	2013	Pre-Service ELT Teachers' Attitudes Towards Computer Use: A Turkish Survey	Journal Article	ICT Beliefs	Quantitative	Computer	Language	Unspecified	Turkey	Yes	No
Topkaya, Ece Zehir	2010	Pre-Service English Language Teachers' Perceptions of Computer Self-Efficacy and General Self-Efficacy	Journal Article	Technology Competence	Quantitative	Computer	Language	Unspecified	Turkey	Yes	No
Abdul Latiff, A.; Mat Daud, N.	2013	Pre-Service ESL teachers' perceptions of parody integration in digital stories	Journal Article	ICT Beliefs	Quantitative	Digital Storytelling	Language	Unspecified	Malaysia	Yes	No
Lee, Robert Earl; McLean, Robert S.	2003	Pre-service geography teachers' self perceptions of readiness for technology implementation	Thesis	Technology Competence	Mixed	General	Geography	Unspecified	Canada	Yes	Yes
Zambak,	2014	Pre-service mathematics	Thesis	Teacher ICT	Mixed	General	Math	Unspecified	US	Yes	Yes

Vecihi Serbay; Tyminski , Andrew M.		teachers' knowledge development and belief change within a technology-enhanced mathematics course		Training Program							
Wegis, Heidi M.	2008	Pre-service physical education teachers' attitudes toward, and use of, handheld technology	Thesis	ICT Beliefs	Mixed	PDA	Sports	Unspecified	US	Yes	Yes
Bursal, Murat; Yigit, Nevzat	2012	Pre-Service Science and Technology Teachers' Efficacy Beliefs about Information and Communication Technologies (ICT) Usage and Material Design	Journal Article	Technology Competence	Quantitative	General	Unspecified	Unspecified	Turkey	No	No
Ozkan, G.; Tombak, B.	2015	Pre-service science teachers' perceptions of technology literacy	Journal Article	Digital Literacy	Qualitative	General	Science	Unspecified	Turkey	No	No
Condy, Janet; Chigona, Agnes; Gachago, Daniela; Ivala, Eunice; Chigona, Agnes	2012	Pre-Service Students' Perceptions and Experiences of Digital Storytelling in Diverse Classrooms	Journal Article	ICT Beliefs	Qualitative	Digital Storytelling	Unspecified	Unspecified	Turkey	No	Yes

Gyamfi, Stephen Adu	2017	Pre-Service Teachers' Attitude towards Information and Communication Technology Usage: A Ghanaian Survey	Journal Article	ICT Beliefs	Quantitative	General	Unspecified	Unspecified	Ghana	Yes	Yes
Teo, Timothy	2008	Pre-Service Teachers' Attitudes towards Computer Use: A Singapore Survey	Journal Article	ICT Beliefs	Quantitative	Computer	Mixed	Mixed	Singapor e	Yes	No
Porfilio, Bradley J.; Bromley, Hank	2005	Pre-service teachers' beliefs and experiences with computing technology and male centered computing culture	Thesis	ICT Beliefs	Qualitative	Computing Technologie s	Unspecified	Unspecified	US	No	Yes
Park, Yong Joon; Yang, Youjin	2013	Pre-Service Teachers' Perception of and Technology Competency at Creating and Using E-Picture Books	Journal Article	ICT Beliefs	Qualitative	E-Picture Books	Mixed	Primary School	US	Yes	No
Baltaci-Goktalay, Sehnaz; Ozdilek, Zehra	2010	Pre-service teachers' perceptions about web 2.0 technologies	Journal Article	ICT Beliefs	Quantitative	Web 2.0	Science	Unspecified	Turkey	Yes	No
Aslan, Aydin; Zhu, Chang	2015	Pre-Service Teachers' Perceptions of ICT Integration in Teacher Education in Turkey	Journal Article	Teacher ICT Training Program	Mixed	General	Mixed	Unspecified	Turkey	Yes	Yes
Garc??a-	2017	Pre-service teachers'	Journal	Technology	Quantitative	General	Mixed	Secondary	Spain	Yes	No

Mart??n, Judit; Garc??a-S??nchez , Jes??s-Nicasio		perceptions of the competence dimensions of digital literacy and of psychological and educational measures	Article	Competence				School			
Inoue-Smith, Yukiko	2014	Pre-Service Teachers' Perceptions of the Internet and Online Courses: The Case of an American Pacific Island University	Journal Article	ICT Beliefs	Quantitative	Internet	Unspecified	Unspecified	US	Yes	No
Raphael, Christina ; Mtebe, Joel S.	2017	Pre-Service Teachers' Self-Efficacy Beliefs towards Educational Technologies Integration in Tanzania	Journal Article	Technology Competence	Quantitative	General	Mixed	Unspecified	Tanzania	Yes	No
Zyad, Hicham	2016	Pre-Service Training and ICT Implementation in the Classroom: ELT Teachers' Perceptions	Journal Article	Teacher ICT Training Program	Quantitative	General	Language	Mixed	Morocco	Yes	Yes
Burnett, Catherin e M.	2009	Primary student-teachers' perceptions of the role of digital literacy in their lives	Thesis	Digital Literacy	Mixed	General	Language	Primary School	UK	Yes	Yes
Hismano glu, Murat	2012	Prospective EFL Teachers' Perceptions of ICT Integration: A Study of Distance Higher Education in Turkey	Journal Article	ICT Beliefs	Mixed	General	Language	Unspecified	Turkey	Yes	Yes
Hazzan,	2002	Prospective high school	Journal	ICT Beliefs	Qualitative	Computer	Math	Secondary	Israel	No	No

O.		mathematics teachers' attitudes toward integrating computers in their future teaching	Article					School			
Ipek, A. Sabri; Berigel, Muhammed; Albayrak, Mustafa	2007	Prospective Mathematics Teachers' Attitudes Towards Learning Mathematics with Technology	Journal Article	ICT Beliefs	Quantitative	General	Math	Primary School	Turkey	No	No
Kobak, Mevhibe; Taskin, Nazli Ruya	2012	Prospective Teachers' Perceptions of using Technology in Three Different Ways	Journal Article	ICT Beliefs	Mixed	General	Science	Secondary School	Turkey	Yes	Yes
Yeung, Alexander Seeshing; Lim, Kam Ming; Tay, Eng Guan; Lam-Chiang, Audrey Cheausim; Hui,	2012	Relating Use of Digital Technology by Pre-Service Teachers to Confidence: A Singapore Survey	Journal Article	Technology Competence	Mixed	General	Unspecified	Unspecified	Singapore	Yes	No

Chenri											
Jamieson -Proctor, Romina; Finger, Glenn	2006	Relationship between Pre-Service and Practising Teachers' Confidence and Beliefs about Using ICT	Journal Article	Relationship Studies	Quantitative	General	Mixed	Mixed	Australia	Yes	No
Akkaya, Recai	2016	Research on the Development of Middle School Mathematics Pre-Service Teachers' Perceptions Regarding the Use of Technology in Teaching Mathematics	Journal Article	Teacher ICT Training Program	Mixed	General	Math	Primary School	Turkey	No	Yes
Alblaihe d, Munthir Abdullah	2016	Saudi arabian science and mathematics pre-service teachers' perceptions and practices of the integration of technology in the classroom	Thesis	ICT Beliefs	Mixed	General	Science	Unspecified	Saudi Arabia	Yes	Yes
Chai, C. S.; Hong, H. Y.; Teo, T.	2009	Singaporean and Taiwanese pre-service teachers' beliefs and their attitude towards ICT use: A comparative study	Journal Article	ICT Beliefs Comparison	Quantitative	Computer	Mixed	Unspecified	Singapor e	Yes	No
Chai, Ching Sing; Wong, Benjami n; Teo,	2011	Singaporean Pre-Service Teachers' Beliefs about Epistemology, Teaching and Learning, and Technology	Journal Article	ICT Beliefs	Qualitative	General	Unspecified	Mixed	Singapor e	No	No

Timothy											
Albion, Peter R.	2001	Some Factors in the Development of Self-Efficacy Beliefs for Computer Use among Teacher Education Students	Journal Article	Teacher ICT Training Program	Quantitative	Computer	Unspecified	Mixed	Australia	Yes	Yes
Assaf, L. C.	2005	Staying connected: Student teachers' perceptions of computer-mediated discussions	Journal Article	ICT Beliefs	Qualitative	Online Discussion Board	Unspecified	Primary School	US	Yes	No
Tur, G.; Marin, V. I.; GarciaPenalvo, F. J.	2013	Student Teachers' Attitude towards ePortfolios and Technology in Education	Journal Article	ICT Beliefs	Quantitative	ePortfolios	Unspecified	Mixed	Spain	No	No
Beacham, N.; McIntosh, K.	2014	Student teachers' attitudes and beliefs towards using ICT within inclusive education and practice	Journal Article	ICT Beliefs	Quantitative	General	Unspecified	Unspecified	UK	Yes	Yes
Mc Alister, M.; Dunn, J.; Quinn, L.	2005	Student teachers' attitudes to and use of computers to teach mathematics in the primary classroom	Journal Article	ICT Beliefs	Quantitative	Computer	Math	Primary School	UK	Yes	No
Chang, Y. F.; Chen, Y.	2012	Student teachers' perception of the VBL system to enhance	Proceedings	Technology Competence	Mixed	VBL	Mixed	Unspecified	Taiwan	No	No

C.; Hsu, C. L.; Croucher, Foundation; Sino-British Fellowship, Trust; Wu Jieh Yee Charitable, Foundation		technology integration competencies									
Srisawasdi, Niwat	2012	Student Teachers' Perceptions of Computerized Laboratory Practice For Science Teaching: A Comparative Analysis	Journal Article	ICT Beliefs	Quantitative	Science Lab	Science	Secondary School	Thailand	No	Yes
Almeida, C.; Morais, C.; Miranda, L.; Viseu, F.; Martinho, H.	2002	Student teachers' perceptions of the Internet: Motivation, influences, and use	Journal Article	ICT Beliefs	Quantitative	Internet	Science	Mixed	Portugal	Yes	No
Orhan	2018	Student Teachers'	Journal	ICT Beliefs	Qualitative	General	Unspecified	Unspecified	Turkey	Yes	No

Goksun, Derya; Filiz, Ozan; Kurt, Adile Askim		Perceptions on Educational Technologies' Past, Present and Future	Article								
Ekizoglu, N.; Tezer, M.; Bozer, M.; Keser, H.; Ozcinar, Z.; Kanbul, S.	2010	Teacher candidates' real success situation on computers and their attitudes towards computer technology in the faculties of education	Journal Article	ICT Beliefs	Quantitative	Computer	Mixed	Mixed	Turkey	Yes	Yes
Yilmazel-Sahin, Yesim; Oxford, Rebecca L.	2010	Teacher Education Students' Perceptions of the Value of Handouts Accompanying Teacher Educators' Computer-Generated Slide Presentations	Journal Article	ICT Beliefs	Mixed	Slides	Mixed	Unspecified	US	Yes	No
Svensson, M.; Baelo, R.; Soare,	2015	Teacher students' perceptions of their digital competence	Proceedings	Technology Competence	Quantitative	General	Unspecified	Unspecified	Sweden	No	No

E.; Langa, C.											
Quadrini, Virginia Horak	2013	Teacher-Education Student Perceptions for Stages of Concern Related to Integrating Technology	Thesis	ICT Beliefs	Quantitative	General	Unspecified	Unspecified	US	Yes	Yes
Moham mad, Anwar H.; Marshall, J. Daniel	2004	Teaching and learning with technology: Kuwaiti mathematics pre -service teachers' competencies and attitudes	Thesis	Teacher ICT Training Program	Quantitative	General	Math	Unspecified	Kuwait	Yes	No
Jung, Eun Joo; Rhodes, Dent M.	2004	Technology disposition of teacher education students: Beliefs, attitudes, self-concepts, and competence	Thesis	ICT Beliefs	Quantitative	General	Unspecified	Unspecified	US	Yes	No
Rehmat, Abeera P.; Bailey, Janelle M.	2014	Technology Integration in a Science Classroom: Preservice Teachers' Perceptions	Journal Article	ICT Beliefs	Qualitative	General	Science	Primary School	US	Yes	No
Can, S.	2016	The attitudes of pre-service teachers attending the schools of physical education and sports of the universities in Turkey towards	Journal Article	ICT Beliefs	Quantitative	General	Sports	Unspecified	Turkey	Yes	No

		education technologies									
Tezer, Murat; Bicen, H??seyin	2009	The attitudes of prospective teachers' on trusting internet resources	Proceedings	ICT Beliefs	Quantitative	Internet	Mixed	Mixed	North Cyprus	Yes	No
Alkan, Fatma; Erdem, Emine	2010	The attitudes of student teachers towards educational technologies according to their status of receiving teaching application lessons	Journal Article	ICT Beliefs	Quantitative	General	Science	Unspecified	Turkey	Yes	No
Findikoglu, F.; Alci, B.; Karatas, H.	2015	The correlation between pre-service teachers‘ attitudes towards technology and achievement in material design course	Journal Article	Relationship Studies	Quantitative	General	Unspecified	Unspecified	Turkey	No	Yes
Sancar Tokmak, Hatice; Ozgelen, Sinan	2013	The ECE Pre-Service Teachers' Perception on Factors Affecting the Integration of Educational Computer Games in Two Conditions: Selecting versus Redesigning	Journal Article	ICT Beliefs	Qualitative	Computer Games	Unspecified	Pre-school	Turkey	Yes	No
Tatar, Enver	2013	The Effect of Dynamic Software on Prospective Mathematics Teachers' Perceptions Regarding Information and Communication	Journal Article	Teacher ICT Training Program	Mixed	Computer Program	Math	Secondary School	Turkey	Yes	No

		Technology									
van 't Hooft, Mark A.; Crowe, Alicia R.	2005	The effect of handheld technology use in pre - service social studies education on the attitudes of future teachers toward technology integration in social studies	Thesis	ICT Beliefs	Quantitative	Handheld Technology	Mixed	Secondary School	US	Yes	No
Han, Insook; Shin, Won Sug; Ko, Yujung	2017	The Effect of Student Teaching Experience and Teacher Beliefs on Pre-Service Teachers' Self-Efficacy and Intention to Use Technology in Teaching	Journal Article	Teacher ICT Training Program	Quantitative	General	Unspecified	Secondary School	South Korea	No	No
Al-Awidi, Hamed Mubarak ; Alghazo, Iman Mohammad	2012	The effect of student teaching experience on preservice elementary teachers' self-efficacy beliefs for technology integration in the UAE	Journal Article	Teacher ICT Training Program	Mixed	General	Unspecified	Primary School	United Arab Emirates	No	Yes
Angeli, Charoula	2004	The Effects of Case-Based Learning on Early Childhood Pre-Service Teachers' Beliefs about the Pedagogical Uses of ICT	Journal Article	Teacher ICT Training Program	Qualitative	General	Unspecified	Pre-school	Cyprus	No	No
Oral,	2008	The Evaluation of the	Journal	ICT Beliefs	Quantitative	Internet	Unspecified	Unspecified	Turkey	No	No

Behcet		Student Teachers' Attitudes toward Internet and Democracy	Article								
Hayes, Elisabeth ; Ohrnberger, Maryellen	2013	The Gamer Generation Teaches School: The Gaming Practices and Attitudes towards Technology of Pre-Service Teachers	Journal Article	ICT Beliefs	Quantitative	Computer Games	Unspecified	Unspecified	US	No	Yes
Hismanoglu, Murat	2012	The Impact of a Curricular Innovation on Prospective EFL Teachers' Attitudes towards ICT Integration into Language Instruction	Journal Article	Teacher ICT Training Program	Mixed	General	Unspecified	Unspecified	Turkey	Yes	Yes
Giles, Linda Michelle; Tyler-Wood, Tandra	2016	The impact of a paired grouping pre-service technology integration course on student participant attitudes, proficiency, and technological knowledge toward technology	Thesis	Teacher ICT Training Program	Mixed	General	Unspecified	Unspecified	US	Yes	Yes
Stonier, Francis W.	2012	The Impact of an Intensive Experience on Prospective Teachers' Perception of the Uses of Digital, Interactive Text among K-12 Students	Thesis	Teacher ICT Training Program	Mixed	Digital Text	Unspecified	Unspecified	US	Yes	No

Banas, Jennifer R.; York, Cindy S.	2014	The Impact of Authentic Learning Exercises on Pre-Service Teachers' Motivational Beliefs towards Technology Integration	Journal Article	Teacher ICT Training Program	Quantitative	General	Health	Unspecified	US	Yes	Yes
Adamy, Peter; Boulmetis, John	2006	The Impact of Modeling Technology Integration on Pre-Service Teachers' Technology Confidence	Journal Article	Technology Competence	Quantitative	General	Unspecified	Mixed	US	No	No
Alexander, Curby; Knezek, Gerald; Christensen, Rhonda; Tyler-Wood, Tandra; Bull, Glen	2014	The Impact of Project-Based Learning on Pre-Service Teachers' Technology Attitudes and Skills	Journal Article	Relationship Studies	Quantitative	General	Mixed	Mixed	US	No	No
Teo, Timothy	2009	The Impact of Subjective Norm and Facilitating Conditions on Pre-Service Teachers' Attitude toward Computer Use: A Structural Equation Modeling of an Extended Technology Acceptance	Journal Article	ICT Beliefs Modeling	Quantitative	General	Unspecified	Secondary School	Singapore	Yes	Yes

		Model									
Benson, Linda F.; Farnsworth, Briant J.; Bahr, Damon L.; Lewis, Valerie K.; Shaha, Steven H.	2004	The Impact of Training in Technology Assisted Instruction on Skills and Attitudes of Pre-Service Teachers	Journal Article	Teacher ICT Training Program	Mixed	General	Science	Primary School	US	No	No
Scherer, Ronny; Tondeur, Jo; Siddiq, Fazilat; Baran, Evrim	2018	The importance of attitudes toward technology for pre-service teachers' technological, pedagogical, and content knowledge: Comparing structural equation modeling approaches	Journal Article	ICT Beliefs Modeling	Quantitative	General	Unspecified	Unspecified	Belgium	No	No
Gakhar, Sonia; Thompson, Ann; Schmidt, Denise; Hegland, Susan	2007	The influence of a digital storytelling experience on pre-service teacher education students' attitudes and intentions	Thesis	Teacher ICT Training Program	Mixed	Digital Storytelling	Unspecified	Mixed	US	No	Yes

Yagci, M.; Sirakaya, D. A.; Ozudogru, G.; Iaman, A.; Eskicumali, A.	2015	The investigation of attitude and readiness of information and communication technologies pre-service teachers toward web based learning	Journal Article	Perception of ICT Based Teaching & Learning	Quantitative	General	Computer	Unspecified	Turkey	Yes	No
Constantin, P.; Sgem,	2014	THE PERCEPTION OF THE PHYSICAL EDUCATION TEACHERS CONCERNING THE USE OF THE AUDIOVISUAL TECHNOLOGIES IN SHAPING THE MOTOR SKILLS	Journal Article	ICT Beliefs	Mixed	Audio-visual Technology	Sports	Unspecified	Romania	Yes	Yes
Birinci, G.; Kilicer, K.; Uzunboylu, H.; Cavus, N.	2009	The pre-service teachers' competency perceptions regarding technology planning	Journal Article	Technology Competence	Quantitative	General	Computer	Unspecified	Turkey	Yes	Yes
I??ikg??z, E.	2016	The relation between the attitudes of pre-service physical education	Journal Article	Relationship Studies	Quantitative	General	Sports	Unspecified	Turkey	No	Yes

		teachers towards instructional technologies and material design course and their academic standing: Batman university sample									
Inayati, Dian; Emaliana , Ive	2017	The Relationship among Pre-Service EFL Teachers' Beliefs about Language Learning, Pedagogical Beliefs, and Beliefs about ICT Integration	Journal Article	Relationship Studies	Quantitative	General	Language	Unspecified	Indonesi a	No	No
Baturay, M. H.; G??k??e arslan, ?? ; Ke, F.	2017	The relationship among pre-service teachers' computer competence, attitude towards computer-assisted education, and intention of technology acceptance	Journal Article	Relationship Studies	Quantitative	Computer	Mixed	Unspecified	Turkey	Yes	Yes
Kalemog lu Varol, Yaprak	2014	The Relationship between Attitudes of Prospective Physical Education Teachers towards Education Technologies and Computer Self-Efficacy Beliefs	Journal Article	Relationship Studies	Quantitative	General	Physics	Unspecified	Turkey	Yes	No
Martinda le,	2015	The Relationship between Pre-Service	Thesis	Relationship Studies	Quantitative	iPad	Unspecified	Pre-school	US	Yes	Yes

Rebecca; Gartin, Barbara C.		Teachers' Basic Technology Competence, Technology Self-Efficacy and Perceptions of Adopting Educational Applications on iPads for Classroom Use									
Yang, Xinrong; Leung, Frederick K. S.	2015	The Relationships among Pre-Service Mathematics Teachers' Beliefs about Mathematics, Mathematics Teaching, and Use of Technology in China	Journal Article	Relationship Studies	Quantitative	General	Math	Primary School	China	No	No
Chang, Y. T.; Lai, Y. Z.; Hwang, B. C.; Hsu, Y. S.; Cantoni, L.; McLoughlin, C.	2004	The study on the pre-service teachers' attitudes and belief on the integration of infonnation technology in teaching	Journal Article	ICT Beliefs	Quantitative	General	Biology	Unspecified	Taiwan	No	No
Al-Hazza, Tami Craft	2017	The Tension in Pre-Service Teachers' Explorations of Tablet Technology for Literacy Purposes: Positive	Journal Article	ICT Beliefs	Mixed	Tablets	Unspecified	Unspecified	US	No	Yes

		Beliefs and Practical Shortcomings									
Keser, Hafize; Karaoglan Yilmaz, Fatma Gizem; Yilmaz, Ramazan	2015	TPACK Competencies and Technology Integration Self-Efficacy Perceptions of Pre-Service Teachers	Journal Article	Technology Competence	Quantitative	General	Mixed	Mixed	Turkey	Yes	No
Dogan, M.; Yang, W. C.; Majewski, M.; De Alwis, T.; Karakirk, E.	2011	TRAINEE TEACHERS' ATTITUDES ABOUT MATERIALS AND TECHNOLOGY USE IN MATHEMATICS EDUCATION	Journal Article	ICT Beliefs	Quantitative	General	Math	Primary School	Turkey	No	No
Best, Marnie	2017	Transforming Pre-Service Teachers' Beliefs and Understandings about Design and Technologies	Journal Article	Teacher ICT Training Program	Quantitative	General	Unspecified	Unspecified	Australia	No	Yes
Gurcay, D.; Wong, B.; Chai, C. S.	2013	Turkish and Singaporean Pre-service Physics Teachers' Beliefs about Teaching and Use of Technology	Journal Article	ICT Beliefs	Quantitative	General	Physics	Secondary School	Turkey	No	No
Pamuk,	2009	Turkish Pre-Service	Journal	ICT Beliefs	Quantitative	Computer	Mixed	Primary	Turkey	Yes	No

Savas; Peker, Deniz		Science and Mathematics Teachers' Computer Related Self-Efficacies, Attitudes, and the Relationship between These Variables	Article					School			
Can, Gulfidan; Cagiltay, Kursat	2006	Turkish Prospective Teachers' Perceptions regarding the Use of Computer Games with Educational Features	Journal Article	ICT Beliefs	Mixed	Computer Games	Science	Unspecified	Turkey	Yes	No
Luan, Wong Su; Bakar, Kamaria h Abu; Hong, Tang Sai	2006	Using a Student-Centred Learning Approach to Teach a Discrete Information Technology Course: The Effects on Malaysian Pre-Service Teachers' Attitudes toward Information Technology	Journal Article	Teacher ICT Training Program	Mixed	General	Unspecified	Unspecified	Malaysia	No	No
Efendiog lu, A.; Berkant, H. G.; Cukurov a, B.; Uzunboy lu, H.; Ozdamli, F.	2013	Using constructivist and collaborative approach to enhance pre-service teachers' attitude toward computer in computer course: Learning and using MS Excel functions in problem-based scenarios	Proceeding s	Teacher ICT Training Program	Quantitative	Computer	Mixed	Primary School	Turkey	No	No
Kahrama	2015	Using Digital	Journal	ICT Beliefs	Mixed	Dynamic	Science	Primary	Turkey	No	No

n, S.; Demir, Y.; Demir, N.		technology-generated dynamic visualization in science education- perceptions of pre-service science teachers	Article			Visualization		School			
Burkett, Ruth S.; Barron, Ann E.	2002	Using electronic bulletin boards and journals to enhance pre-service teachers self-efficacy and attitudes toward the use of computers	Thesis	Teacher ICT Training Program	Mixed	Computer	Unspecified	Unspecified	US	No	No
